# Supplementary material for: Poxviruses and paramyxoviruses use a conserved mechanism of STAT1 antagonism to inhibit interferon signaling
Source: Cell Host Microbe. 2022 Mar 9;30(3):357–372.e11. doi: 10.1016/j.chom.2022.01.014 (PMC8912257; doi:10.1016/j.chom.2022.01.014)
Supplement: Document S1. Figures S1–S7 and Tables S1–S5 [file mmc1.pdf]

Cell Host & Microbe, Volume *30*

Supplemental information

Poxviruses and paramyxoviruses use a conserved mechanism of STAT1 antagonism to inhibit interferon signaling

Callum Talbot-Cooper, Teodors Pantelejevs, John P. Shannon, Christian R. Cherry, Marcus T. Au, Marko Hyvönen, Heather D. Hickman, and Geoffrey L. Smith

Table S1. Oligonucleotide primers for construction of recombinant DNA. Related to STAR methods. Cloning sites are highlighted in bold (for restriction digest cloning) or underlined (for ligation-independent cloning). Complementary sequences for overlapping PCR are italicised. Site-directed mutagenesis primers are indicated with an asterisk (*). All V5 and TAP-tagged proteins are tagged at the N terminus.

Plasmid	Primers 5'-3'
pcDNA4/TO TAP-018	AAA**GCGGCCGC**GATGAGTTCTAAAGGCGGATCTTC
	AAA**TCTAGA**TTAGATTTTGCCGGTGCCGC
pcDNA/TO TAP-NiV-V	AAAA**GCGGCCG**CGGATAAATTGGAACTAGTCAA
	AAAA**TCTAGA**TTAACCGCAGTGGAAGCATTCAG
pcDNA4/TO TAP-018 (1-54)	AAA**GCGGCCGC**GATGAGTTCTA
	AAA**TCTAGA**TTATTTCACGCCGCCGTTCACGC
pcDNA4/TO TAP-018 (1-48)	AAA**GCGGCCGC**GATGAGTTCTA
	AAA**TCTAGA**TTAGCCAGACTTCACTCCGCTA
pcDNA4/TO TAP-018 (1-43)	AAA**GCGGCCGC**GATGAGTTCTA
	AAA**TCTAGA**TTAGCTAGATGATCCTCCGCCCCA
pcDNA4/TO TAP-018 (1-35)	AAA**GCGGCCGC**GATGAGTTCTA
	AAA**TCTAGA**TTATCCTCCGCCGCCAGATGT
pcDNA4/TO TAP-018 (1-30)	AAA**GCGGCCGC**GATGAGTTCTA
	AAA**TCTAGA**TTATGTGTATGTCTTGCTGCCCT
pcDNA4/TO TAP-018 (1-27)	AAA**GCGGCCGC**GATGAGTTCTA
	AAA**TCTAGA**TTACTTGCTGCCCTTGTTGCTG
pcDNA4/TO TAP-018 (1-24)	AAA**GCGGCCGC**GATGAGTTCTA
	AAA**TCTAGA**TTACTTGTTGCTGCCATCGTGG
pcDNA4/TO TAP-018 (1-21)	AAA**GCGGCCGC**GATGAGTTCTA
	AAA**TCTAGA**TTAGCCATCGTGGCCGTGGAT
pcDNA4/TO TAP-018 (8-60)	AAA**GCGGCCGC**GTCTGGCGGCATGTGGAGC
	AAA**TCTAGA**TTAGATTTTGCCGGTGCCGCTT
pcDNA4/TO TAP-018 (11-60)	AAA**GCGGCCGC**GATGTGGAGCGTGTTCATCCA
	AAA**TCTAGA**TTAGATTTTGCCGGTGCCGCTT
pcDNA4/TO TAP-018 (14-60)	AAA**GCGGCCGC**GGTGTTCATCCACGGCCACG
	AAA**TCTAGA**TTAGATTTTGCCGGTGCCGCTT
pcDNA4/TO TAP-018 (17-60)	AAA**GCGGCCGC**GCACGGCCACGATGGCAG
	AAA**TCTAGA**TTAGATTTTGCCGGTGCCGCTT
pcDNA4/TO TAP-018 (22-60)	AAA**GCGGCCGC**GAGCAACAAGGGCAGCAAGAC
	AAA**TCTAGA**TTAGATTTTGCCGGTGCCGCTT
pcDNA4/TO TAP-018 (31-60)	AAA**GCGGCCGC**GTCTGGCGGCGGAGGAATGT
	AAA**TCTAGA**TTAGATTTTGCCGGTGCCGCTT
pcDNA4/TO TAP-018^{AGA}	CGCCGATGGCAGCAACAAGGG*
	CCGGCGATGAACACGCTCCACATG*
pcDNA3 HA-018	SUBCLONED FROM PCDNA4/TO TAP-018
pcDNA3 HA-018^{AGA}	CGCCGATGGCAGCAACAAGGG*
	CCGGCGATGAACACGCTCCACATG*
pcDNA3 V5-STAT1	AAAA**GCGGCCGC**GTCTCAGTGGTACGAACTTCA
	AAAA**GGGCCC**TTATACTGTGTTCATCATAC
pcDNA3 V5-STAT2	AAA**GCGGCCGC**GATGGCGCAGTGGGAAATGCT
	AAA**CTCGAG**TTAGAAGTCAGAAGGCATCAAG
pcDNA3 V5-STAT3 (human)	AAA**GCGGCCGC**GATGGCCCAATGGAATCAGCT
	AAA**CTCGAG**TTACATGGGGGAGGTAGCGC
pcDNA3 V5-STAT3 (mouse)	AAA**GCGGCCGC**GGCTCAGTGGAACCAGCTGCA
	AAA**GGGCCC**TCACATGGGGGAGGTAGCAC
pcDNA3 V5-STAT4	AAA**GCGGCCGC**GATGTCTCAGTGGAATCAAGTCC
	AAA**CTCGAG**TTATTCAGCAGAATAAGGAGACTTC
pcDNA3 V5-STAT5A	AAA**GCGGCCGC**GATGGCGGGCTGGATCCA
	AAA**CTCGAG**TTATGAGAGGGAGCCTCTGGC
pcDNA3 V5-STAT5B	AAA**GCGGCCGC**GATGGCTGTGTGGATACAAGCTCA
	AAA**CTCGAG**TTACGATTGTGCGTGCGGGA
pcDNA3 V5-STAT6	AAA**GCGGCCGC**GATGTCTCTGTGGGGTCTGGTC
	AAA**CTCGAG**TTACCAACTGGGGTTGGCCCT
pcDNA3 V5-STAT1 (1-712)	AAAA**GCGGCCGC**GTCTCAGTGGTACGAACTTCA
	AAAA**GGGCCC**TTAAACTTCAGACACAGAAATCAA
pcDNA3 V5-STAT1 (1-684)	AAAA**GCGGCCGC**GTCTCAGTGGTACGAACTTCA
	AAAA**GGGCCC**TTATGGCCTGGAGTAATACTTTC
pcDNA3 V5-Fus1	AAAA**GCGGCCGC**GGCTCAGTGGAACCAGCTGCA
	*CCCTTCATTCCAAAG*GGCCAAG
	*CTTTGGAATGAAGGG*TGCATCATGGGCTTCAT
	AAAA**GGGCCC**TTATACTGTGTTCATCATAC
pcDNA3 V5-Fus2	AAAA**GCGGCCGC**GTCTCAGTGGTACGAAC
	CCCATCATTCCAGAGAGGGA
	*TCCCTCTCTGGAATGATGGG*TACATCATGGGTTTCATCAG
	AAAA**GGGCCC**TTACATGGGGGAGGTAGCACAC
pcDNA3 V5-STAT3^{Q635H} (Human)	GACCCAGATCCACTCCGTGGAAC*
	TTACCGCTGATGTCCTTC*
pF3A TAP-018	AAA**CGATCG**ATGTGGTCTCATCCTCAGTT
	AAA**GTTTAAAC**TTAGATTTTGCCGGTGCCGC
pF3A STAT1	AAA**CGATCG**ATGTCTCAGTGGTACGAACTTCA
	AAA**GTTTAAAC**TTATACTGTGTTCATCATAC
pUC13-Ecogpt-EGFP Δ018	AAA**CTGCAG**TGCCACTGTCTAGTAGTAGT
	AAA*GCGGCCGCATACTCGAG*ACACTAAATTATTTTTATTA
	AAA*CTCGAGTATGCGGCCGC*ATTAATAAGACTTAAGGACA
	AAA**GGATCC**CTAATAAGTTAATAGAACAA
pUC13-Ecogpt-EGFP TAP-018	AAA**TCTAGA**TGCCACTGTCTAGTAGTAGT.
	AAA**GCGGCCGC**GATGTCTTCTAAAGGGGGTA
	ATTAATAAGACTTAAGGACA.
	AAA**GGATCC**GCTAATAAGTTAATAGAACAA.
	AAA**CTGCAG**TGCCACTGTCTAGTAGTAGT.
	*TGTCCTTAAGTCTTATTAAT*ATGTGGTCTCATCCTCAGTT
pOPTH_TEV	CCGGAGAGCTCCAATTGGA.
	AAA**GGATCC**CTGGAAGTACAGGTTTTCGGTACCGCTATGGTGATGG TGATGATGAGCCAT
pOPTH_TEV STAT1$^{132-684,\Delta183-190,H182A,E393A,E394A}$	AAA**GGATCC**ACAGTGATGTTAGACAAACAG
	AAA**ACGCGT**TTATGGCCTGGAGTAATACTTT
	*TTGCAGAACAGAGAA*GCGGATCAGAAACAAGAACAGCTGTTACTC
	*TTCTCTGTTCTGCAA*GGTTTTG
	GATGAACATGGCGGCGTCCACCAATG*
	ACTTTTGTGTGCGTGCCC*
pEXP-nHis-STAT1-cStrep	<u>GAAAACCTGTACTTCCAG</u>GGTTCTCAGTGGTACGAACTTCAGCAG
	<u>GCTCCATGCGCTCGA</u>TACTGTGTTCATCATACTGTCGAATTC
pPEPT 018	GGAA**GGATCC**GGCGGCATGAGTTCTAAAGGCGGATC
	AAA**CTCGAG**CCGATTTTGCCGGTGCCGCTTTTC
pPEPT 018^{T2}	GGAA**GGATCC**GGCGGCATGTGGAGCGT
	AAA**CTCGAG**CCAGATGTGTATGTCTTGCTGCCC
pPEPT 018^{T3}	GGAA**GGATCC**GGCGGCATGTGGAGCGT
	AAA**CTCGAG**CCCTTGCTGCCCTTGTTGCTG
pPEPT 018^{AGA}	CGCCGATGGCAGCAACAAGGG*
	CCGGCGATGAACACGCTCCACATG*
pPEPT-NiV-V (110-140)	AAA**GGATCC**GGCGGCGTGGTTACAGACGTTGTATA
	AAA**CTCGAG**CCTGTGTAATCACTCCACCCTC
pPEPT-NiV-V (110-140)^{ADA}	TGCTGGAGGAGAATGTACCGGA*
	TCAGCGTATACAACGTCTGTAACCAC*

Table S2. Oligonucleotide primers for RT-qPCR. Related to STAR methods.

Target Gene	Primers 5’-3’
HRPT	CGAGATGTGATGAAGGAGATGG
	TTGATGTAATCCAGCAGGTCAG
IRF1	CATTCACACAGGCCGATACA
	TGGTCTTTCACCTCCTCGATA

Table S3. Oligonucleotide primers for analytical PCR or sequencing. Related to STAR methods.

Target	Primers 5-3’
STAT1 Internal	ATGCTTGCTTGGATCAGC
STAT2 Internal	GTTGGAACAGCTGGAGAC
STAT3 (human) Internal	CAACTTCAGACCCGTCAAC
STAT4 Internal	GAGCTGCAAGACTGGAA
STAT5A Internal	GATGACGAGCTGATCCAGTG
STAT5B Internal	GACGAGCTGATCCAGTGG
STAT6 Internal	GCTGGATGAAGTCCTGAG
VACWR018 Upstream	GTGAACTCTATACACCCACAC
VACWR018 Downstream	ACATTTGATTTTCTCGTACGC

Table S4. X-ray crystallographic data collection and refinement statistics. Related to Figure 7 and STAR methods.

PDB ID: 7nuf

**Data collection and processing**	
Wavelength (Å)	0.9795
Space group	C 1 2 1
Data collection temperature (K)	100
a, b, c (Å)	169.06, 37.47, 115.48
α, β, γ (°)	90.00, 116.19, 90.00
Resolution range (high resolution bin) (Å)	80.42 - 2.00 (2.00 - 2.04)
R_{meas}	0.124 (3.251)
Completeness (%)	99.7 (99.8)
Number of total / unique reflections	183215 / 44523
Redundancy	4.1 (4.3)
$\langle I/\sigma(I)\rangle$	6.6 (0.5)
$CC_{1/2}$	1.0 (0.4)
**Refinement**	
R_{cryst} / R_{free}	0.224 / 0.256
Number of reflections in test set	2136
Number of atoms	4659
Mean/Wilson B-factor ($Å^2$)	61 / 44.3
Ramachandran favoured/allowed/outliers (%)	98.86/ 1.14 / 0
RMSD bonds (Å)	0.009
RMSD angles (°)	1.309

Table S5. ITC experimental conditions and fitted parameters. Related to Figures 3, 5, 6, S6 and STAR methods.

Reaction	Cell	Syringe	K_d, nM	N	ΔH, cal / mol	ΔS, cal / mol / deg
018 + STAT1	10 µM STAT1	100 µM 018	291	1.02	-1.93×10^4	-34.9
018T2 + STAT1	15 µM STAT1	150 µM 018T2	235	1.01	-1.87×10^4	-32.3
018T3 + STAT1	15 µM STAT1	350 µM 018T3	$>10^4$	1.00 (fixed)	-2.01×10^4	-46.8
018AGA + STAT1	10 µM STAT1	100 µM 018AGA	No binding	n.a.	n.a.	n.a.
IFNGR1 + STAT1	10 µM STAT1	300 µM IFNGR1 5-mer	7.6×10^3	0.708	-9172	-7.33
IFNGR1 + STAT1 / 018	10 µM STAT1 + 50 µM 018	300 µM IFNGR1 5-mer	No binding	n.a.	n.a.	n.a.
IFNGR1 + STAT1 / 018 AGA	10 µM STAT1 + 50 µM 018 AGA	300 µM IFNGR1 5-mer	7.8×10^3	0.79	-8001	-3.48
IFNGR1 + STAT1 / NiV-V	10 µM STAT1 + 200 µM NiV- V	300 µM IFNGR1 5-mer	No binding	n.a.	n.a.	n.a.
IFNGR1 + STAT1 / NiV-V ADA	10 µM STAT1 + 200 µM NiV-V ADA	300 µM IFNGR1 5-mer	7.2×10^3	0.88	-8125	-3.72
018 21-mer + STAT1	10 µM STAT1	100 µM 018 21-mer	321	1.09	-1.59×10^4	-23.9
p018 21-mer + STAT1	10 µM STAT1	100 µM p018 21-mer	174	1.12	-9139	0.276

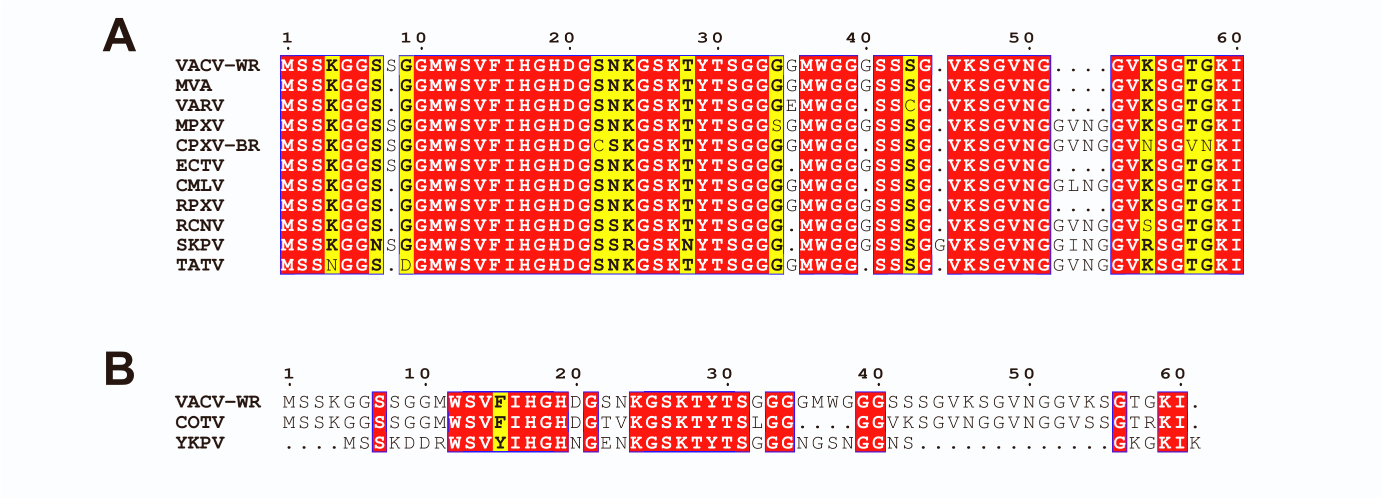

Figure S1. Sequence alignment of 018 orthologues. Related to Figure 1. (**A**) Alignment of 018 orthologues from representative orthopoxviruses: vaccinia virus strain Western Reserve (VACV-WR), modified vaccinia Ankara (MVA), variola virus (VARV), monkeypox virus (MPXV), cowpox virus strain Brighton Red (CPXV), ectromelia virus (ECTV), camelpox virus (CMPV), rabbitpox virus (RPXV), racconpox virus (RCNV), skunxpox virus (SKPV) and taterpox virus (TATV). The 018 ORF is absent from VACV strain Copenhagen. (**B**) Alignment of 018 poxvirus orthologues from Cotia virus (COTV) and yokapoxvirus (YKPV), which sit outside of the orthopoxvirus genus. Identical residues are shown in red, similar residues are shown in yellow (**A-B**).

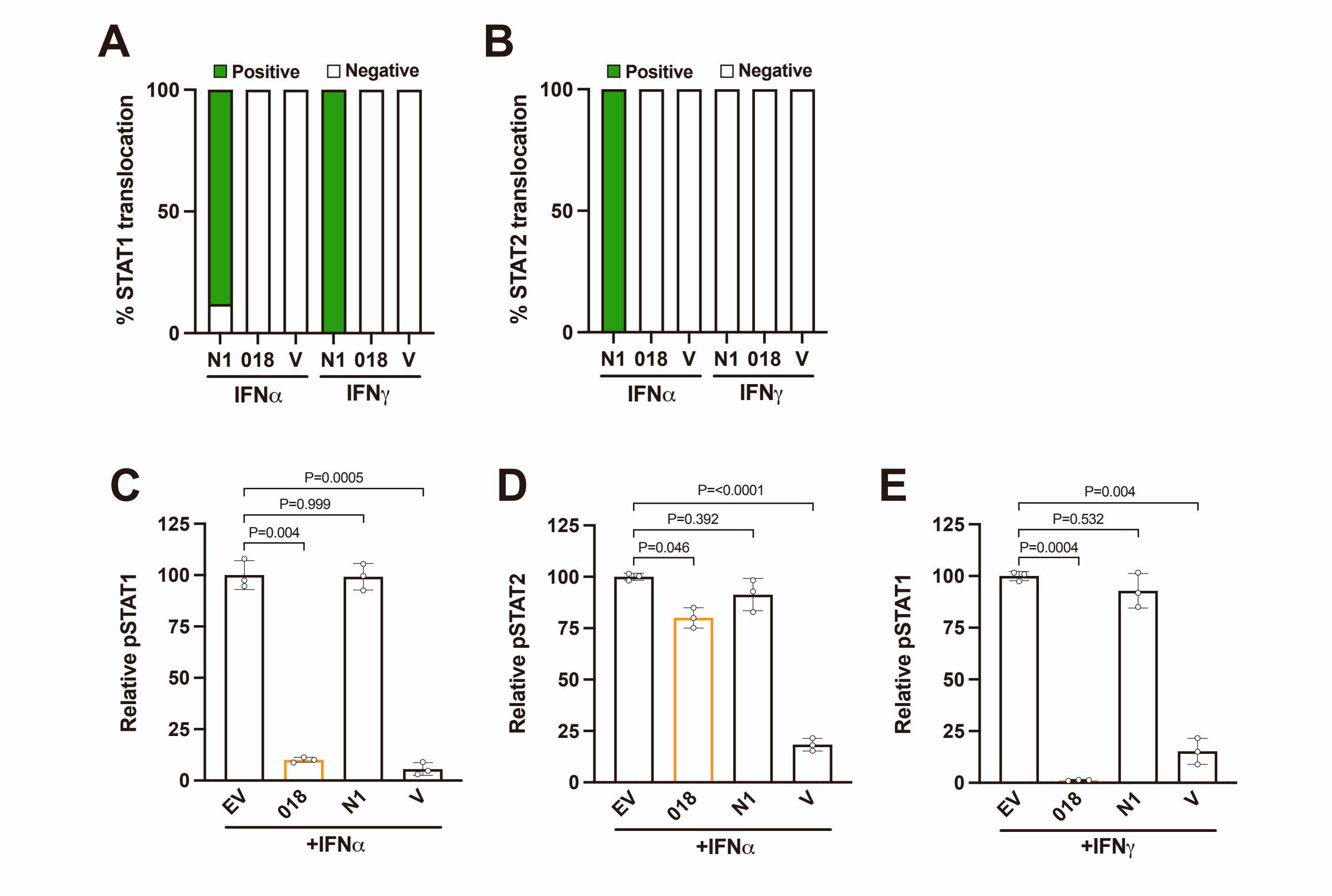

Figure S2. Quantification of STAT1 and STAT2 phosphorylation and translocation into the nucleus. Related to Figure 2. (**A-B**) Percentage of cells expressing TAP-tagged N1, 018 and NiV-V (V) that show nuclear STAT1 (**A**) and STAT2 (**B**) following stimulation with IFNα or IFNγ from (**Figure 2A, B**), (n=25 cells per condition). Cells positive for translocation are shown in green. (**C-E**) Quantification of relative band intensities for pSTAT1 (**C**) and pSTAT2 (**D**) from (**Figure 2C**) and pSTAT1 (**E**) from (**Figure 2D**). pSTAT levels were normalised against total STAT levels and made relative to EV condition. Means ± SD (n=3 per condition) are shown. Significances were calculated by Dunnett's T3 multiple comparisons test.

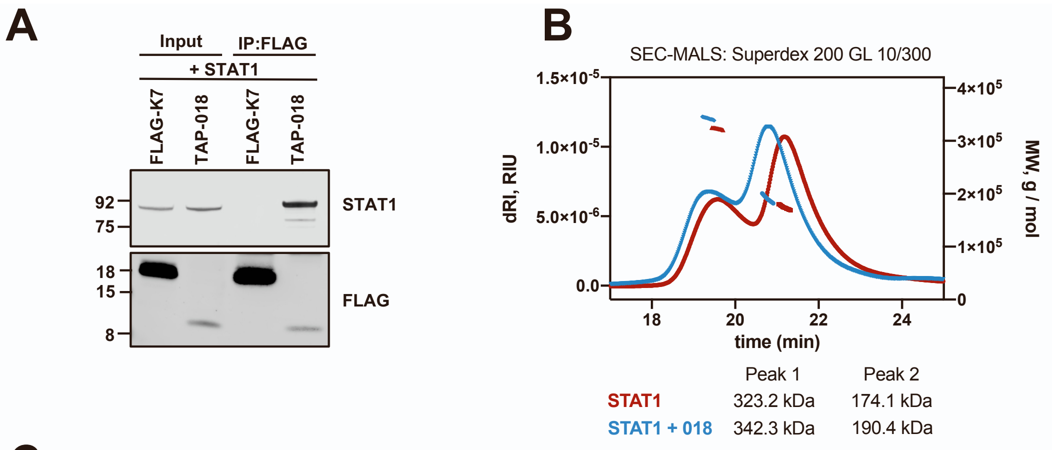

C

```
018 FL MSSKGGSSGGMWSVFIHGHDGSNKGSKTYTSGGGGMWGGGSSSGVKSGVNGGVKSGTGKI
1-30   MSSKGGSSGGMWSVFIHGHDGSNKGSKTYT------------------------------
1-27   MSSKGGSSGGMWSVFIHGHDGSNKGSK---------------------------------
1-24   MSSKGGSSGGMWSVFIHGHDGSNK------------------------------------
1-21   MSSKGGSSGGMWSVFIHGHDG---------------------------------------
11-60  ----------MWSVFIHGHDGSNKGSKTYTSGGGGMWGGGSSSGVKSGVNGGVKSGTGKI
14-60  -------------VFIHGHDGSNKGSKTYTSGGGGMWGGGSSSGVKSGVNGGVKSGTGKI
17-60  ----------------HGHDGSNKGSKTYTSGGGGMWGGGSSSGVKSGVNGGVKSGTGKI
```

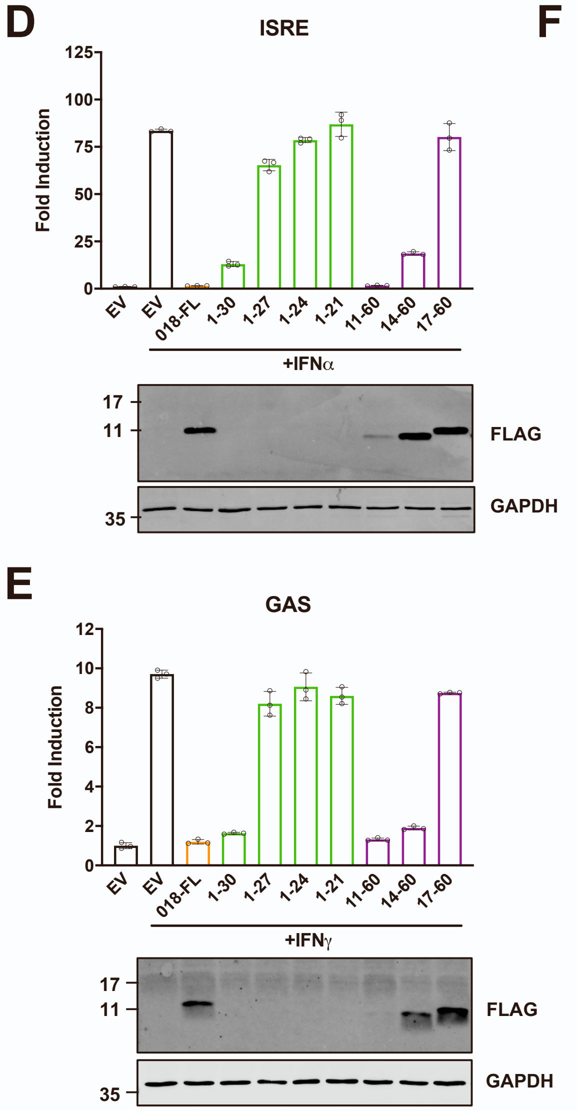

F

Construct	% Inhibition		Relative expression level	
	ISRE	GAS	ISRE	GAS
018-FL	>95%	>95%	WT	WT
1-54	>95%	>95%	WT	Low
1-48	>95%	>95%	WT	Very Low
1-43	>95%	>95%	Low	Undetectable
1-35	>95%	>95%	Very Low	Undetectable
1-30	>75%	>75%	Undetectable	Undetectable
1-27	<25%	<25%	Undetectable	Undetectable
1-24	<25%	<25%	Undetectable	Undetectable
1-21	<25%	<25%	Undetectable	Undetectable
8-60	>95%	>95%	WT	Low
11-60	>95%	>95%	Low	Very Low
14-60	>75%	>75%	WT	WT
17-60	<25%	<25%	WT	WT
22-60	<25%	<25%	WT	WT
31-60	<25%	<25%	Low	Very low

Figure S3. 018 binds STAT1 without affecting its oligomeric state and inhibits IFN-signalling via a 21 aa sequence. Related to Figure 3. (**A**) 018:STAT1 interaction using a cell-free transcriptional and translation system. Untagged-STAT1 was co-expressed along with either FLAG-tagged K7 or TAP-tagged 018 using a wheat germ cell free transcriptional and translation system. FLAG-K7 and TAP-018 were

precipitated using M2-FLAG affinity gel and purified proteins were analysed by immunoblotting with α-FLAG and α-STAT1 antibodies. (**B**) SEC-MALS measurements of free STAT1 (red) and STAT1:GB1-018 complex (blue). One hundred μl samples were loaded on a Superdex 200 GL 10/300 column and scattering and refractive index of the eluting peaks were measured. Concentration of 20 μM for STAT1 and 100 μM of GB1-018 were applied. (**C**) Sequences for C-terminal (green) and N-terminal (purple) 018 refined truncation mutants. (**D**) HEK 293T cells or (**E**) HeLa cells were transfected with reporter plasmids ISRE-Luc (**D**) or GAS-Luc (**E**) plus TK-*Renilla* and vectors expressing 018 truncation mutants from (**C**) fused to a TAP-tag. Cells were stimulated with IFNα (1000 U/mL) (**D**), or IFNγ (25 ng/mL) (**E**) for 6 h (**D**) or 8 h (**E**) and luciferase values were measured. Means ± SD (n=3 per condition) are shown. Lysates were prepared and analysed by immunoblotting with α-FLAG and α-GAPDH. (F) Summary of all C-terminal (green) and N-terminal (purple) 018 truncation mutants describing the percentage inhibitory activity (>95%, >75% (but less then >95%) or <25%) and relative protein expression levels (WT (wild-type)), low, very low or undetectable) for ISRE (IFNα) and GAS (IFNγ) reporters. Data from (**D-E**) are representative of 2 individual experiments.

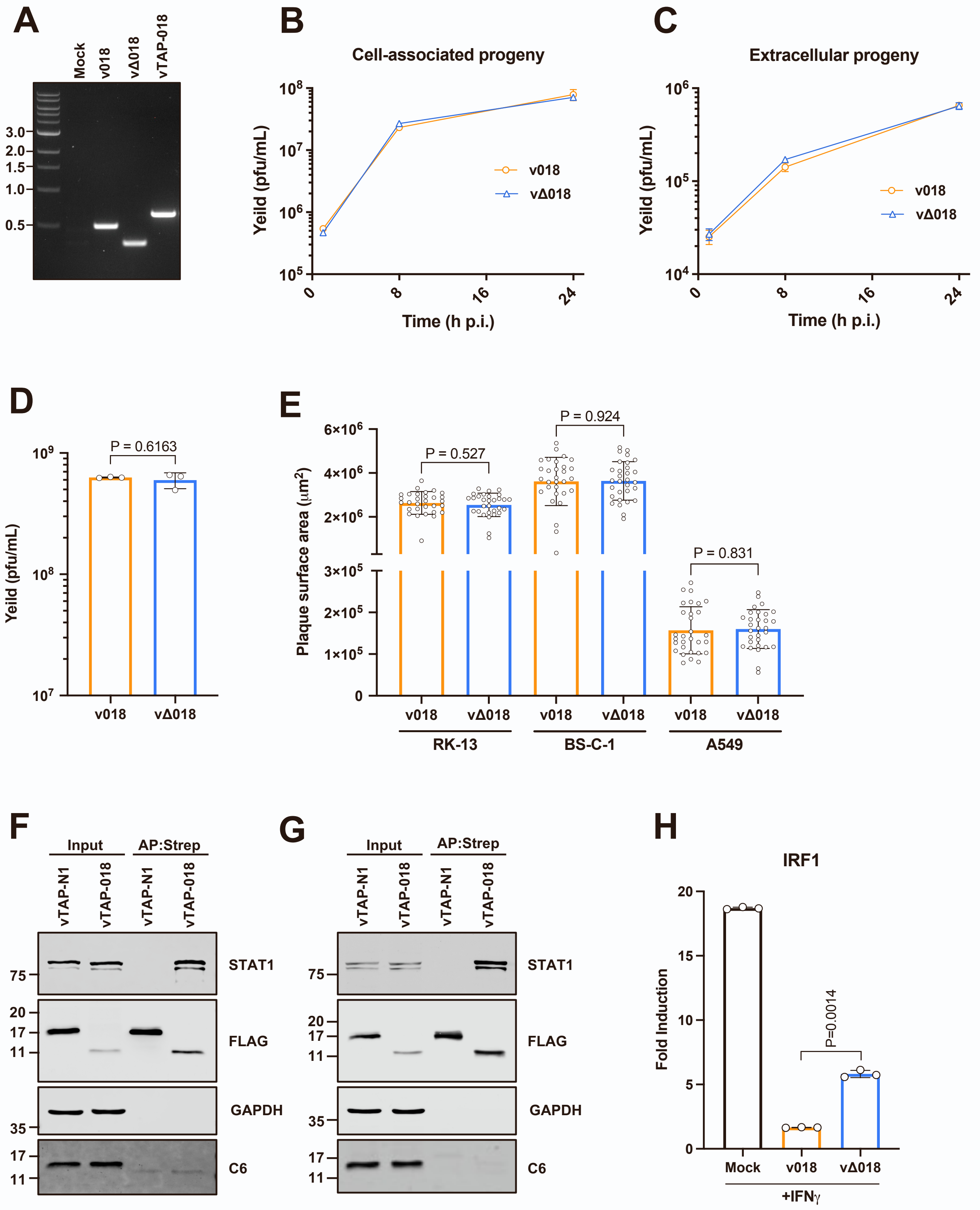

Figure S4. Characterisation of VACV-WR deletion mutant lacking gene *VACWR018*. Related to Figure 4. (**A**) PCR amplification of genomic DNA from the indicated viruses using primers upstream and downstream of the 018 ORF. The position of DNA size markers (kbp) are shown on the left side of the image. (**B, C**) BS-C-1 cells were infected with either v018 (orange) or vΔ018 (blue) at 5 pfu/cell. At 1, 8 and 24 h p.i. infectious virus titres associated with cells (**B**) and in the supernatants (**C**) was determined by plaque assay on BS-C-1 cells. Means ± SD (n=2 per condition) are shown and P=>0.05 for all timepoints. (**D**) A549 cells were infected with either v018 (orange) or vΔ018 (blue) at 5 pfu/cell. At 24 h p.i., cells and supernatant were collected, and infectious virus titres were determined by plaque assay on BS-C-1 cells. **(E)** RK13, BS-C-1 and A549 cells were infected at 30 pfu per well with either v018 (orange) or vΔ018

(blue). At 72 h p.i. monolayers were stained and plaque surface areas were quantified. Means ± SD (n=30 plaques per condition) are shown. (**F**) BS-C-1 cells or (**G**) MEFs were infected with vTAP-018 or vTAP-N1 at 5 pfu/cell for 12 h. TAP-tagged proteins were affinity-purified by Strep-Tactin and whole cell lysates (Input) and affinity-purified proteins (AP:Strep) were analysed by immunoblotting with α-FLAG, α-GAPDH, α-STAT1 and α-C6. (**H**) A549 cells were mock-infected or infected with v018 or vΔ018 at 10 pfu/cell. At 2 h p.i. cells were stimulated IFNγ (25 ng/mL) for 1 h. Total RNA was extracted and mRNA for IRF1 was analysed by RT-qPCR. Means ± SD (n=3 per condition) are shown. Data from (**B-H**) representative of 2 individual repeats. Significances were determined using Unpaired t-test with Welch's correction (**B-E**, **H**).

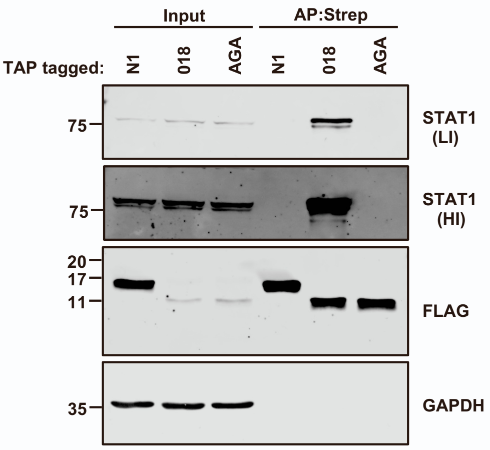

Figure S5. The 018 HxH motif is essential for 018 co-precipitation with STAT1 from cells. Related to Figure 6. TAP-tagged 018, 018 AGA (labeled AGA) and N1 were expressed by transfection in 2fTGH cells and affinity purified by Strep-Tactin. Whole cell lysates (Input) and affinity-purified proteins (AP:Strep) were analysed by immunoblotting with α-FLAG, α-GAPDH and α-STAT1. A high intensity (HI) and low intensity (LI) scan of α-STAT1 are shown. Data is representative of 2 independent repeats.

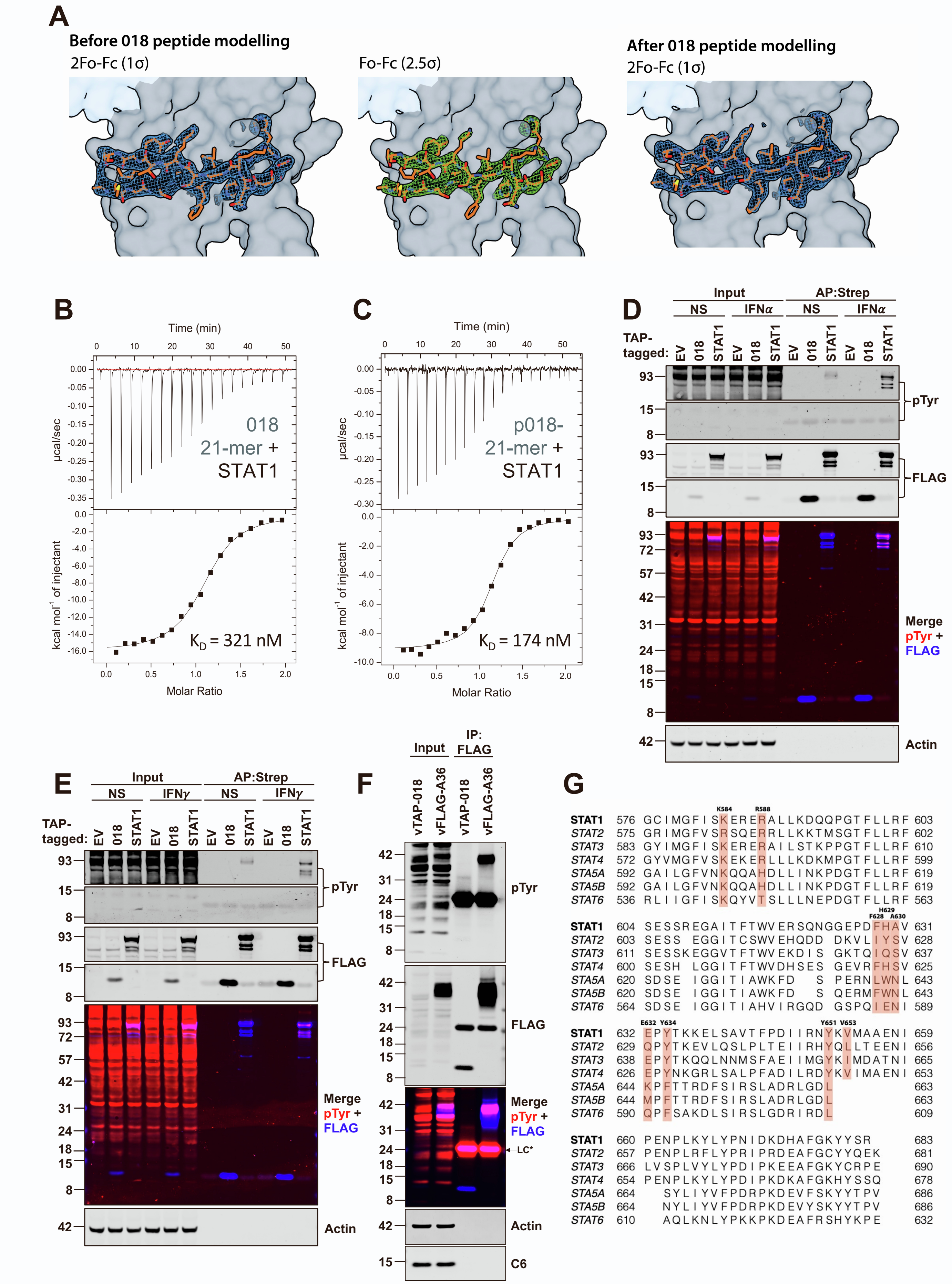

Figure S6. Analysis of the 018 pTyr-independent mode of STAT binding. Related to Figure 7. (**A**) Electron density maps of 018 peptide before and after peptide modelling and refinement. (**B-C**) ITC data for 100 µM 018 21-mer (**B**) and 100 µM p018 21-mer (pTyr29) (**C**) titrated into 10 µM U-STAT1. Fitting of the isotherm (bottom) to a one site model gave a K_D of 321 nM (**B**) and 174 nM (**C**). (**D-E**) EV,

TAP-tagged 018 and TAP-tagged STAT1 were expressed by transfection in HEK 293T cells and at 16 h post-transfection, cells were kept either non-stimulated (NS) or were stimulated with IFNα (1000 U/mL) (**D**), or IFNγ (25 ng/mL) (**E**) for 30 min and TAP-tagged proteins were affinity-purified by Strep-Tactin. Whole cell lysates (Input) and affinity-purified proteins (AP:Strep) were analysed by immunoblotting with α-pTyr, α-FLAG and α-actin. (**F**) A549 cells were infected with either vTAP-018 or vFLAG-A36 (a VACV protein phosphorylated on Tyr), at 5 pfu/cell and at 12 h p.i., TAP-tagged and FLAG-tagged proteins were immunoprecipitated using M2-FLAG affinity gel. Whole cell lysates (Input) and immunoprecipitated proteins (IP:FLAG) were analysed by immunoblotting with α-pTyr, α-FLAG, α-actin and α-C6. LC = IgG light chain. Data from (**D-F**) are representative of 2 individual repeats. (**G**) Alignment of STAT family SH2 domains with residues that form contacts with 018 in the 018:STAT1 crystal structure highlighted in orange.

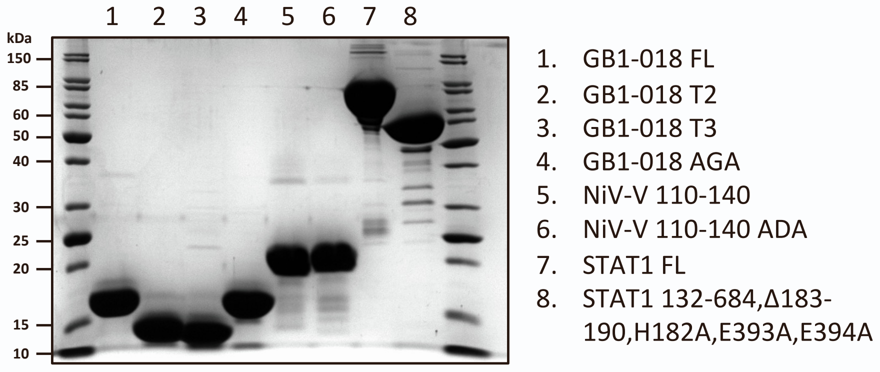

Figure S7. Coomassie-stained SDS-polyacrylamide gel of purified recombinant proteins. Related to STAR methods.
