# Supplementary material for: Poxviruses and paramyxoviruses use a conserved mechanism of STAT1 antagonism to inhibit interferon signaling
Source: Cell Host Microbe. 2022 Mar 9;30(3):357–372.e11. doi: 10.1016/j.chom.2022.01.014 (PMC8912257; doi:10.1016/j.chom.2022.01.014)
Supplement: Document S2. Article plus supplemental information [file mmc3.pdf]

Article

Cell Host & Microbe

Poxviruses and paramyxoviruses use a conserved mechanism of STAT1 antagonism to inhibit interferon signaling

Graphical abstract

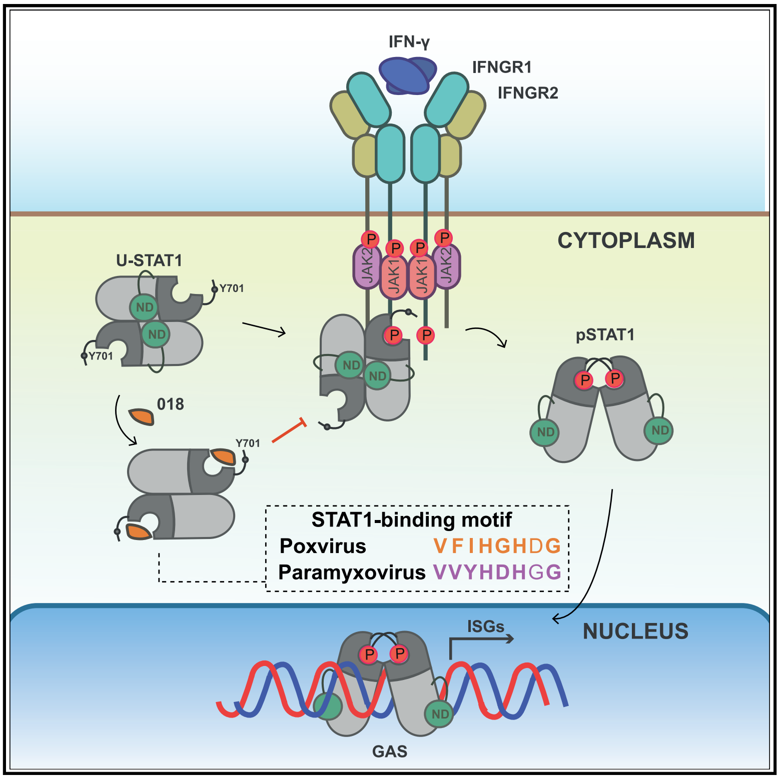

Authors

Callum Talbot-Cooper, Teodors Pantelejevs, John P. Shannon, ..., Marko Hyvönen, Heather D. Hickman, Geoffrey L. Smith

Correspondence

gls37@cam.ac.uk

In brief

Examining the poxvirus protein 018, Talbot-Cooper et al. uncover a conserved viral strategy to inhibit host anti-viral responses. 018 binds the STAT1 SH2 domain to block its recruitment to IFN receptors. The STAT1-binding motif of 018 is present in diverse virus families, highlighting its convergent evolution.

Highlights

- Poxvirus protein 018 is a virulence factor that inhibits IFN-induced signaling
- 018 binds the STAT1 SH2 domain to block its recruitment to the IFNγ receptor
- The structure of the 018:STAT1 complex reveals a pTyr-independent binding mode
- Vaccinia and Nipah viruses acquired a STAT1-binding motif by convergent evolution

Talbot-Cooper et al., 2022, Cell Host & Microbe *30*, 357–372
March 9, 2022 © 2022 The Authors. Published by Elsevier Inc.
https://doi.org/10.1016/j.chom.2022.01.014

CellPress

Cell Host & Microbe

CellPress
OPEN ACCESS

Article

Poxviruses and paramyxoviruses use a conserved mechanism of STAT1 antagonism to inhibit interferon signaling

Callum Talbot-Cooper,[1] Teodors Pantelejevs,[2,4] John P. Shannon,[1,3] Christian R. Cherry,[3] Marcus T. Au,[1] Marko Hyvönen,[2] Heather D. Hickman,[3] and Geoffrey L. Smith[1,5,*]

[1]Department of Pathology, University of Cambridge, Tennis Court Road, Cambridge CB2 1QP, UK
[2]Department of Biochemistry, University of Cambridge, 80 Tennis Court Road, Cambridge CB2 1GA, UK
[3]Viral Immunity and Pathogenesis Unit, Laboratory of Clinical Immunology and Microbiology, NIAD, NIH, Bethesda, MD 20852, USA
[4]Latvian Institute of Organic Synthesis, Aizkraukles 21, LV-1006 Riga, Latvia
[5]Lead contact
*Correspondence: gls37@cam.ac.uk
https://doi.org/10.1016/j.chom.2022.01.014

SUMMARY

The induction of interferon (IFN)-stimulated genes by STATs is a critical host defense mechanism against virus infection. Here, we report that a highly expressed poxvirus protein, 018, inhibits IFN-induced signaling by binding to the SH2 domain of STAT1, thereby preventing the association of STAT1 with an activated IFN receptor. Despite encoding other inhibitors of IFN-induced signaling, a poxvirus mutant lacking 018 was attenuated in mice. The 2.0 Å crystal structure of the 018:STAT1 complex reveals a phosphotyrosine-independent mode of 018 binding to the SH2 domain of STAT1. Moreover, the STAT1-binding motif of 018 shows similarity to the STAT1-binding proteins from Nipah virus, which, similar to 018, block the association of STAT1 with an IFN receptor. Overall, these results uncover a conserved mechanism of STAT1 antagonism that is employed independently by distinct virus families.

INTRODUCTION

Interferons (IFNs) induce signal transduction to upregulate IFN-stimulated genes (ISGs) that inhibit virus replication (Schneider et al., 2014). Signal transduction is mediated by signal transducers of transcription (STAT) proteins STAT1 and STAT2, which, when unphosphorylated, form latent hetero (U-STAT1-U-STAT2) or homodimers (U-STAT1) (Mao et al., 2005; Wang et al., 2021). IFNs bind their cognate receptors to activate receptor-associated kinases that phosphorylate receptor tails, creating a docking site for STAT SH2 domains. At receptors, STATs are phosphorylated (pSTAT) and undergo dimer rearrangement from an anti-parallel to a parallel conformation, mediated by a reciprocal pTyr:SH2 interaction between two pSTATs (Wenta et al., 2008).

Type I IFNs (IFN-I) signal via the IFNα/β receptor (IFNAR) to activate kinases that phosphorylate STAT1 and 2. The pSTAT1:-STAT2 heterodimer associates with IRF9 to form the IFN-stimulated gene factor 3 (ISGF3) complex (Rengachari et al., 2018). Type II IFN (IFN-II or IFNγ) signals via the IFNγ receptor (IFNGR) and activates kinases that phosphorylate STAT1 only. The pSTAT1 homodimer is called the γ-activated factor (GAF). ISGF3 and GAF drive the transcription of ISGs with IFN-stimulated responsive element (ISRE) or γ-activated sequence (GAS) promoters, respectively (Aaronson and Horvath, 2002).

To overcome the anti-viral activities of IFNs, viruses have evolved many strategies to antagonize host IFN pathways (for review, see García-Sastre, 2017; Randall and Goodbourn, 2008). Given the importance of viral-mediated IFN-signaling antagonism for virus replication, insight into these strategies can guide novel anti-viral therapeutic approaches.

Poxviruses are large, cytoplasmic DNA viruses. Vaccinia virus (VACV) is the prototypic poxvirus, the vaccine used to eradicate smallpox and an excellent model to study host-pathogen interactions. VACV encodes ~200 proteins, of which >1/3 modulate host immune responses, including proteins that target IFN-induced signaling (Smith et al., 2013, 2018). VACV proteins B18 and B8 act as soluble IFN receptors that bind IFN-I and IFN-II, respectively (Alcamí and Smith, 1995; Colamonici et al., 1995; Mossman et al., 1995; Symons et al., 1995). At the intracellular level, the viral phosphatase vH1 dephosphorylates STAT1 (Koksal et al., 2009; Najarro et al., 2001), and protein C6 inhibits IFN-I signaling in the nucleus (Stuart et al., 2016).

Here, we show that an uncharacterized VACV protein (018), encoded by VACV strain Western Reserve (WR) gene *VACWR018*, binds directly to the SH2 domain of STAT1 and competes with a phosphorylated IFN receptor to prevent STAT1-receptor association and therefore STAT1 phosphorylation. A VACV lacking 018 was attenuated in mice and induced

© 2022 The Authors. Published by Elsevier Inc.
This is an open access article under the CC BY license (http://creativecommons.org/licenses/by/4.0/).

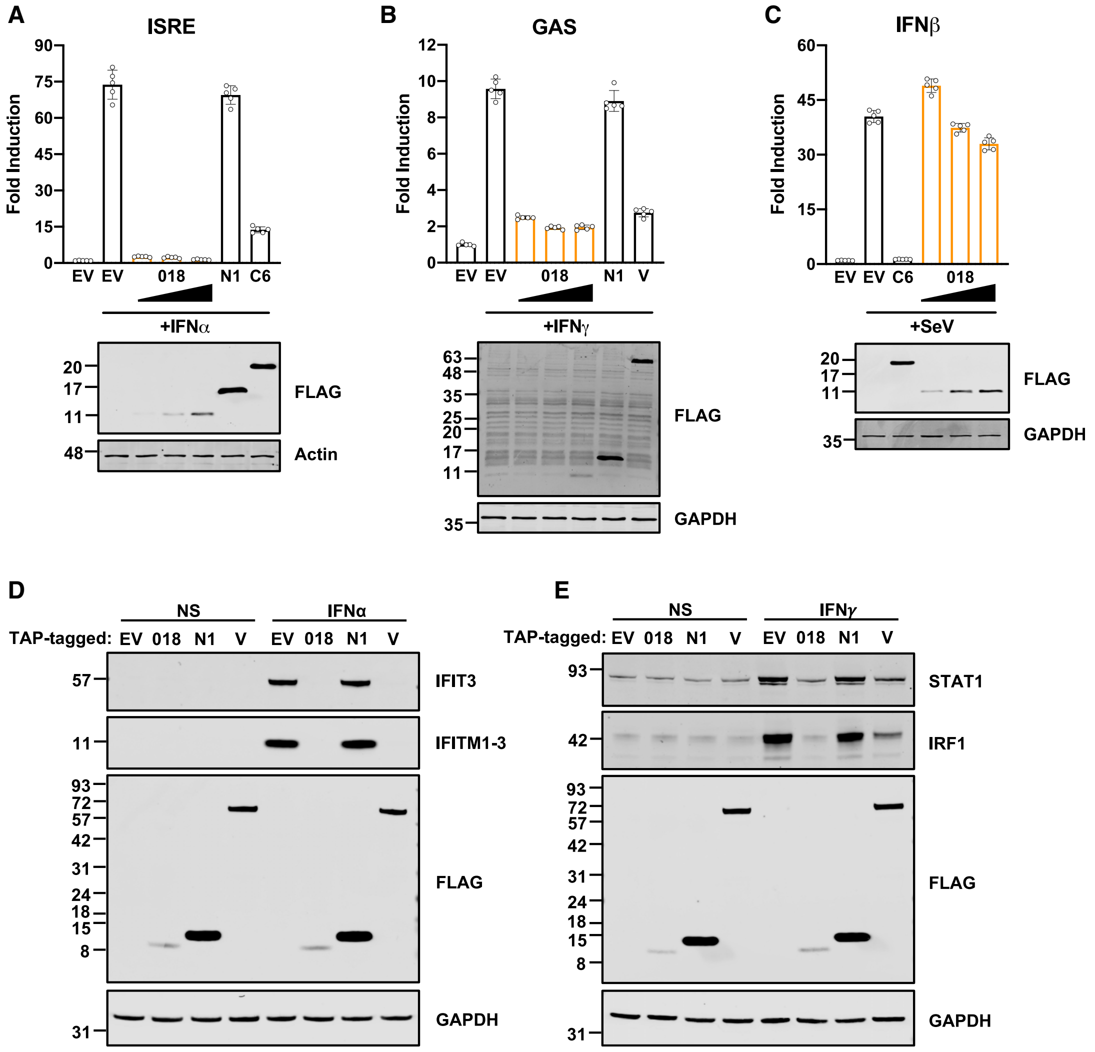

Figure 1. VACV protein 018 inhibits IFN-induced signaling

(A–C) (A and C) HEK 293T or (B) HeLa cells were transfected with reporter plasmids ISRE-Luc (A), GAS-Luc (B), or IFNβ-Luc (C), plus *TK-Renilla* and empty vector (EV) or vectors expressing indicated proteins fused to a TAP tag. Cells were stimulated with IFNα (A), IFNγ (B), or SeV (C), for 6 (A), 8 (B), or 24 h (C) and then luciferase activity was measured, and lysates were analyzed by immunoblotting. Means ± SD (n = 5 per condition) are shown.

(D and E) T-REx 293 cells expressing indicated proteins were stimulated with IFNα (D) or IFNγ (E) for 24 h and lysates were analyzed by immunoblotting. Data for (A–C) and (D and E) are representative of 3 or 2 individual experiments, respectively.

enhanced innate immune signaling, demonstrating the *in vivo* importance of this inhibitor. The crystal structure of 018 complexed with STAT1 was determined to 2.0 Å. This revealed a key contact that enables 018 to bind STAT1 and STAT4 selectively, and a non-canonical SH2-binding mode, whereby 018 occupies the SH2 domain in a pTyr pocket-independent manner with high affinity. Furthermore, the STAT1-binding region of 018 shares remarkable similarity to STAT1-binding regions of V/W and P proteins from Nipah virus (NiV), a highly pathogenic paramyxovirus. Similar to 018, we show that the minimal STAT1-binding region of the NiV-V protein can compete with a phosphorylated IFN receptor to bind STAT1. This study reveals a conserved mechanism for targeting STAT1 utilized by poxviruses and paramyxoviruses to subvert cellular anti-viral responses.

RESULTS

VACWR018 is located in a genomic region encoding immunomodulators (Gubser et al., 2004), is transcribed early after infection, and is one of the most abundant viral transcripts (Assarsson et al., 2008; Wennier et al., 2013; Yang et al., 2015). Protein 018 is highly conserved within the orthopoxvirus genus, including human pathogens such as cowpox virus, monkeypox virus, and both the 20th century and Viking era variola viruses, the cause of smallpox (Mühlemann et al., 2020) (Figure S1). Given these features, we explored whether 018 modulates anti-viral immunity.

VACV protein 018 inhibits IFN-induced signaling

To test this, luciferase reporter plasmids were used that are activated by specific anti-viral signaling pathways. Activation

CellPress
OPEN ACCESS

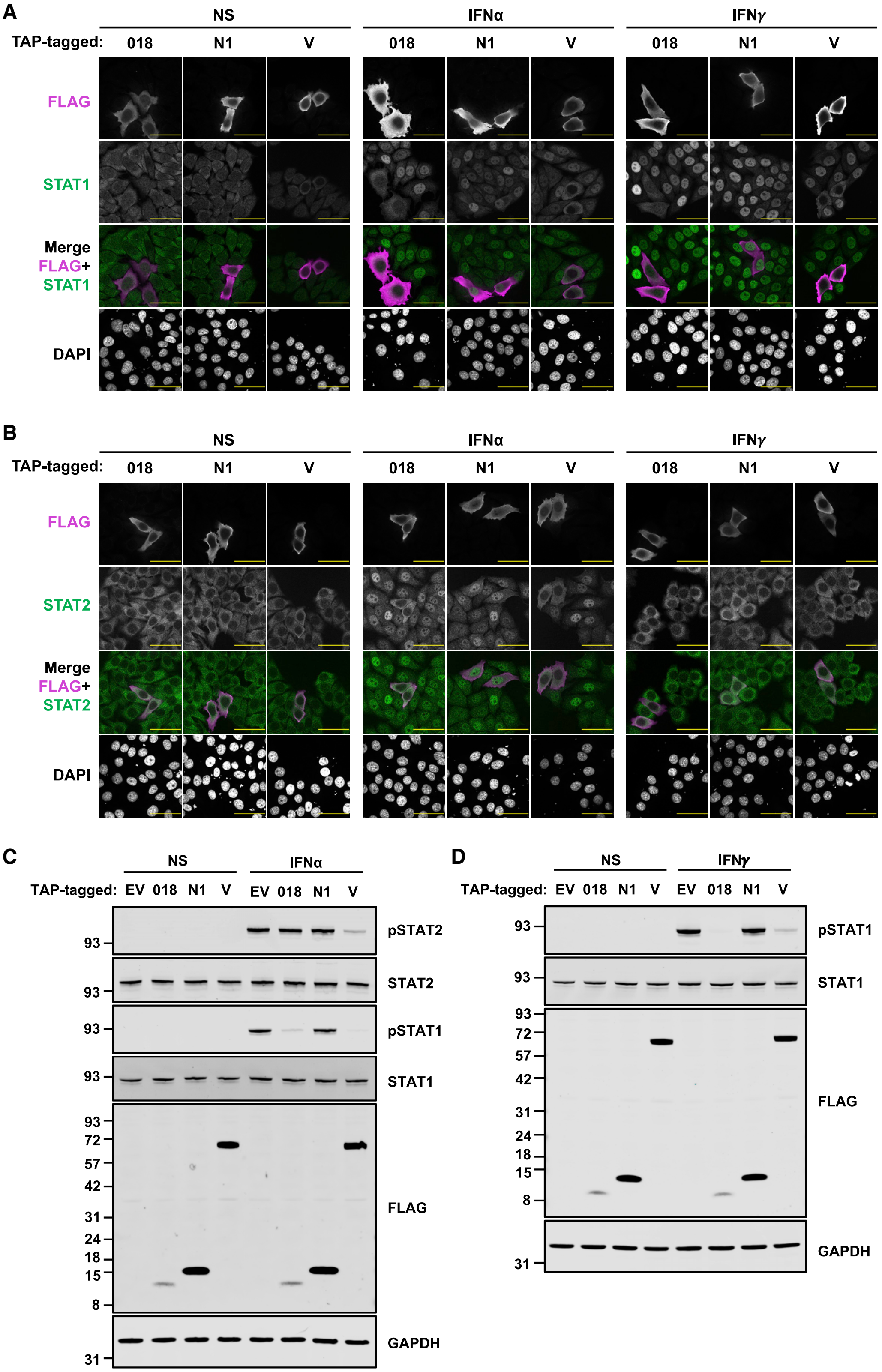

(legend on next page)

of IRF3, NF-κB, and AP-1 pathways that induce IFNβ was measured using an IFNβ-Luc reporter after stimulation with Sendai virus (SeV). IFN-induced pathways were measured using ISRE- or GAS-Luc reporters after stimulation with IFN-I or II, respectively.

Tandem affinity purifcation (TAP)-tagged (2x Strep, 1x FLAG epitope) 018 inhibited pathway activation induced by IFN-I and II (Figures 1A and 1B) but had little effect on the activation of IFN-β-Luc (Figure 1C). NiV-V (Rodriguez et al., 2002) and VACV protein C6 (Stuart et al., 2016; Unterholzner et al., 2011) served as positive controls, whereas VACV protein N1 (Maluquer de Motes et al., 2011) served as a negative control.

Next, the effect of 018 on endogenous ISGs was tested by immunoblotting for representative ISGs in T-Rex cell lines that inducibly expressed TAP-tagged 018 or controls (empty vector [EV], TAP-tagged N1, or NiV-V). Stimulation with IFN-I or -II increased the ISG levels in cells expressing EV but not those expressing 018 (Figures 1D and 1E). These data show that 018 is a potent inhibitor of IFN-I- and -II-induced signaling.

Phosphorylation of STAT1 at Tyr701 is blocked by 018

Next, the ability of 018 to inhibit STAT nuclear translocation was tested by confocal microscopy. In untransfected cells, addition of IFN-I or -II induced STAT1 redistribution to the nucleus (Figure 2A), whereas only IFN-I did so for STAT2 (Figure 2B). In contrast, 018 blocked STAT1 and 2 translocation (Figures 2A, 2B, S2A, and S2B). As reported, NiV-V also blocked STAT1 translocation, but unlike 018, NiV-V redistributed STAT1 to a predominantly cytoplasmic localization in resting cells due to its nuclear export signal (Rodriguez et al., 2004) (Figure 2A).

To test if 018 inhibited phosphorylation of STAT1 at Tyr701 (pSTAT1) and STAT2 at Tyr690 (pSTAT2), T-REx 293 cells expressing TAP-tagged 018 or controls were IFN-stimulated and analyzed by immunoblotting. IFN-I stimulation increased the pSTAT1 and pSTAT2 levels in both EV and N1-expressing cells, whereas 018 greatly reduced the pSTAT1 level (Figures 2C and S2C) but only affected the pSTAT2 level marginally (Figures 2C and S2D). As reported, NiV-V blocked STAT1 phosphorylation (Rodriguez et al., 2002) (Figures 2C and S2C). NiV-V also blocked STAT2 phosphorylation (Figures 2C and S2D), which has not been reported, but is consistent with NiV-V harboring a distinct STAT2-binding site (Rodriguez et al., 2004). IFN-II increased the pSTAT1 level in control cells, whereas STAT1 phosphorylation was blocked by 018 (and NiV-V) (Figures 2D and S2E). These data show that 018 blocks the phosphorylation of STAT1 at Tyr701 after stimulation with IFN-I or -II and thus prevents STAT1/2 translocation.

A 21 aa fragment of 018 is sufficient to bind STAT1

Next, we assessed if 018 interacts with key cellular proteins involved in IFN signal transduction by pull-down in 2fTGH cells (a human cell line containing the selectable marker guanine phosphoribosyltransferase regulated by IFNα; Pellegrini et al., 1989). 018 co-precipitated STAT1 and, to a lesser degree, STAT2 but not IRF9 (Figure 3A). In 2fTGH-derived U3A (STAT1$^{-/-}$) cells, the 018:STAT2 interaction was lost, indicating that the interaction was likely indirect via STAT1 (Figure 3B). In 2fTGH-derived U6A (STAT2$^{-/-}$) cells, the 018:STAT1 interaction was retained (Figure 3B). The 018:STAT1 interaction was shown to be direct as 018 and STAT1 co-precipitated when produced using a cell-free protein expression system (Figure S3A).

To study the 018:STAT1 interaction, each protein was expressed and purified from *E. coli*. 018 was fused to the B1 domain of protein G (GB1) to improve expression and solubility. Using isothermal titration calorimetry (ITC), we observed a K_D of 291 nM and a stoichiometry of 1.02, meaning that one 018 molecule binds per U-STAT1 protomer (Figure 3C). The effect of 018 on U-STAT1 quaternary assembly was evaluated by SEC-MALS. U-STAT1 alone eluted mostly as tetrameric and dimeric species, and preincubation with excess 018 caused the two peaks to have earlier elution volumes and increased masses (Figure S3B), indicating that 018 binds U-STAT1 without altering its oligomeric state.

Next, the region of 018 needed to inhibit IFN-I- and -II-induced signaling was mapped using C- and N-terminal 018 truncation mutants (Figure 3D). Inhibitory activity was categorized as (1) >95%, (2) between 75% and 95%, or (3) <25%, deemed to be non-inhibitory. Mutant 1–35 had the largest C-terminal truncation but still demonstrated >95% inhibition (Figures 3E and 3F). Mutant 8–60 inhibited >95%, whereas mutant 22–60 lost inhibitory activity (<25%) (Figures 3E and 3F). These data show that aa 8–35 of 018 are sufficient for pathway inhibition.

To refine the inhibitory region, additional mutants truncating inward from aa 8 and 35 were constructed (Figure S3C). Mutant 11–60 retained >95% inhibition, whereas mutants with further N-terminal truncation had reduced inhibitory activity (Figures S3D and S3E). Mutant 1–30 inhibited between 75% and 95%, demonstrating a marginal loss in inhibitory activity; however, the expression was undetectable (Figures S3D and S3E). All further C-terminal truncations showed <25% inhibitory activity, but the expression was undetectable (Figures S3D and S3E). The same pattern of inhibitory activity by 018 mutants was observed for both IFN-I and -II signaling (Figure S3F), indicating that the same region of 018 is required to inhibit both pathways.

These observations map a putative minimal inhibitory region of 018 to aa 11–31. The C-terminal boundary was defined assuming the slight reduction in inhibitory activity after the deletion of residues 35–31 was due to lower protein expression levels, whereas further truncation removed functional residues. Ser31 was included as it is highly conserved in orthopoxvirus orthologues of 018 (Figure S1).

Figure 2. Phosphorylation of STAT1 at Tyr701 is blocked by 018

(A and B) HeLa cells were transfected with plasmids expressing TAP-tagged 018, N1, or NiV-V and stimulated with IFNα or IFNγ for 1 h. Cells were immunostained with α-FLAG (pink) (A and B) and either α-STAT1 (green) (A) or α-STAT2 (green) (B) and DNA stained with DAPI. Cells were visualized by confocal microscopy. Scale bar (yellow) = 50 μm. Quantification of STAT1/2 translocation in transfected cells for (A and B) is provided in Figures S2A and S2B.

(C and D) T-REx 293 cells expressing indicated proteins were stimulated with IFNα (C) or IFNγ (D) for 30 min, and lysates were analyzed by immunoblotting. Quantification of band intensities for (C and D) is provided in Figures S2C–S2E. Data for (A and B) and (C and D) are representative of 2 or 3 individual experiments, respectively.

CellPress
OPEN ACCESS

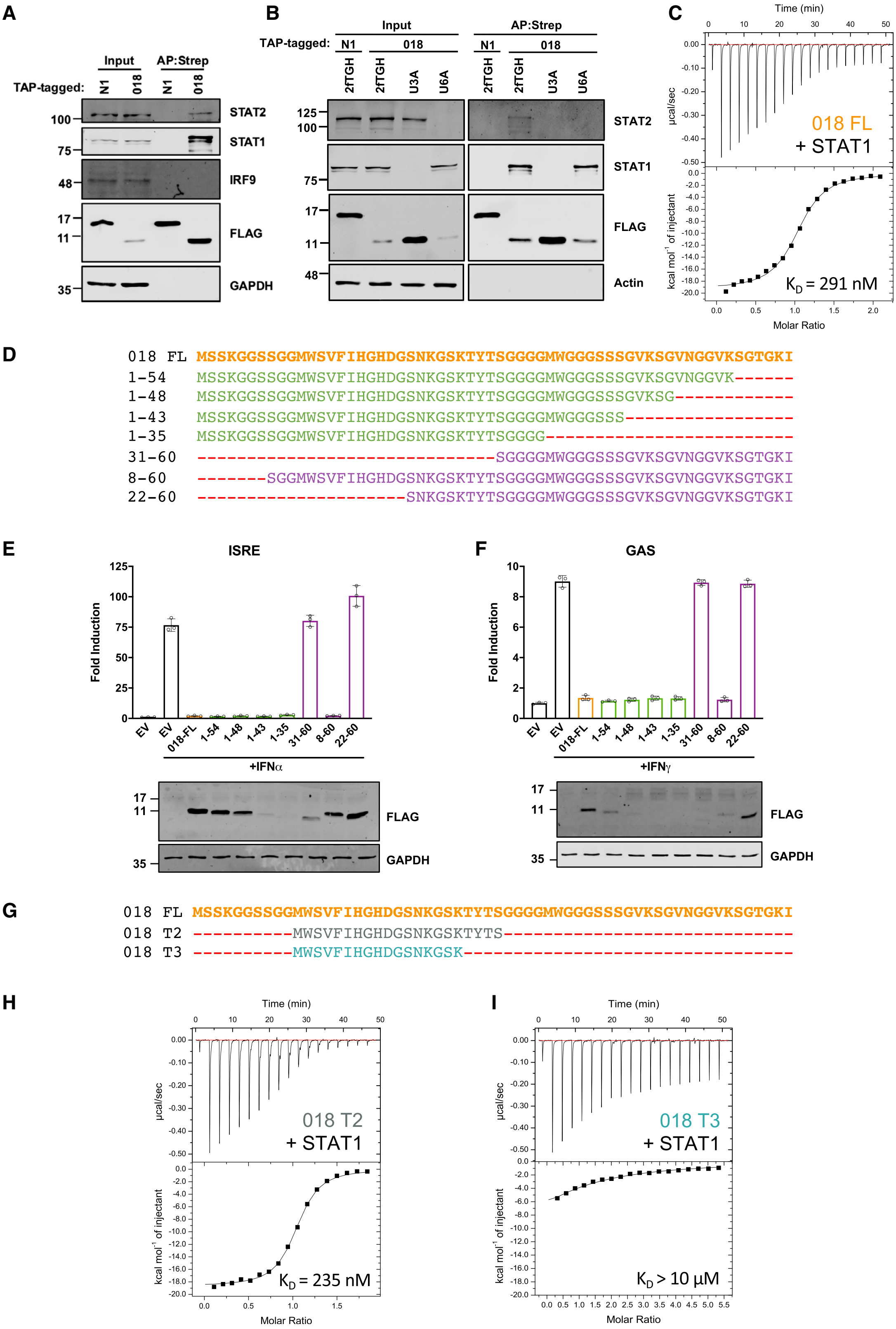

Figure 3. A 21 aa fragment of 018 is sufficient to bind STAT1

(A and B) TAP-tagged 018 and N1 were expressed in 2fTGH cells (A) or 2fTGH, U3A (STAT1$^{-/-}$) and U6A (STAT2$^{-/-}$) cells (B) by transfection and purified by Strep-Tactin. Total lysate (Input) and purified proteins (AP:Strep) were analyzed by immunoblotting.

(legend continued on next page)

ITC measurements of the minimal fragment (018^{T2}) with STAT1 gave a K_D of 235 nM, a value comparable with that of full-length 018 (291 nM) (Figure 3H). Removal of the C-terminal 28-TYTS-31 (018^{T3}) from 018^{T2} led to a large reduction in affinity (>10 μM), thereby showing the importance of these residues (Figure 3I). Collectively, these data show that a 21-residue fragment of 018 (aa 11–31) is sufficient for STAT1 binding and inhibitory activity.

018 is a virulence factor

To study the role of 018 during infection, a VACV WR 018 deletion mutant (vΔ018) was constructed. The wild-type sibling virus (v018) and vΔ018 were analyzed by PCR (Figure S4A) and genomic sequencing, which showed no differences besides the 018 deletion. Comparison of v018 and vΔ018 in cell lines competent to produce and respond to IFN (BS-C-1, A549, and RK13) displayed no difference in virus replication or plaque size (Figures S4B–S4E). Another VACV was made by reintroduction of the 018 open reading frame (ORF) fused to an N-terminal TAP tag into vΔ018 at its natural locus (vTAP-018). Pull-down of TAP-tagged 018 expressed from vTAP-018 confirmed the 018:STAT1 interaction during infection (Figures S4F and S4G).

Next, vΔ018's ability to inhibit IFN signaling was assessed. A549 cells were infected with v018 or vΔ018, then the cells were stimulated with IFN, and pSTAT1 levels were determined by immunoblotting. The cells were washed prior to stimulation to remove soluble VACV IFN decoy receptors B8 and B18. This, however, will not fully remove B18 (IFN-I decoy receptor) because it also binds to the cell surface (Alcamí et al., 2000). Although by 2 h p.i., both v018 and vΔ018 inhibited pSTAT1 induction after IFN-I stimulation, v018 inhibited earlier and to a greater extent (Figure 4A). In contrast, pSTAT1 induction was inhibited by v018 but almost fully rescued to mock levels in vΔ018-infected cells after IFN-II stimulation (Figure 4B). Consistent with this finding, STAT1 translocation was blocked by v018 after IFN-II stimulation, whereas in vΔ018-infected cells, STAT1 was mostly nuclear (Figure 4C). The impaired ability of vΔ018 to inhibit IFN-II signaling was illustrated further by increased IRF1 levels (a canonical IFNγ ISG) in cells infected with v018 compared with vΔ018 after IFN-II stimulation at both the mRNA (Figure S4H) and protein level (Figure 4D).

To test if 018 contributes to virulence, v018 and vΔ018 were compared in an intranasal mouse model. Mice infected with vΔ018 lost less weight (Figure 4E) and showed reduced virus titers at 7 and 9 days p.i. (Figure 4F). Furthermore, consistent with 018 functioning as an immunomodulator, mRNAs for several ISGs, chemokines, and IFNs were upregulated in the lungs of mice infected with vΔ018 compared with v018 (Figure 4G). Collectively, these data show that vΔ018 is defective in the inhibition of IFN-induced signaling and is attenuated in mice.

018 binds the STAT1 SH2 domain to block its association with the phosphorylated IFNGR1

To identify which STAT1 domain/s 018 binds, the interaction of 018 with several STAT1 truncations and STAT1-STAT3 chimeras was tested (Figure 5A). 018 bound a chimera with linker domain (LD), SH2, and transactivation domain (TAD) of STAT1 (31F), but not a chimera with N-terminal domain (ND), coiled-coil domain (CCD), and DNA-binding domain (DBD) of STAT1 (13F) (Figure 5B). These chimeras have been studied with NiV-V, which also only binds 31F (Rodriguez et al., 2004). 018 also bound STAT1 lacking the final 38 aa (STAT1β, a STAT1 isoform) or the entire TAD (Figure 5C). Lastly, 018 bound a chimera that contained only the SH2 and TAD of STAT1 (Fus 1) but not a chimera that contained the LD of STAT1 (Fus 2) nor with STAT3 alone (Figure 5D). Together, these data show that 018 binds the SH2 domain of STAT1.

The finding that 018 binds the STAT1 SH2 domain allowed us to hypothesize how 018 blocks STAT1 phosphorylation. Given that 018 inhibition of IFN-II signaling during infection was nonredundant, we focused on this pathway to study 018 mechanistically. The IFNGR has two IFNGR1 chains and two IFNGR2 chains that bind dimeric IFNγ (Mendoza et al., 2019). Ligand engagement induces JAK-1 phosphorylation of IFNGR1 at Tyr440 (Briscoe et al., 1996; Greenlund et al., 1994). STAT1 then docks at the IFNGR1 pTyr site via its SH2 domain and is phosphorylated (Greenlund et al., 1995), inducing its parallel dimer orientation and receptor dissociation. We hypothesized that by binding the SH2 domain, 018 blocks STAT1 recruitment to pIFNGR1 and thus prevents STAT1 phosphorylation.

To test this, a fluorescence polarization (FP) assay was set up using a fluorescent 12-mer peptide from pIFNGR1 that included the STAT1-docking site (pYDKPH). Addition of 018 to a preformed STAT1-pIFNGR1 probe led to a dose-dependent displacement of the probe and an IC_{50} value of 1.26 μM (Figure 5E). IC_{50} values of 0.93 and 17.82 μM for 018^{T2} and 018^{T3}, respectively, were obtained, demonstrating that 018^{T2}, but not 018^{T3}, has comparable inhibitory activity to full-length 018, consistent with ITC data (Figure 5E).

The mechanism was further validated by competition ITC. A 5-mer peptide of the pIFNGR1 (pYDKPH) was titrated into U-STAT1, giving a K_D of 7.6 μM (Figure 5F). In contrast, inclusion of excess 018 resulted in complete loss of detectable binding (Figure 5G). Taken together, these data demonstrate a competitive inhibition mechanism whereby 018 binds the SH2 of STAT1

(C) ITC data for GB1-018 (100 μM) titrated into U-STAT1 (10 μM). Fitting of the isotherm (bottom) to a one site model gave a K_D of 291 nM. Completely fitted ITC parameters are provided in Table S5.

(D) Sequences for TAP-tagged C-terminal (green) and N-terminal (purple) 018 truncation mutants.

(E and F) (E) HEK 293T or (F) HeLa cells were transfected with reporter plasmids ISRE-Luc (E) or GAS-Luc (F) along with *TK-Renilla* and vectors expressing proteins from (D). Cells were stimulated with IFNα (E) or IFNγ (F) for 6 (E) or 8 h (F) and then luciferase activity was measured, and lysates were analyzed by immunoblotting. Means ± SD (n = 3 per condition) are shown. Percentage inhibitory activity and relative protein expression levels from (E and F) are shown in Figure S3F. Data shown in (A and B) and (E and F) are representative of 3 or 2 individual experiments, respectively.

(G) Sequences for GB1-fused 018 truncation mutants.

(H and I) ITC data for 150 μM GB1-018^{T2} (H) or 350 μM GB1-018^{T3} (I) titrated into 15 μM STAT1. Accurate fitting of the isotherm for (I) was not possible due to the low C-value of the reaction.

CellPress
OPEN ACCESS

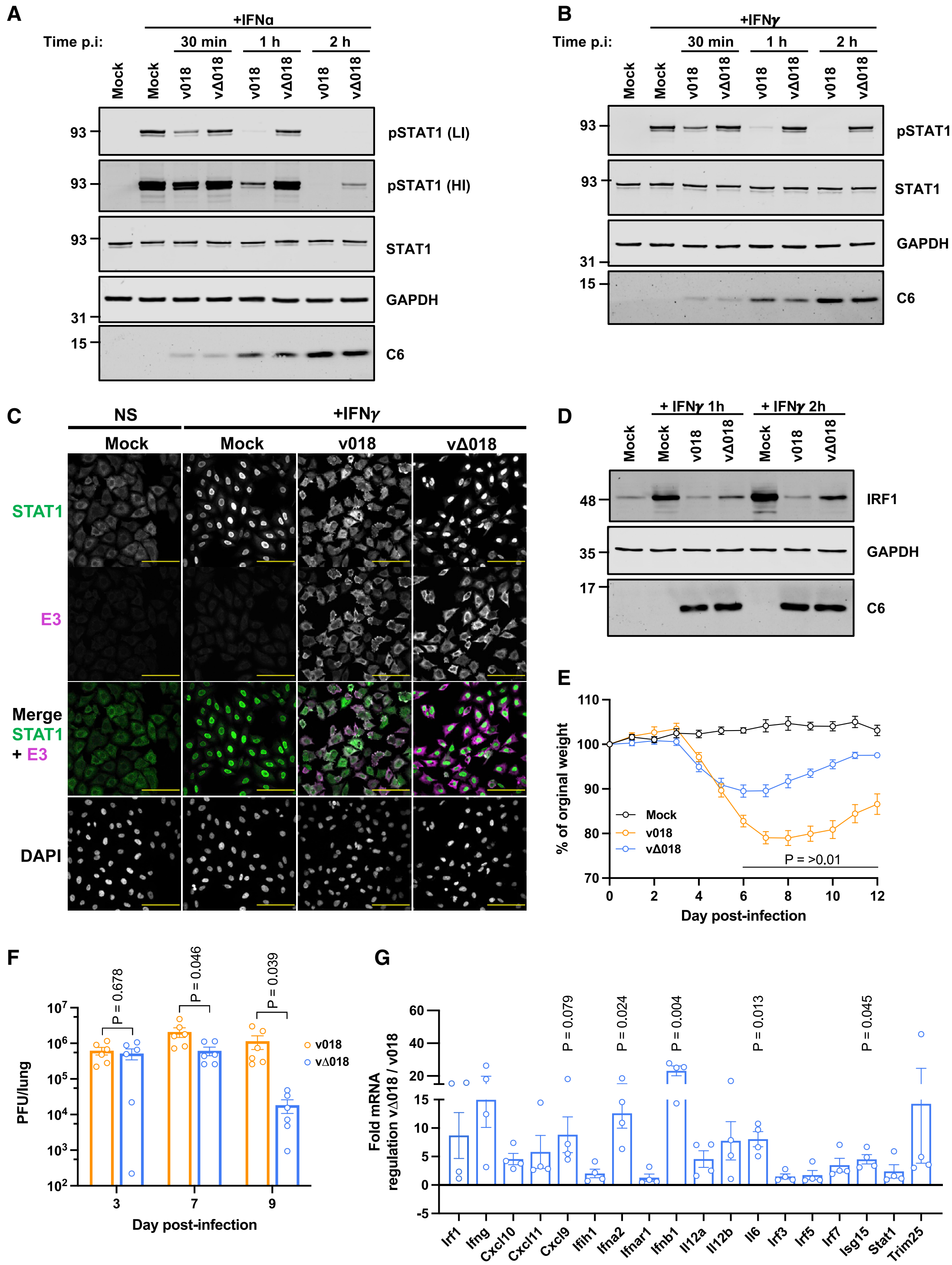

Figure 4. 018 is a virulence factor

(A and B) A549 cells were mock infected or infected with v018 or vΔ018 at 10 pfu/cell. At 30 min, 1 h, or 2 h post infection (p.i.) cells were washed once, then stimulated with IFNα (A) or IFNγ (B) for 30 min, and lysates were analyzed by immunoblotting.

(C and D) A549 cells were infected as described for (A and B) and at 2 h p.i., cells were washed once and then stimulated with IFNγ for 30 min (C), or 1 and 2 h (D). (C) Cells were immunostained with α-STAT1 and α-E3 (an VACV early protein), and DNA stained with DAPI. Cells were visualized by confocal microscopy. Scale bar (yellow) is 100 μm. (D) Cell lysates were analyzed by immunoblotting including VACV protein C6 to control for equal infection (A, B, and D). For (A), high-intensity (HI) and low-intensity (LI) scans for α-pSTAT1 are shown. Data for (A–D) are representative of 3 separate experiments.

(legend continued on next page)

and prevents STAT1 from engaging the active IFN-signaling receptor complex.

VACV 018 and NiV-V utilize a shared motif to engage STAT1

NiV-V, W and P proteins, encoded by the *P* gene, all inhibit IFN signaling. They have distinct C-terminal sequences but share a common 407 aa N-terminal region to which the IFN inhibitory activity was mapped (aa 114–140) (Ciancanelli et al., 2009). Here, we focus on this STAT1-binding region and refer to it within NiV-V.

The observation that 018 and NiV-V bind STAT1, block STAT1 phosphorylation, and bind the 31F chimera suggested that they might share a similar mode of action. Alignment of the NiV-V STAT1-binding region and 018^{T2} revealed aa similarity exemplified by a conserved HxH motif preceded by a cluster of conserved hydrophobic residues (Figure 6A). Recent ITC data showed that a NiV-V fragment (aa 92–190) binds STAT1 directly but weakly ($K_D > 100$ μM) and mutation of 117-HDH-119 to 117-AAA-119 abolished binding (Jensen et al., 2020). To determine whether the HxH motif of 018 had an analogous function, we mutated 17-HGH-19 to 17-AGA-19 (018^{AGA}). Unlike 018, 018^{AGA} did not co-precipitate with STAT1 in cells (Figure S5). Furthermore, ITC titration of 018^{AGA} into STAT1 resulted in no detectable binding (Figure 6B). Loss of STAT1 binding correlated with loss of inhibitory activity because 018^{AGA} was unable to inhibit IFN-I and -II signaling by reporter gene assay (Figures 6C and 6D). Consistent with this, 018^{AGA} did not interfere with STAT1:pIFNGR1 12-mer interaction by FP (Figure 6G). In addition, 018^{AGA} showed no inhibition of STAT1-pIFNGR1 binding via ITC (Figure 6H).

Consistent with the idea that 018 and NiV-V harbor analogous motifs, recent data showed that NiV-V binds the STAT1 SH2 domain (Keiffer et al., 2020). To test if these viral proteins target the same SH2 interface, the ability of 018 to outcompete the NiV-V:STAT1 interaction was tested. In cells, NiV-V co-precipitated with STAT1, however, this was decreased in a dose-dependent manner by the expression of 018 (Figure 6E). In contrast, 018^{AGA} did not affect the NiV-V:STAT1 interaction (Figure 6F). These data show that 018 and NiV-V utilize a shared motif to bind a common interface on the SH2 domain of STAT1.

Previous reports show that NiV-V sequesters STAT1 and 2 within the cytoplasm and prevents STAT1 phosphorylation (Rodriguez et al., 2002). The finding that 018 and NiV-V bind STAT1 via the same interface prompted us to assess if, similarly to 018, NiV-V competes with pIFNGR1 to bind STAT1. To test this, NiV-V STAT1-binding fragment residues 110–140 (NiV-$V^{110-140}$) fused to a GB1 tag was purified together with a mutant in which His117 and His119 of the HxH motif were mutated to Ala (NiV-V^{ADA}). By FP assay, addition of NiV-V to the preformed STAT1-pIFNGR1 12-mer complex led to a modest reduction in polarization, whereas addition of NiV-V^{ADA} was non-competitive (Figure 6I). Consistent with these data, preincubation of STAT1 with NiV-V abolished any detectable binding between STAT1 and the pIFNGR 5-mer by ITC (Figure 6J). In contrast, preincubation with NiV-V^{ADA} did not prevent STAT1:pIFNGR binding (Figure 6K). These data show that in the context of IFN-II signaling, NiV-V can block STAT1 recruitment to the active IFNGR signaling complex.

Phosphotyrosine pocket-independent binding of 018 to the STAT1 SH2 domain

A feature of the SH2 interface is a deep pTyr pocket that binds the phosphate group and the phenyl ring of pTyr. Remarkably, 018 binds the STAT1 SH2 domain with high affinity and competes with pIFNGR1 without a pTyr modification. Intrigued by this, we crystallized the STAT1 core (aa 132-684) complexed with the minimal 21-mer 018 peptide (Met11-Ser31). Crystals diffracted to 2.0 Å with 018 electron density clearly defined except for Ser31 (Figure S6A).

The 018 peptide forms a β-hairpin with a β-turn midway through the sequence (Figures 7A and 7B). The two peptide strands augment the central β-sheet of the SH2 domain, with the 018 Val14-His17 backbone hydrogen bonding to the βD strand of the SH2 domain (Figure 7C). There is spatial overlap with the published binding modes of pTyr peptides from pIFNGR1 and pSTAT1 homodimer (Figure 7B). The 680 $Å^2$ 018-STAT1 interface is formed by multiple shallow contacts exclusively within the SH2 domain. 018 Trp12, Val14, and Ile16 form a continuous hydrophobic interface with STAT1 helix αA and strand βD (Figure 7D). This is followed by the HxH motif, in which His17 forms an imidazole-to-imidazole hydrogen bond with His629 of STAT1 (Figures 7D and 7E). The His17 rotamer is stabilized intramolecularly by a second hydrogen bond with the backbone carbonyl of 018 Gly21. Gly18 carbonyl forms a hydrogen bond with the Tyr651 hydroxyl of STAT1, similar to pIFNGR1 Pro443 (PDB: 1YVL). His19 occupies the same cleft as His444 of pIFNGR1, forming an identical π-stacking interaction with STAT1 Tyr634. The Asp20 sidechain stabilizes the β-turn by hydrogen bonding with the Ser21 backbone and forms an intramolecular salt bridge with Lys24 (Figure 7D). An inter-strand hydrogen bond between the hydroxyl groups of Ser13 and Thr28 acts as a non-covalent bridge that may stabilize the β-hairpin fold (Figure 7D).

Strikingly, 018 does not interact with the pTyr pocket. The only Tyr in the 018 peptide, Tyr29, hydrogen bonds with the ζ-amine of STAT1 Lys584 through its hydroxyl and makes van der Waals contacts with the alkyl chain of the same lysine (Figure 7D). The lower affinity of 018^{T3} compared with that of 018^{T2} may result from the loss of interactions made by Thr28 and Tyr29. The effect of phosphorylation at Tyr29 was tested by comparing the affinity of an 018 peptide phosphorylated at Tyr29 (p018 21-mer) with that of the unphosphorylated 018 peptide (018 21-mer) for STAT1 by ITC. The phosphopeptide had a slight increased affinity (<2-fold) of 174 nM compared with the unmodified 018 peptide (321 nM) (Figures S6B and S6C). Based on

(E–G) BALB/c mice were infected intranasally with v018 (orange) or vΔ018 (blue) at 10^3 (E and F) or 10^5 (G) pfu and weighed daily (E) or virus titers in upper lung lobes were titrated by plaque assay on days 3, 7, and 9 p.i. (F), or mice were sacrificed at 3 days p.i. and mRNA levels of indicated genes from upper lung lobes were analyzed by RT-qPCR (G). Data from (E and F) are representative of at 2 individual experiments using 5 or 3 mice, respectively, per group that were then pooled. Data from (G) are representative of 4 (vΔ018) or 3 (v018) mice per group. For (E–G) means ± SEM are shown, and p values were calculated using unpaired t test with (E and F) or without (G) Welch's correction.

CellPress
OPEN ACCESS

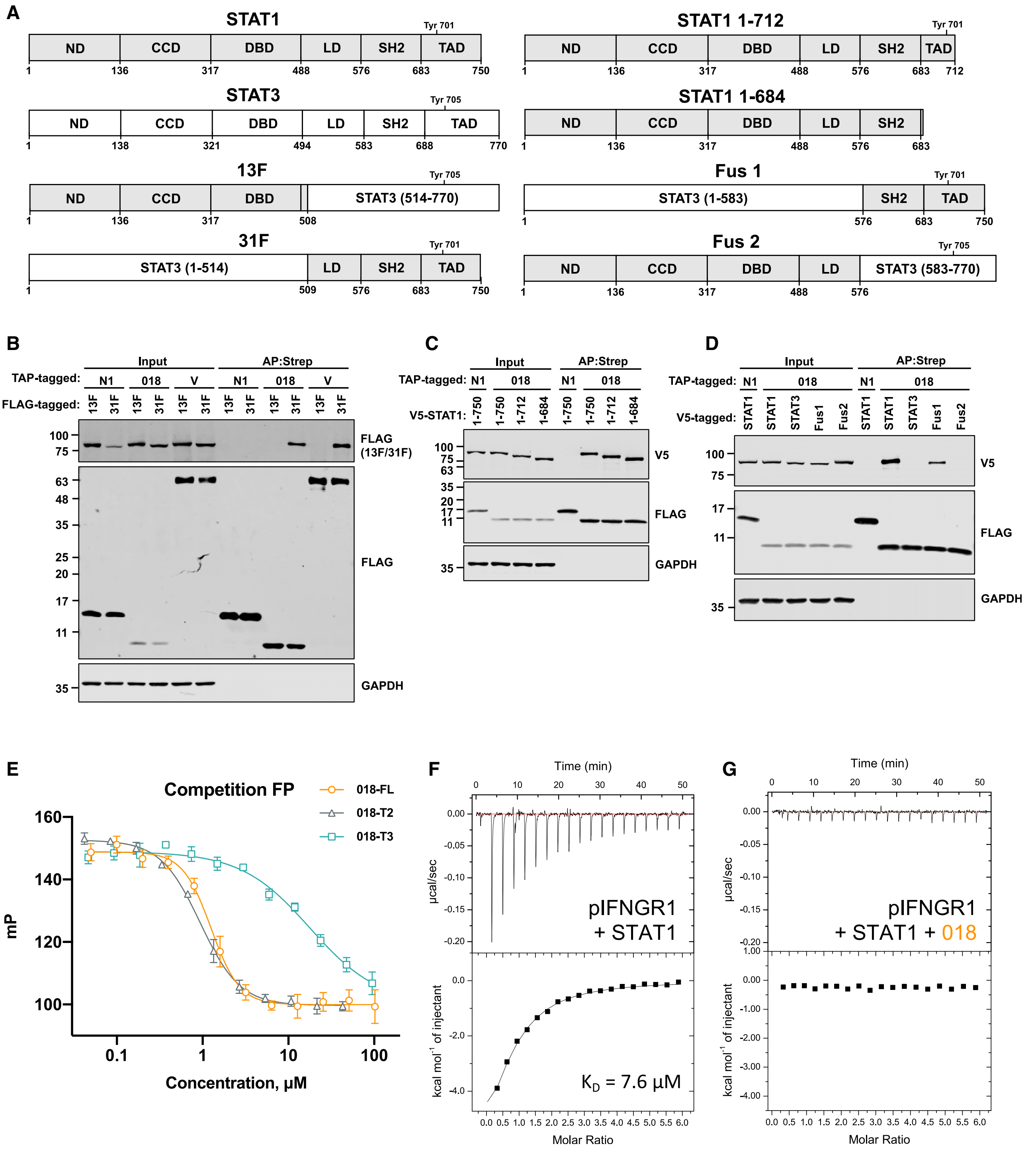

Figure 5. 018 binds the STAT1 SH2 domain to block its association with the phosphorylated IFNGR1

(A) Schematic of STAT1-STAT3 chimeras and STAT1 truncation mutants. STAT1 regions (gray) and STAT3 (white) are shown.

(B–D) TAP-tagged proteins indicated were co-expressed with either FLAG (B) or V5-tagged (C and D) STAT proteins from (A) by transfection in U3A (STAT1$^{-/-}$) cells and TAP-tagged proteins were purified by Strep-Tactin. Total lysates (Input) and purified proteins (AP:Strep) were analyzed by immunoblotting. Data from (B–D) are representative of 2 individual experiments.

(E) Competition FP measurements for GB1-018 and truncation mutants. Each reaction contained 10 nM fluorescein-pIFNGR1 12-mer preincubated with 1.5 μM U-STAT1, to which 2-fold serial dilutions of GB1-018 proteins were added. 100 mP represents the calibrated FP value of the free fluorescent probe.

(F and G) ITC data for 300 μM pIFNGR1 5-mer titrated into 10 μM U-STAT1 (F) or 10 μM U-STAT1 preincubated with 50 μM GB1-018 (G). No heat of binding was detected for (G).

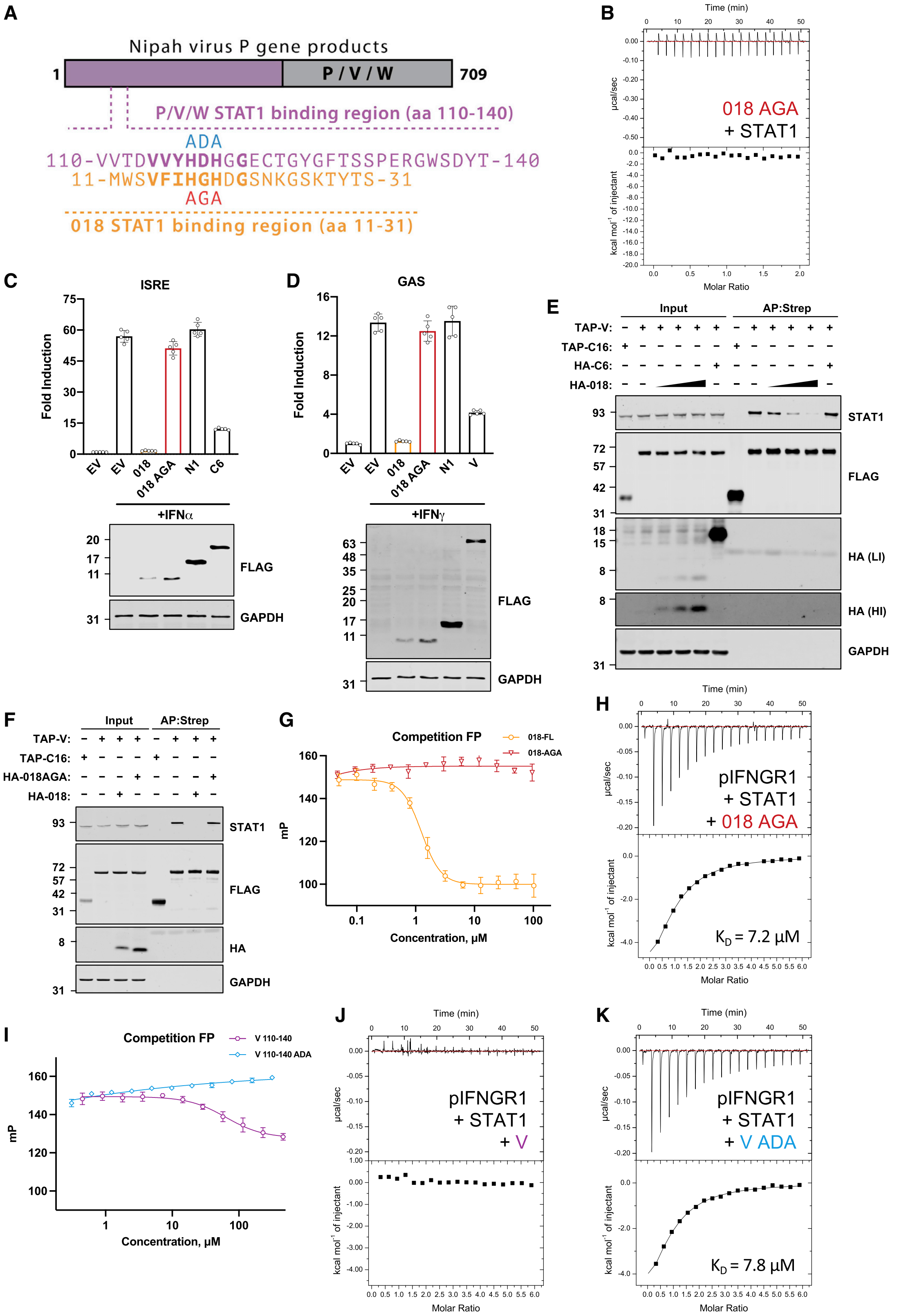

(legend on next page)

CellPress
OPEN ACCESS

the 018:STAT1 structure, binding of pTyr29 into the pTyr pocket would require substantial rearrangement of the peptide conformation. Such a rearrangement would be incompatible with the binding of the essential HxH motif. The slightly increased affinity likely stems from an inter-molecular salt bridge forming between pTyr29 and STAT1 Lys584, and an intramolecular salt bridge with 018 Lys27, both situated near the Tyr29 sidechain. Immunoblotting with a pTyr antibody after pull-down enrichment provided no evidence of 018 Tyr phosphorylation, either in resting cells, cells stimulated with IFN-I/II, or during VACV infection (Figures S6D–S6F). These data are consistent with physiological 018 not being Tyr phosphorylated. It remains possible that Tyr29 is phosphorylated at low levels in cells, but considering the minor affinity difference between the two forms, it is unlikely that pTyr-018 would contribute significantly to STAT1 occupancy.

A single histidine found in STAT1 and 4 determines 018 selectivity

High sequence similarity between SH2 domains of STATs led us to investigate if 018 interacts with other STATs. In humans, there are seven STATs (STAT1, 2, 3, 4, 5A, 5B, and 6) (Ihle, 2001). Pull-down of 018 from U3A cells demonstrated that 018 binds STAT1 and 4, but not other STATs (Figure 7F). To understand the observed specificity of 018 for STAT1 and 4, STAT SH2 domain alignments were integrated with our structural data (Figure S6G). In the structure, an interaction between 018 His17 and STAT1 His629 was observed. Only STAT1 and 4 have a His at this position, and so other STATs fail to recapitulate this interaction. To test if STAT1 His629 was critical for specificity, a STAT3 mutant was made in which the structurally equivalent Glu635 was mutated to His. This enabled 018 to co-precipitate STAT3^{Q635H}, confirming the assignment of this specificity determinant for 018 binding (Figure 7G).

DISCUSSION

STAT1 and 2 are central to IFN signaling and thus are common targets for viral antagonism (Harrison and Moseley, 2020); however, structural details of STAT:antagonist complexes are few. The complex of SeV C protein with the ND of STAT1 indicates that the C protein interferes with the oligomeric state of STAT1 (Oda et al., 2015), while the structures of dengue and Zika virus NS5 proteins in complex with STAT2 reveal that their NS5 proteins overlap the IRF9 binding site to prevent the ISGF3 assembly (Wang et al., 2020). A similar mechanism was described for measles V protein (Nagano et al., 2020). Here, the complex of poxvirus protein 018 with STAT1, shows that 018 occupies the STAT1 SH2 domain to block STAT1 association with the active IFNGR.

Interaction between STAT SH2 domains and pTyr sites is a common mechanism for STAT recruitment to activated receptors. The pTyr contributes half the binding energy, whereas specificity is provided by a small number of adjacent residues (Kaneko et al., 2010; Ladbury and Arold, 2011). For STAT1, 018 overlaps with these specificity-determining sites and obstructs the pTyr pocket without occupying it.

To establish if a binding mode similar to 018:STAT1 exists, we examined 524 SH2-containing structures retrieved from PDB. Most liganded SH2 domains bind a pTyr-containing peptide, a synthetic pTyr mimetic or an unphosphorylated Tyr at the pTyr pocket. Several structures contain SH2 domains as part of a larger protein-protein interaction, in which the pTyr pocket is not occupied; however, in such cases, the interface extends significantly beyond the SH2 phosphopeptide site. The closest binding mode analog to 018 was a monobody that binds at the phosphopeptide site of SHP-1 phosphatase without interacting with the pTyr pocket (PDB: 6SM5). Hence, we suggest that 018 has an unprecedented mode of high-affinity SH2 domain binding.

For IFN-I-induced signaling, 018 blocked pSTAT1 induction but only reduced pSTAT2 levels slightly. While it is accepted that STAT2 associates constitutively with the IFNAR2 chain (Li et al., 1997; Shemesh et al., 2021), the proceeding steps that induce STAT1/2 phosphorylation at the IFNAR are unclear. One model suggests that STAT2 is phosphorylated after docking at an IFN-I induced pTyr site on IFNAR1 (Tyr466) and that subsequently, the pTyr690 of STAT2 provides a docking site for STAT1 via its SH2 domain (Leung et al., 1995; Li et al., 1997; Qureshi et al., 1996; Yan et al., 1996). Additional Tyr phosphorylation sites on IFNAR2 and 1 may also provide docking sites for STAT1 and 2 functioning in a cell type- or species-dependent manner (Zhao et al., 2008). Thus, we rationalize that the occupancy of the STAT1 SH2 domain by 018 could diminish

Figure 6. VACV 018 and NiV-V protein utilize a shared motif to engage STAT1

(A) Schematic of Nipah virus P, V, and W proteins indicating the common N-terminal region (purple) and unique C-terminal region (gray). Below, the STAT1-binding regions of P/V/W (residues 110–140, purple) and 018 (residues 11–31, orange) are aligned with key conserved residues emboldened. Sites of NiV-V^{ADA} (blue) and 018^{AGA} (red) mutants are shown.

(B) ITC data for the titration of 100 μM GB1-018^{AGA} into 10 μM U-STAT1. No heat of binding was observed.

(C and D) (C) HEK 293T or (D) HeLa cells were transfected with plasmids ISRE-Luc (C) or GAS-Luc (D) along with *TK-Renilla* and vectors expressing the indicated TAP-tagged proteins. Cells were stimulated with IFNα (C) or IFNγ (D) for 6 h (C) or 8 h (D), then luciferase activity was measured, and lysates were analyzed by immunoblotting. Means ± SD (n = 5 per condition) are shown.

(E and F) TAP-tagged and HA-tagged proteins were co-expressed in HEK 293T cells by transfection as indicted and TAP-tagged proteins were purified by Strep-Tactin. Total lysates (Input) and purified (AP:Strep) proteins were analyzed by immunoblotting. For (E), high-intensity (HI) and low-intensity (LI) scans are shown for α-HA. VACV proteins TAP-C16 and HA-C6 were used as a pull-down and competition protein controls, respectively. Data shown in (C and D) and (E and F) are representative of 2 or 3 individual experiments, respectively.

(G and I) Competition FP measurements for GB1-018 and GB1-018^{AGA} (G) or GB1-NiV-V and GB1-NiV-V^{ADA} (I) binding to U-STAT1. Each reaction contained 10 nM fluorescein-pIFNGR1 12-mer preincubated with 1.5 μM U-STAT1, to which 2-fold serial dilutions of GB1 proteins were added. 100 mP represents the calibrated FP value of the free fluorescent probe. The NiV-V^{ADA} curve has a positive slope at high protein concentrations due to increased sample viscosity or non-specific interactions (I).

(H, J, and K) ITC data for 300 μM pIFNGR1 5-mer titrated into 10 μM U-STAT1 preincubated with 50 μM GB1-018^{AGA} (H), 200 μM NiV-V (J), or 200 μM NiV-V^{ADA} (K). No heat of binding was detected for the reaction containing GB1-NiV-V.

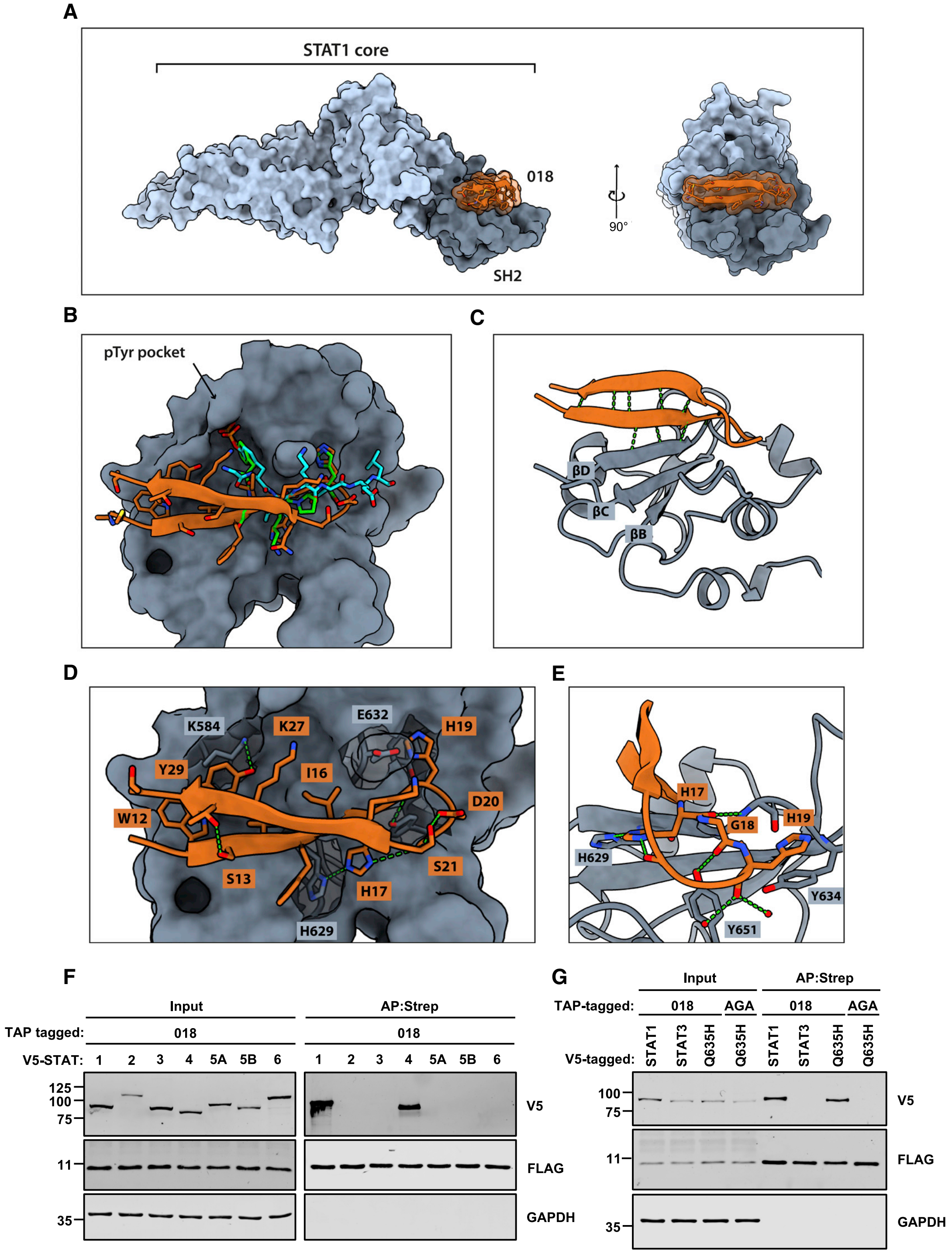

Figure 7. Structural basis of 018 binding to U-STAT1

(A–E) Crystal structure of the 018:STAT1 core complex (PDB: 7nuf). 018 is depicted in orange, the SH2 domain is dark gray, and the rest of the core is light gray. (A) Surface view of the complex from two perpendicular axes. (B) 018 binding mode at the STAT1 SH2 domain superimposed with IFNGR1 phosphopeptide (green, PDB: 1yvl) and STAT1 pTyr701 phosphopeptide (cyan, PDB: 1bf5). (C) Ribbon diagram of 018 and the STAT1 SH2 domain with β-sheet-forming hydrogen bonds shown in green. SH2 domain core β-strands are labeled with standard nomenclature. (D) Detailed depiction of 018 binding to the STAT1 SH2 domain. 018 sidechains are shown as sticks and backbone atoms as ribbons. Key STAT1 sidechains are depicted as sticks under semi-transparent surface. (E) A zoomed-in view of HxH motif binding. (F and G) V5-tagged and TAP-tagged proteins were co-expressed in U3A (STAT1$^{-/-}$) cells by transfection as indicted and purified using Strep-Tactin. Total lysates (Input) and purified proteins (AP:Strep) were analyzed by immunoblotting. STAT3^{Q635H} and 018^{AGA} are labeled as Q635H and AGA, respectively (G). Data shown in (F and G) are representative of 2 individual experiments.

CellPress
OPEN ACCESS

STAT1 engagement of either STAT2 pTyr690 or pTyr sites on IFNAR to prevent STAT1 phosphorylation. Alternatively, a recent study suggested that STAT1 and 2 are weakly phosphorylated independently of pTyr receptor sites and instead serve to enhance the dissociation of STATs from IFNAR2, thereby increasing the flux of STAT phosphorylation (Shemesh et al., 2021). Irrespective of the model, our data supports the SH2 domain of STAT1 being important for its IFN-I induced phosphorylation.

STAT4 was identified as an additional binding partner of 018. STAT4 is activated by phosphorylation mostly in response to IL-12 and IFN-I and promotes IFNγ production during viral infection (Nguyen et al., 2002; Yang et al., 2020). The activation of STAT4 occurs mainly in lymphoid and myeloid cells but also in vascular endothelial cells (Torpey et al., 2004); thus, for 018 to modulate this pathway, VACV would need to infect these cell types *in vivo*. The conserved 018-binding interface between STAT1 and 4 suggests that 018 could prevent STAT4 recruitment to its activating receptors. Whether the 018:STAT4 interaction plays a physiological role during infection remains to be determined.

The 018 STAT1-binding region shares similarity to the STAT1-binding region of V/W and P proteins from NiV, a paramyxovirus discovered in Malaysia in 1998 (Chua et al., 2000). NiV is highly pathogenic in humans, causing numerous sporadic outbreaks, including recently in Kerala, India (Arunkumar et al., 2019), and no effective treatments or vaccines are available (Hauser et al., 2021). The observation that the STAT1-binding region can block STAT1:pIFNGR1 association is most relevant to the V and P proteins due to their cytoplasmic location (Shaw et al., 2004). Although W harbors an identical STAT1-binding region, it traffics STAT1 to the nucleus to prevent STAT1 activation (Shaw et al., 2004). As we anticipate that the 114-VVYHDHGG-121 region of NiV-V/W and P binds in an analogous fashion to the 14-VFIHGHDG-21 of 018, the 018:STAT1 structure can aid the understanding of previous mutagenesis studies of the NiV STAT1-binding region (Ciancanelli et al., 2009; Hagmaier et al., 2006; Jensen et al., 2020; Ludlow et al., 2008; Satterfield et al., 2019).

Intrinsically disordered proteins that harbor short linear motifs (SLiMs), such as the STAT1-binding region from NiV-V and 018, are important mediators of virus-host interactions (Mishra et al., 2020). SLiMs are advantageous to viruses because they offer high flexibility and typically evolve rapidly, allowing quick adaptation to changing host environments (Xue et al., 2014). Virus SLiMs that mimic eukaryotic linear motifs are a prevalent virus strategy to hijack cellular machinery and disable host defenses (Davey et al., 2011; Hagai et al., 2014; Lasso et al., 2021). Because SLiMs are short and evolve easily, they have emerged mostly independent of their host mimics rather than by horizontal gene transfer (Elde and Malik, 2009; Hagai et al., 2014). In the context of the 018/NiV-V STAT1-binding motif, although possible cellular proteins exist that bind STAT SH2 domains in a pTyr-independent manner, none have been identified. The STAT1-binding motif, described here, likely represents a striking example of convergent evolution in diverse virus families and has produced an unconventional binding mechanism to target STAT1. Consistent with the notion that SLiMs preferentially target proteins central to multiple networks (Dyer et al., 2008), STAT1 is required for ISG induction in response to all IFN families (IFN-I, -II, and -III). The existence of the shared motif between disparate viruses highlights its importance as an efficient moiety for inhibiting IFN-induced signaling.

Poxviruses encode multiple antagonists of IFN-induced signaling. Of these, the viral phosphatase vH1 is released into the cytoplasm immediately upon infection, where it might dephosphorylate STAT1, although this activity was shown only *in vitro* (Najarro et al., 2001; Schmidt et al., 2013). Multiple reports show that shortly after VACV infection, the cells are refractory to pSTAT1 activation by IFN-II stimulation (Mann et al., 2008; Najarro et al., 2001; Schmidt et al., 2013). Hitherto, this phenotype was mainly attributed to vH1; however, deletion of 018 led to an almost complete rescue of pSTAT1 levels despite the presence of vH1, demonstrating that during infection 018, rather than vH1, is responsible for this phenotype. Consistent with this early block, 018 is one of the earliest viral proteins expressed during infection (Soday et al., 2019).

Despite apparent redundancy in inhibition of IFN-induced signaling by VACV, deletion of individual IFN antagonists leads to virus attenuation *in vivo* (Figure 4; Symons et al., 1995; Unterholzner et al., 2011). These non-redundant phenotypes may stem from each inhibitor having different locations, expression kinetics, or being multifunctional. Unlike intracellular inhibitors, B18 and B8 are secreted from cells and can thus neutralize IFNs extracellularly and distally. Also, B18 can bind to cell surface glycosaminoglycans and thereby inhibit IFN-I-induced signaling in uninfected cells (Alcamí et al., 2000; Montanuy et al., 2011). This is the major mechanism by which B18 promotes virulence (Hernáez et al., 2018). Although no VACV-specific inhibitor of IFN-III-induced signaling has been described, cells infected with VACV are refractory to pSTAT1 induction after IFN-III stimulation, and IFN-III expression during viral infection has little effect on VACV replication (Bandi et al., 2010; Bartlett et al., 2005). These observations may be explained by the action of 018. Differences in the expression kinetics of VACV IFN antagonists could also affect redundancy, for, although B18 functions upstream of 018, VACV lacking 018 showed enhanced levels of pSTAT1 after IFN-I stimulation. Lastly, virus proteins are often multifunctional. Indeed, VACV protein C6, which inhibits both IFN production (Unterholzner et al., 2011) and IFN-I signaling (Stuart et al., 2016), also degrades VACV restriction factors HDAC4 and 5 (Lu et al., 2019; Soday et al., 2019). It remains unknown if the attenuated phenotype of the 018-deletion virus derives from the ability of 018 to bind STAT1, STAT4, or both proteins, or to additional other unknown function of 018.

Deletion of IFN antagonists can improve the safety and immunogenicity of VACV-based vaccine vectors (Albarnaz et al., 2018). Modified vaccinia Ankara (MVA) is a widely used vaccine vector and expresses 018 (Wennier et al., 2013). MVAs expressing SARS-CoV-2 proteins have been described as potential vaccine candidates, and thus, our findings can inform further development (Chiuppesi et al., 2020; García-Arriaza et al., 2021; Liu et al., 2021).

In summary, we describe a viral mechanism to antagonize IFN-induced signaling by occupancy of the STAT1 SH2 domain to prevent STAT1-receptor association. The structure of the VACV protein 018 complexed with STAT1 illustrates how a viral protein has evolved an unconventional strategy to bind an SH2 domain with high affinity. The biological importance of 018 is

shown by its contribution to virus virulence despite additional IFN antagonists. Finally, this study highlights that disparate viruses can evolve highly similar motifs to target a host response that poses a common threat to all viruses.

STAR★METHODS

Detailed methods are provided in the online version of this paper and include the following:

- KEY RESOURCES TABLE
- RESOURCE AVAILABILITY
  - Lead contact
  - Materials availability
  - Data and code availability
- EXPERIMENTAL MODEL AND SUBJECT DETAILS
  - Cell lines
  - Viruses
  - Animals
- METHOD DETAILS
  - Orthologue alignments
  - Plasmids
  - Construction of T-REx 293 cell lines expressing proteins inducibly
  - Construction of recombinant VACVs
  - Purification of VACVs by sedimentation through sucrose
  - VACV infection for cell culture
  - Virus growth and spread assays
  - Interferon stimulations
  - Immunoblotting
  - Reporter gene assays
  - Immunofluorescence
  - RT-qPCR
  - Pulldowns
  - *In vivo* experiments
  - Protein expression and purification
  - Isothermal titration calorimetry (ITC)
  - Fluorescence polarisation (FP) anisotropy measurements
  - Peptides for ITC and FP
  - SEC-MALS
  - X-ray crystallography
- QUANTIFICATION AND STATISTICAL ANALYSIS

SUPPLEMENTAL INFORMATION

Supplemental information can be found online at https://doi.org/10.1016/j.chom.2022.01.014.

ACKNOWLEDGMENTS

This work was supported by a Wellcome Trust Principal Research Fellowship (090315, to G.L.S.). C.T.-C. was funded by the BBSRC DTP. T.P. was funded by the MRC DTP. M.T.A. was funded by a Harry Smith vacation studentship from the Microbiology Society. J.P.S., C.R.C., and H.D.H. were supported by the NIAID Division of Intramural Research. We thank Diamond Light Source for access to macromolecular crystallography beam line i04 (proposal 25402) and the Biophysical research facilities at the Department of Biochemistry, University of Cambridge, for access to instrumentation and support. Funding: Acquisition: M.H., H.D.H., and G.L.S.

AUTHOR CONTRIBUTIONS

Conceptualization, C.T.-C., T.P., and G.L.S.; methodology, C.T.-C., T.P., and J.P.S.; formal analysis, C.T.-C., T.P., and J.P.S.; investigation, C.T.-C., T.P., J.P.S., C.R.C., and M.T.A.; resources, T.P., M.H., H.D.H., and G.L.S.; data curation, C.T.-C., T.P., J.P.S., and M.H.; writing: original draft, C.T.-C. and T.P.; writing: reviewing and editing, C.T.-C., T.P., J.P.S., C.R.C., M.T.A., M.H., H.D.H., and G.L.S.; visualization, C.T.-C. and T.P.; supervision, M.H., H.D.H., and G.L.S.; project administration, C.T.-C. and G.L.S.

DECLARATION OF INTERESTS

The authors declare no competing interests.

Received: July 29, 2021
Revised: October 29, 2021
Accepted: January 10, 2022
Published: February 18, 2022

REFERENCES

Aaronson, D.S., and Horvath, C.M. (2002). A road map for those who don't know JAK-STAT. Science *296*, 1653–1655.

Albarnaz, J.D., Torres, A.A., and Smith, G.L. (2018). Modulating vaccinia virus immunomodulators to improve immunological memory. Viruses *10*, 101.

Alcamí, A., and Smith, G.L. (1995). Vaccinia, cowpox, and camelpox viruses encode soluble gamma interferon receptors with novel broad species specificity. J. Virol. *69*, 4633–4639.

Alcamí, A., Symons, J.A., and Smith, G.L. (2000). The vaccinia virus soluble alpha/beta interferon (IFN) receptor binds to the cell surface and protects cells from the antiviral effects of IFN. J. Virol. *74*, 11230–11239.

Arunkumar, G., Chandni, R., Mourya, D.T., Singh, S.K., Sadanandan, R., Sudan, P., and Bhargava, B.; Nipah Investigators People and Health Study Group (2019). Outbreak investigation of Nipah virus disease in Kerala, India, 2018. J. Infect. Dis. *219*, 1867–1878.

Assarsson, E., Greenbaum, J.A., Sundström, M., Schaffer, L., Hammond, J.A., Pasquetto, V., Oseroff, C., Hendrickson, R.C., Lefkowitz, E.J., Tscharke, D.C., et al. (2008). Kinetic analysis of a complete poxvirus transcriptome reveals an immediate-early class of genes. Proc. Natl. Acad. Sci. USA *105*, 2140–2145.

Bandi, P., Pagliaccetti, N.E., and Robek, M.D. (2010). Inhibition of type III interferon activity by orthopoxvirus immunomodulatory proteins. J. Interferon Cytokine Res. *30*, 123–134.

Bartlett, N.W., Buttigieg, K., Kotenko, S.V., and Smith, G.L. (2005). Murine interferon lambdas (type III interferons) exhibit potent antiviral activity in vivo in a poxvirus infection model. J. Gen. Virol. *86*, 1589–1596.

Briscoe, J., Rogers, N.C., Witthuhn, B.A., Watling, D., Harpur, A.G., Wilks, A.F., Stark, G.R., Ihle, J.N., and Kerr, I.M. (1996). Kinase-negative mutants of JAK1 can sustain interferon-gamma-inducible gene expression but not an antiviral state. EMBO J *15*, 799–809.

Chiuppesi, F., Salazar, M., d'Alincourt, C.H., Nguyen, V.H., Martinez, J., Park, Y., Nguyen, J., Kha, M., Iniguez, A., et al. (2020). Development of a multi-antigenic SARS-CoV-2 vaccine candidate using a synthetic poxvirus platform. Nat. Commun. *11*, 6121.

Chua, K.B., Bellini, W.J., Rota, P.A., Harcourt, B.H., Tamin, A., Lam, S.K., Ksiazek, T.G., Rollin, P.E., Zaki, S.R., Shieh, W., et al. (2000). Nipah virus: a recently emergent deadly paramyxovirus. Science *288*, 1432–1435.

Ciancanelli, M.J., Volchkova, V.A., Shaw, M.L., Volchkov, V.E., and Basler, C.F. (2009). Nipah virus sequesters inactive STAT1 in the nucleus via a P gene-encoded mechanism. J. Virol. *83*, 7828–7841.

Colamonici, O.R., Domanski, P., Sweitzer, S.M., Larner, A., and Buller, R.M.L. (1995). Vaccinia virus B18R gene encodes a type I interferon-binding protein that blocks interferon α transmembrane signaling. J. Biol. Chem. *270*, 15974–15978.

Davey, N.E., Travé, G., and Gibson, T.J. (2011). How viruses hijack cell regulation. Trends Biochem. Sci. *36*, 159–169.

CellPress
OPEN ACCESS

Dyer, M.D., Murali, T.M., and Sobral, B.W. (2008). The landscape of human proteins interacting with viruses and other pathogens. PLoS Pathog. *4*, e32.

Elde, N.C., and Malik, H.S. (2009). The evolutionary conundrum of pathogen mimicry. Nat. Rev. Microbiol. *7*, 787–797.

Emsley, P., Lohkamp, B., Scott, W.G., and Cowtan, K. (2010). Features and development of Coot. Acta Crystallogr. D Biol. Crystallogr. *66*, 486–501.

Falkner, F.G., and Moss, B. (1990). Transient dominant selection of recombinant vaccinia viruses. J. Virol. *64*, 3108–3111.

García-Arriaza, J., Garaigorta, U., Pérez, P., Lázaro-Frías, A., Zamora, C., Gastaminza, P., del Fresno, C., Casasnovas, J.M., Sorzano, S., Ó., C., Sancho, D., and Esteban, M. (2021). COVID-19 vaccine candidates based on modified vaccinia virus Ankara expressing the SARS-CoV-2 spike protein induce robust T- and B-cell immune responses and full efficacy in mice. J. Virol. *95*, e02260-20.

García-Sastre, A. (2017). Ten strategies of interferon evasion by viruses. Cell Host Microbe *22*, 176–184.

Greenlund, A.C., Farrar, M.A., Viviano, B.L., and Schreiber, R.D. (1994). Ligand-induced IFN(γ) receptor tyrosine phosphorylation couples the receptor to its signal transduction system (p91). EMBO J *13*, 1591–1600.

Greenlund, A.C., Morales, M.O., Viviano, B.L., Yan, H., Krolewski, J., and Schreiber, R.D. (1995). Stat recruitment by tyrosine-phosphorylated cytokine receptors: an ordered reversible affinity-driven process. Immunity *2*, 677–687.

Gubser, C., Hué, S., Kellam, P., and Smith, G.L. (2004). Poxvirus genomes: A phylogenetic analysis. J. Gen. Virol. *85*, 105–117.

Hagai, T., Azia, A., Babu, M.M., and Andino, R. (2014). Use of host-like peptide motifs in viral proteins is a prevalent strategy in host-virus interactions. Cell Rep *7*, 1729–1739.

Hagmaier, K., Stock, N., Goodbourn, S., Wang, L.F., and Randall, R. (2006). A single amino acid substitution in the V protein of Nipah virus alters its ability to block interferon signalling in cells from different species. J. Gen. Virol. *87*, 3649–3653.

Harrison, A.R., and Moseley, G.W. (2020). The dynamic interface of viruses with STATs. J. Virol. *94*, e00856-20.

Hauser, N., Gushiken, A.C., Narayanan, S., Kottilil, S., and Chua, J.V. (2021). Evolution of Nipah virus infection: past, present, and future considerations. Trop. Med. Infect. Dis. *6*, 24.

Hernáez, B., Alonso-Lobo, J.M., Montanuy, I., Fischer, C., Sauer, S., Sigal, L., Sevilla, N., and Alcamí, A. (2018). A virus-encoded type I interferon decoy receptor enables evasion of host immunity through cell-surface binding. Nat. Commun. *9*, 5440.

Ihle, J.N. (2001). The Stat family in cytokine signaling. Curr. Opin. Cell Biol. *13*, 211–217.

Jensen, M.R., Yabukarski, F., Communie, G., Condamine, E., Mas, C., Volchkova, V., Tarbouriech, N., Bourhis, J.M., Volchkov, V., Blackledge, M., and Jamin, M. (2020). Structural description of the Nipah virus phosphoprotein and its interaction with STAT1. Biophys. J. *118*, 2470–2488.

Johnson, M., Zaretskaya, I., Raytselis, Y., Merezhuk, Y., McGinnis, S., and Madden, T.L. (2008). NCBI BLAST: a better web interface. Nucleic Acids Res *36*, W5–W9.

Joklik, W.K. (1962). The purification of four strains of poxvirus. Virology *18*, 9–18.

Kaneko, T., Huang, H., Zhao, B., Li, L., Liu, H., Voss, C.K., Wu, C., Schiller, M.R., and Li, S.S.C. (2010). Loops govern SH2 domain specificity by controlling access to binding pockets. Sci. Signal. *3*, ra34.

Keiffer, T.R., Ciancanelli, M.J., Edwards, M.R., and Basler, C.F. (2020). Interactions of the Nipah Virus P, V, and W proteins across the STAT family of transcription factors. mSphere *5*, e00449-20.

Koksal, A.C., Nardozzi, J.D., and Cingolani, G. (2009). Dimeric quaternary structure of the prototypical dual specificity phosphatase VH1. J. Biol. Chem. *284*, 10129–10137.

Ladbury, J.E., and Arold, S.T. (2011). Energetics of Src homology domain interactions in receptor tyrosine kinase-mediated signaling. Methods Enzymol *488*, 147–183, Academic Press.

Lasso, G., Honig, B., and Shapira, S.D. (2021). A sweep of earth's virome reveals host-guided viral protein structural mimicry and points to determinants of human disease. Cell Syst *12*, 82–91.e3.

Leung, S., Qureshi, S.A., Kerr, I.M., Darnell, J.E., and Stark, G.R. (1995). Role of STAT2 in the alpha interferon signaling pathway. Mol. Cell. Biol. *15*, 1312–1317.

Li, X., Leung, S., Kerr, I.M., and Stark, G.R. (1997). Functional subdomains of STAT2 required for preassociation with the alpha interferon receptor and for signaling. Mol. Cell. Biol. *17*, 2048–2056.

Liebschner, D., Afonine, P.V., Baker, M.L., Bunkóczi, G., Chen, V.B., Croll, T.I., Hintze, B., Hung, L.W., Jain, S., McCoy, A.J., et al. (2019). Macromolecular structure determination using X-rays, neutrons and electrons: recent developments in Phenix. Acta Crystallogr. D Struct. Biol. *75*, 861–877.

Liu, R., Americo, J.L., Cotter, C.A., Earl, P.L., Erez, N., Peng, C., and Moss, B. (2021). One or two injections of MVA-vectored vaccine shields hACE2 transgenic mice from SARS-CoV-2 upper and lower respiratory tract infection. Proc. Natl. Acad. Sci. USA *118*, e2026785118.

Lu, Y., Stuart, J.H., Talbot-Cooper, C., Agrawal-Singh, S., Huntly, B., Smid, A.I., Snowden, J.S., Dupont, L., and Smith, G.L. (2019). Histone deacetylase 4 promotes type I interferon signaling, restricts DNA viruses, and is degraded via vaccinia virus protein C6. Proc. Natl. Acad. Sci. USA *116*, 11997–12006.

Ludlow, L.E., Lo, M.K., Rodriguez, J.J., Rota, P.A., and Horvath, C.M. (2008). Henipavirus V protein association with polo-like kinase reveals functional overlap with STAT1 binding and interferon evasion. J. Virol. *82*, 6259–6271.

Maluquer de Motes, C.M., Cooray, S., Ren, H., Almeida, G.M.F., McGourty, K., Bahar, M.W., Stuart, D.I., Grimes, J.M., Graham, S.C., and Smith, G.L. (2011). Inhibition of apoptosis and NF-κB activation by vaccinia protein N1 occur via distinct binding surfaces and make different contributions to virulence. PLoS Pathog. *7*, e1002430.

Maluquer de Motes, C.M., Schiffner, T., Sumner, R.P., and Smith, G.L. (2014). Vaccinia virus virulence factor N1 can be ubiquitylated on multiple lysine residues. J. Gen. Virol. *95*, 2038–2049.

Mann, B.A., Huang, J.H., Li, P., Chang, H.C., Slee, R.B., O'Sullivan, A., Mathur, A., Yeh, N., Klemsz, M.J., Brutkiewicz, R.R., et al. (2008). Vaccinia virus blocks Stat1-dependent and Stat1-independent gene expression induced by type I and type II interferons. J. Interferon Cytokine Res. *28*, 367–380.

Mao, X., Ren, Z., Parker, G.N., Sondermann, H., Pastorello, M.A., Wang, W., McMurray, J.S., Demeler, B., Darnell, J.E., and Chen, X. (2005). Structural bases of unphosphorylated STAT1 association and receptor binding. Mol. Cell *17*, 761–771.

McWilliam, H., Li, W., Uludag, M., Squizzato, S., Park, Y.M., Buso, N., Cowley, A.P., and Lopez, R. (2013). Analysis Tool web services from the EMBL-EBI. Nucleic Acids Res. *41*, W597–W600.

Mendoza, J.L., Escalante, N.K., Jude, K.M., Sotolongo Bellon, J., Su, L., Horton, T.M., Tsutsumi, N., Berardinelli, S.J., Haltiwanger, R.S., Piehler, J., et al. (2019). Structure of the IFNγ receptor complex guides design of biased agonists. Nature *567*, 56–60.

Mishra, P.M., Verma, N.C., Rao, C., Uversky, V.N., and Nandi, C.K. (2020). Intrinsically disordered proteins of viruses: involvement in the mechanism of cell regulation and pathogenesis. Prog. Mol. Biol. Transl. Sci. (Elsevier B.V.) *174*, 1–78.

Montanuy, I., Alejo, A., and Alcami, A. (2011). Glycosaminoglycans mediate retention of the poxvirus type I interferon binding protein at the cell surface to locally block interferon antiviral responses. FASEB J *25*, 1960–1971.

Mossman, K., Upton, C., Buller, R.M.L., and McFadden, G. (1995). Species specificity of ectromelia virus and vaccinia virus interferon-γ binding proteins. Virology *208*, 762–769.

Mühlemann, B., Vinner, L., Margaryan, A., Wilhelmson, H., de la Fuente Castro, C., Allentoft, M.E., de Barros Damgaard, P., Hansen, A.J., Holtsmark Nielsen, S., Strand, L.M., et al. (2020). Diverse variola virus (smallpox) strains were widespread in northern Europe in the Viking Age. Science *369*, eaaw8977.

Nagano, Y., Sugiyama, A., Kimoto, M., Wakahara, T., Noguchi, Y., Jiang, X., Saijo, S., Shimizu, N., Yabuno, N., Yao, M., et al. (2020). The measles virus V protein Binding Site to STAT2 overlaps that of IRF9. J. Virol. *94*.

Najarro, P., Traktman, P., and Lewis, J.A. (2001). Vaccinia virus blocks gamma interferon signal transduction: viral VH1 phosphatase reverses Stat1 activation. J. Virol. *75*, 3185–3196.

Nguyen, K.B., Watford, W.T., Salomon, R., Hofmann, S.R., Pien, G.C., Morinobu, A., Gadina, M., O'Shea, J.J., and Biron, C.A. (2002). Critical role for STAT4 activation by type 1 interferons in the interferon-γ response to viral infection. Science *297*, 2063–2066.

Oda, K., Matoba, Y., Irie, T., Kawabata, R., Fukushi, M., Sugiyama, M., and Sakaguchi, T. (2015). Structural basis of the inhibition of STAT1 activity by Sendai virus C protein. J. Virol. *89*, 11487–11499.

Pellegrini, S., John, J., Shearer, M., Kerr, I.M., and Stark, G.R. (1989). Use of a selectable marker regulated by alpha interferon to obtain mutations in the signaling pathway. Mol. Cell. Biol. *9*, 4605–4612.

Peters, N.E., Ferguson, B.J., Mazzon, M., Fahy, A.S., Krysztofinska, E., Arribas-Bosacoma, R., Pearl, L.H., Ren, H., and Smith, G.L. (2013). A mechanism for the inhibition of DNA-PK-mediated DNA sensing by a virus. PLoS Pathog *9*, e1003649.

Qureshi, S.A., Leung, S., Kerr, I.M., Stark, G.R., and Darnell, J.E. (1996). Function of Stat2 protein in transcriptional activation by alpha interferon. Mol. Cell. Biol. *16*, 288–293.

Randall, R.E., and Goodbourn, S. (2008). Interferons and viruses: an interplay between induction, signalling, antiviral responses and virus countermeasures. J. Gen. Virol. *89*, 1–47.

Rengachari, S., Groiss, S., Devos, J.M., Caron, E., Grandvaux, N., and Panne, D. (2018). Structural basis of STAT2 recognition by IRF9 reveals molecular insights into ISGF3 function. Proc. Natl. Acad. Sci. U S A *115*, E601–E609.

Robert, X., and Gouet, P. (2014). Deciphering key features in protein structures with the new ENDscript server. Nucleic Acids Res. *42*, W320–W324.

Rodriguez, J.J., Cruz, C.D., and Horvath, C.M. (2004). Identification of the nuclear export signal and STAT-binding domains of the Nipah virus V protein reveals mechanisms underlying interferon evasion. J. Virol. *78*, 5358–5367.

Rodriguez, J.J., Parisien, J.-P., and Horvath, C.M. (2002). Nipah virus V protein evades alpha and gamma interferons by preventing STAT1 and STAT2 activation and nuclear accumulation. J. Virol. *76*, 11476–11483.

Satterfield, B.A., Borisevich, V., Foster, S.L., Rodriguez, S.E., Cross, R.W., Fenton, K.A., Agans, K.N., Basler, C.F., Geisbert, T.W., and Mire, C.E. (2019). Antagonism of STAT1 by Nipah virus P gene products modulates disease course but not lethal outcome in the ferret model. Sci. Rep. *9*, 16710.

Schindelin, J., Arganda-Carreras, I., Frise, E., Kaynig, V., Longair, M., Pietzsch, T., Preibisch, S., Rueden, C., Saalfeld, S., Schmid, B., et al. (2012). Fiji: an open-source platform for biological-image analysis. Nat. Methods *9*, 676–682.

Schmidt, F.I., Bleck, C.K.E., Reh, L., Novy, K., Wollscheid, B., Helenius, A., Stahlberg, H., and Mercer, J. (2013). Vaccinia virus entry is followed by core activation and proteasome-mediated release of the immunomodulatory effector VH1 from lateral bodies. Cell Rep. *4*, 464–476.

Schneider, W.M., Chevillotte, M.D., and Rice, C.M. (2014). Interferon-stimulated genes: A complex web of host defenses. Annu. Rev. Immunol. *32*, 513–545.

Shaw, M.L., García-Sastre, A., Palese, P., and Basler, C.F. (2004). Nipah virus V and W proteins have a common STAT1-binding domain yet inhibit STAT1 activation from the cytoplasmic and nuclear compartments, respectively. J. Virol. *78*, 5633–5641.

Shemesh, M., Lochte, S., Piehler, J., and Schreiber, G. (2021). IFNAR1 and IFNAR2 play distinct roles in initiating type I interferon–induced JAK-STAT signaling and activating STATs. Sci. Signal. *14*, eabe4627.

Smart, O.S., Womack, T.O., Flensburg, C., Keller, P., Paciorek, W., Sharff, A., Vonrhein, C., and Bricogne, G. (2012). Exploiting structure similarity in refinement: automated NCS and target-structure restraints in BUSTER. Acta Crystallogr. D Biol. Crystallogr. *68*, 368–380.

Smith, G.L., Benfield, C.T.O., Maluquer de Motes, C., Mazzon, M., Ember, S.W.J., Ferguson, B.J., and Sumner, R.P. (2013). Vaccinia virus immune evasion: mechanisms, virulence and immunogenicity. J. Gen. Virol. *94*, 2367–2392.

Smith, G.L., Talbot-Cooper, C., and Lu, Y. (2018). How does vaccinia virus interfere With interferon? Adv. Virus Res. *100*, 355–378, Academic Press.

Soday, L., Lu, Y., Albarnaz, J.D., Davies, C.T.R., Antrobus, R., Smith, G.L., and Weekes, M.P. (2019). Quantitative temporal proteomic analysis of vaccinia virus infection reveals regulation of histone deacetylases by an interferon antagonist. Cell Rep *27*, 1920–1933.e7.

Stuart, J.H., Sumner, R.P., Lu, Y., Snowden, J.S., and Smith, G.L. (2016). Vaccinia virus protein C6 inhibits Type I IFN signalling in the nucleus and binds to the transactivation domain of STAT2. PLoS Pathog. *12*, e1005955.

Symons, J.A., Alcamí, A., and Smith, G.L. (1995). Vaccinia virus encodes a soluble type I interferon receptor of novel structure and broad species specificity. Cell *81*, 551–560.

Teo, H., Perisic, O., González, B., and Williams, R.L. (2004). ESCRT-II, an endosome-associated complex required for protein sorting: crystal structure and interactions with ESCRT-III and membranes. Dev. Cell *7*, 559–569.

Torpey, N., Maher, S.E., Bothwell, A.L.M., and Pober, J.S. (2004). Interferon α but not interleukin 12 activates STAT4 signaling in human vascular endothelial cells. J. Biol. Chem. *279*, 26789–26796.

Torres, A.A., Macilwee, S.L., Rashid, A., Cox, S.E., Albarnaz, J.D., Bonjardim, C.A., and Smith, G.L. (2020). Spir-1 promotes IRF3 activation and is targeted by vaccinia virus protein K7, Preprint at: bioRxiv, 2020.08.31.276659

Unterholzner, L., Sumner, R.P., Baran, M., Ren, H., Mansur, D.S., Bourke, N.M., Randow, F., Smith, G.L., and Bowie, A.G. (2011). Vaccinia virus protein C6 is a virulence factor that binds TBK-1 adaptor proteins and inhibits activation of IRF3 and IRF7. PLoS Pathog. *7*, e1002247.

Vonrhein, C., Flensburg, C., Keller, P., Sharff, A., Smart, O., Paciorek, W., Womack, T., and Bricogne, G. (2011). Data processing and analysis with the autoPROC toolbox. Acta Crystallogr. D Biol. Crystallogr. *67*, 293–302.

Wang, B., Thurmond, S., Zhou, K., Sánchez-Aparicio, M.T., Fang, J., Lu, J., Gao, L., Ren, W., Cui, Y., Veit, E.C., et al. (2020). Structural basis for STAT2 suppression by Flavivirus NS5. Nat. Struct. Mol. Biol. *27*, 875–885.

Wang, Y., Song, Q., Huang, W., Lin, Y., Wang, X., Wang, C., Willard, B., Zhao, C., Nan, J., Holvey-Bates, E., et al. (2021). A virus-induced conformational switch of STAT1-STAT2 dimers boosts antiviral defenses. Cell Res. *31*, 206–218.

Weaver, J.R., Shamim, M., Alexander, E., Davies, D.H., Felgner, P.L., and Isaacs, S.N. (2007). The identification and characterization of a monoclonal antibody to the vaccinia virus E3 protein. Virus Res. *130*, 269–274.

Wennier, S.T., Brinkmann, K., Steinhäußer, C., Mayländer, N., Mnich, C., Wielert, U., Dirmeier, U., Hausmann, J., Chaplin, P., and Steigerwald, R. (2013). A novel naturally occurring tandem promoter in modified vaccinia virus Ankara drives very early gene expression and potent immune responses. PLoS One *8*, e73511.

Wenta, N., Strauss, H., Meyer, S., and Vinkemeier, U. (2008). Tyrosine phosphorylation regulates the partitioning of STAT1 between different dimer conformations. Proc. Natl. Acad. Sci. USA *105*, 9238–9243.

Xue, B., Blocquel, D., Habchi, J., Uversky, A.V., Kurgan, L., Uversky, V.N., and Longhi, S. (2014). Structural disorder in viral proteins. Chem. Rev. *114*, 6880–6911.

Yan, H., Krishnan, K., Greenlund, A.C., Gupta, S., Lim, J.T.E., Schreiber, R.D., Schindler, C.W., and Krolewski, J.J. (1996). Phosphorylated interferon-α receptor 1 subunit (IFNaR1) acts as a docking site for the latent form of the 113 kDa STAT2 protein. EMBO J. *15*, 1064–1074.

Yang, C., Mai, H., Peng, J., Zhou, B., Hou, J., and Jiang, D. (2020). STAT4: an immunoregulator contributing to diverse human diseases. Int. J. Biol. Sci. *16*, 1575–1585.

Yang, Z., Cao, S., Martens, C.A., Porcella, S.F., Xie, Z., Ma, M., Shen, B., and Moss, B. (2015). Deciphering poxvirus gene expression by RNA sequencing and ribosome profiling. J. Virol. *89*, 6874–6886.

Zhao, W., Lee, C., Piganis, R., Plumlee, C., de Weerd, N., Hertzog, P.J., and Schindler, C. (2008). A conserved IFN-α receptor tyrosine motif directs the biological response to Type I IFNs. J. Immunol. *180*, 5483–5489.

STAR★METHODS

KEY RESOURCES TABLE

REAGENT or RESOURCE	SOURCE	IDENTIFIER
Antibodies		
Rabbit anti-STAT1	Cell Signaling Technologies	14994 RRID:AB_2737027
Rabbit anti-STAT2	Cell Signaling Technologies	72604 RRID:AB_2799824
Rabbit anti-IRF9	Cell Signaling Technologies	76684 RRID:AB_2799885
Rabbit anti-pSTAT1 (Tyr 701)	Cell Signaling Technologies	9167 RRID:AB_561284
Rabbit anti-pSTAT2 (Tyr 690)	Cell Signaling Technologies	88410 RRID:AB_2800123
Rabbit anti-IRF1	Cell Signaling Technologies	8478 RRID:AB_10949108
Mouse anti-IFIT3	Santa Cruz	sc-393512 RRID:AB_2857847
Mouse anti-IFITM1-3	Santa Cruz	sc-374026 RRID:AB_10916884
Rabbit anti-C6	Laboratory of Geoffrey L Smith (Unterholzner et al., 2011)	N/A
Mouse anti-E3	(Weaver et al., 2007)	MAb 2015B2
Mouse anti-GAPDH	Sigma-Aldrich	G8795 RRID:AB_1078991
Rabbit anti-actin	Sigma-Aldrich	A2066 RRID:AB_476693
Mouse anti-FLAG	Sigma-Aldrich	F3165; RRID:AB_259529
Rabbit anti-FLAG	Sigma-Aldrich	F7425 RRID: AB_439687
Mouse anti-HA	Biolegend	901513; RRID:AB_2565335
Rabbit anti-V5	Cell Signaling Technologies	13202; RRID:AB_2687461
Mouse anti-pTyr	Santa Cruz	sc-7020 RRID:AB_628123
Biotin-SP (long spacer) Affinipure goat anti-mouse IgG, light chain specific	Jackson ImmunoResearch Labs	115-065-174 RRID: AB_2338570
IRDye 680RD-conjugated goat anti-rabbit IgG	LI-COR	926-68071; RRID:AB_10956166
IRDye 680LT-conjugated goat anti-mouse IgG	LI-COR	926-68020; RRID:AB_10706161
IRDye 800CW-conjugated goat anti-rabbit IgG	LI-COR	926-32211; RRID:AB_621843
IRDye 800CW-conjugated goat anti-mouse IgG	LI-COR	926-32210; RRID:AB_621842
IRDye 800CW-conjugated Streptavidin	LI-COR	926-32230
Donkey anti-Mouse IgG (H+L) secondary antibody, Alexa Fluor 546	Molecular Probes	A10036; RRID:AB_2534012
Goat anti-Rabbit IgG (H+L) secondary antibody, Alexa Fluor 488	Molecular Probes	A11008; RRID:AB_143165
Goat anti-Rabbit IgG (H+L) secondary antibody, Alexa Fluor 546	Molecular Probes	A11010 RRID:AB_2434077
Donkey anti-Mouse IgG (H+L) highly cross-adsorbed secondary antibody, Alexa Fluor Plus 488	Thermo Fisher Scientific	A32766 RRID:AB_2762823
Bacterial and virus strains		
T7 Express competent *E. coli*	New England Biolabs	C2566I
E. coli (subcloning efficiency DH5α competent Cells)	Invitrogen	18265-017

(*Continued on next page*)

Continued

REAGENT or RESOURCE	SOURCE	IDENTIFIER
VACV strain Western Reserve: v018	This paper	N/A
VACV strain Western Reserve: vΔ018	This paper	N/A
VACV strain Western Reserve: vTAP-018	This paper	N/A
VACV strain Western Reserve: vTAP-N1	Laboratory of Geoffrey L Smith (Maluquer de Motes et al., 2014)	N/A
VACV strain Western Reserve: vFLAG-A36	Laboratory of Geoffrey L Smith (unpublished, constructed by Dr David C.J.Carpentier)	N/A
Sendai virus (SeV) strain Cantell	A gift from Steve Goodbourn (St George's Hospital Medical School, University of London) Licence No. ITIMP17.0612A	N/A
**Chemicals, peptides, and recombinant proteins**		
Fluor-pIFNGR1 12-mer peptide (5Flu-GTSFGpYDKPHVLV-NH2)	PeptideSynthetics (UK)	https://www.peptidesynthetics.co.uk/
pIFNGR1 5-mer peptide (Ac-pYDKPH-NH2)	Genosphere Biotechnologies	https://www.genosphere-biotech.com/
018 21-mer peptide (Ac-MWSVFIHGHD GSNKGSKTYTS-NH2)	Genosphere Biotechnologies	https://www.genosphere-biotech.com/
018 phospho 21-mer peptide (Ac-MWSVFIHGHDGSNKGSKT(pY)TS-NH2)	Genosphere Biotechnologies	https://www.genosphere-biotech.com/
Full-length STAT1 protein	This paper	N/A
GB1-018 protein fusions	This paper	N/A
GB1-NiV-V protein fusions	This paper	N/A
$STAT1^{132-684,\Delta 183-190,H182A,E393A,E394A}$ protein	This paper	N/A
Dulbecco's modified Eagle's Medium (DMEM)	Gibco	41966-029
Minimal essential medium (MEM)	Gibco	31095-029
MEM non-essential amino acids (NEAAs)	Gibco	11140050
Trypsin-EDTA	Gibco	25300-054
Penicillin-streptomycin	Gibco	15140-122
Fetal bovine serum (FBS)	PAN-Biotech	P30-19375
Opti-MEM I reduced serum medium	Gibco	51985-026
Bovine serum albumin (BSA)	Sigma-Aldrich	A3059
Blasticidin S HCl solution	Santa Cruz	sc-495389
Zeocin	InvivoGen	ant-zn-1
Xanthine sodium salt	Sigma-Aldirch	x3627
Hypoxanthine	Sigma-Aldrich	H9377
Mycophenolic acid	Sigma-Aldrich	M5255
Agarose (low gelling temperature)	Sigma-Aldrich	A4018
Doxycycline hydrochloride	Melford	D43020
cOmplete, EDTA-free protease inhibitor cocktail	Roche	11836153001
PhosSTOP phosphatase inhibitor cocktail	Roche	04906837001
16% Paraformaldehyde aqueous solution, EM grade	Electron Microscopy Sciences	15710
IFNa 2 human	Sigma-Aldrich	SRP4594
IFNγ human	PreproTech	300-02
DNase I	Sigma-Aldrich	DN25
DAPI (4',6-diamidino-2-phenylindole)	Biotium	40043
Mowiol 4-88	Calbiochem	475904
Polyethylenimine (PEI), linear, MW 25000	Polysciences	23966
Passive lysis 5X buffer	Promega	E1941

(*Continued on next page*)

Continued

REAGENT or RESOURCE	SOURCE	IDENTIFIER
Acetyl coenzyme A (firefly luciferase reagents)	Nanolight Technology	315-500
Luciferin (firefly luciferase reagent)	Nanolight Technology	306-500
Coelenterazine (*Renilla* luciferase reagent)	Nanolight Technology	303-10
Hanks' balanced salt solution (HBSS)	Lonza	10-527F
Collagenase (type I)	Worthington Biochemicals	LS004216
Crystal violet	Sigma-Aldrich	C0775
Formaldehyde	Sigma-Aldrich	252549
Gentamicin (50 mg/mL)	Sigma-Aldrich	G1397
Carboxymethylcellulose sodium salt	Sigma-Aldrich	419273
TCEP	Melford	T26500
Tobacco etch virus (TEV) protease	Prepared in-house from the pRK793 expression plasmid (Addgene #8827)	N/A
AEBSF	Melford	A20010
polyethylene glycol (PEG) 3350	Sigma-Aldrich	202444
Ni-NTA agarose	Cube Biotech	31103
**Critical commercial assays**		
Pierce BCA protein assay kit	Thermo Fisher Scientific	23227
MycoAlert mycoplasma detection kit	Lonza	LT07-218
Q5 High-fidelity DNA polymerase	New England Biolabs	M0491
Q5 Site-directed mutagenesis kit	New England Biolabs	E0554
One*Taq* Quick-load 2X master mix with standard buffer	New England Biolabs	M486
T4 DNA ligase	New England Biolabs	M0202
*Trans*IT-LT1	Mirus Bio	MIR 2305
Monarch Total RNA miniprep kit	New England Biolabs	T2010
Luna Universal one-step RT-qPCR kit	New England Biolabs	E3005
Strep-TactinXT superflow resin	IBA	2-4030-002
Anti-FLAG M2 affinity gel	Sigma-Alrich	A220
TnT coupled wheat germ extract (SP6)	Promega	L4130
Lysing matric S (1/8'') metal beads	MPBio	116925100
Qiagen RNeasy mini kit	Qiagen	74104
RT^2 First stand kit	Qiagen	330404
Antiviral response qPCR array	Qiagen	PAMM-122Z-24
RT^2 SYBR Green ROX qPCR mastermix	Qiagen	330523
RT^2 qPCR primer assay for mouse IRF1	Qiagen	PPM03203D-200
RT^2 qPCR primer assay for mouse IFNγ	Qiagen	PPM03121A-200
RT^2 qPCR primer assay for mouse Actb	Qiagen	PPM02945B-200
RT^2 qPCR primer assay for mouse B2M	Qiagen	PPM03562A-200
RT^2 qPCR primer assay for mouse GAPDH	Qiagen	PPM02946E-200
**Deposited data**		
Structure of 018 complexed with STAT1 core fragment	This paper	PDB ID: 7nuf
**Experimental models: Cell lines**		
BS-C-1	ATCC	CCL-26
CV-1	ATCC	CCL-70
RK13	ATCC	CCL-37
Mouse embryo fibroblast (MEF)	A gift from Prof. Dr Eugen Kerkhoff – University Hospital Regenburg, Germany	N/A

(*Continued on next page*)

Continued

REAGENT or RESOURCE	SOURCE	IDENTIFIER
HEK 293T	ATCC	CRL-11268
HeLa	ATCC	CCL-2
T-REx 293	Life Technologies	R71007
T-REx 293 EV	This paper	N/A
T-REx 293TAP-N1	This paper	N/A
T-REx 293TAP-018	This paper	N/A
T-REx 293 TAP-NiV-V	This paper	N/A
A549	ATCC	CCL-185
2fTGH	Sigma Aldrich	12021508
U3A	Sigma-Aldrich	12021503
U6A	Sigma Aldrich	12021507
TK$^-$ 143B	ATCC	CRL-8303
Experimental models: Organisms/strains		
BALB/c mice, female, adult aged 6-10 weeks old	Taconic Farms	Mouse strain: BALB/CANNTAC
Oligonucleotides		
Primers for construction of recombinant DNA	See Table S1	N/A
Primers for RT-qPCR (cell culture)	See Table S2	N/A
Primers for analytical PCR or sequencing	See Table S3	N/A
Recombinant DNA		
pcDNA4/TO	Thermo Fisher Scientific	V102020
pcDNA4/TO TAP-018	This Paper	N/A
pcDNA/TO TAP-NiV-V	This Paper	N/A
pcDNA/TO TAP-N1	Laboratory of Geoffrey L Smith (Maluquer de Motes et al., 2011)	N/A
pcDNA4/TO TAP-C6	Laboratory of Geoffrey L Smith (Stuart et al., 2016)	N/A
pcDNA4/TO TAP-C16	Laboratory of Geoffrey L Smtih (Peters et al., 2013)	N/A
pcDNA4/TO TAP-018 (1-54)	This Paper	N/A
pcDNA4/TO TAP-018 (1-48)	This Paper	N/A
pcDNA4/TO TAP-018 (1-43)	This Paper	N/A
pcDNA4/TO TAP-018 (1-35)	This Paper	N/A
pcDNA4/TO TAP-018 (1-30)	This Paper	N/A
pcDNA4/TO TAP-018 (1-27)	This Paper	N/A
pcDNA4/TO TAP-018(1-24)	This Paper	N/A
pcDNA4/TO TAP-018 (1-21)	This Paper	N/A
pcDNA4/TO TAP-018 (8-60)	This Paper	N/A
pcDNA4/TO TAP-018 (11-60)	This Paper	N/A
pcDNA4/TO TAP-018 (14-60)	This Paper	N/A
pcDNA4/TO TAP-018 (17-60)	This Paper	N/A
pcDNA4/TO TAP-018 (22-60)	This Paper	N/A
pcDNA4/TO TAP-018 (31-60)	This Paper	N/A
pcDNA4/TO TAP-018^{AGA}	This Paper	N/A
pcDNA3 HA-C6	Laboratory of Geoffrey L Smith (Unterholzner et al., 2011)	N/A
pcDNA3 HA-018	This Paper	N/A
pcDNA3 HA-018^{AGA}	This Paper	N/A
pcDNA3 V5-STAT1	This Paper	N/A
pcDNA3 V5-STAT2	This Paper	N/A

(*Continued on next page*)

CellPress
OPEN ACCESS

Continued

REAGENT or RESOURCE	SOURCE	IDENTIFIER
pcDNA3 V5-STAT3 (human)	This Paper	N/A
pcDNA3 V5-STAT3 (mouse)	This Paper	N/A
pcDNA3 V5-STAT4	This Paper	N/A
pcDNA3 V5-STAT5A	This Paper	N/A
pcDNA3 V5-STAT5B	This Paper	N/A
pcDNA3 V5-STAT6	This Paper	N/A
pcDNA3 V5-STAT1 (1-712)	This Paper	N/A
pcDNA3 V5-STAT1 (1-684)	This Paper	N/A
pcDNA3 V5-Fus1	This Paper	N/A
pcDNA3 V5-Fus2	This Paper	N/A
pcDNA3 V5-STAT3^{Q635H} (human)	This Paper	N/A
13F	A gift from Curt Horvath (Northwestern University, USA)	N/A
31F	A gift from Curt Horvath (Northwestern University, USA)	N/A
ISRE-Luc	Promega	E4141
GAS-Luc	A gift from Andrew Bowie (Trinity College Dublin, Republic of Ireland)	N/A
IFNβ-Luc	A gift from T. Taniguchi (University of Tokyo, Japan)	N/A
TK-Renilla-Luc	Promega (GL3-*Renilla* vector was made by replacing the firefly luciferase ORF from pGL3-control (Promega) with the *Renilla* luciferase ORF from pRL-TK (Promega)	E2241
pF3A	Promega	L5671
pF3A TAP-018	This Paper	N/A
pF3A STAT1	This Paper	N/A
pF3A FLAG-K7	Laboratory of Geoffrey L Smith (Torres et al., 2020)	N/A
pUC13-Ecogpt-EGFP Δ018	This Paper	N/A
pUC13-Ecogpt-EGFP TAP-018	This Paper	N/A
pOPTH	(Teo et al., 2004)	N/A
pOPTH-TEV	This Paper	N/A
pOPTH-TEV-STAT1$^{132\text{-}684,\Delta 183\text{-}190,H182A,E393A,E394A}$	This Paper	N/A
pEXP-nHis	Laboratory of Marko Hyvonen	Addgene #112558
pEXP-nHis-STAT1	This Paper	N/A
pPEPT1	Laboratory of Marko Hyvonen Vector map is provided in Figure S8	N/A
pPEPT 018	This Paper	N/A
pPEPT 018^{T2}	This Paper	N/A
pPEPT 018^{T3}	This Paper	N/A
pPEPT 018^{AGA}	This Paper	N/A
pPEPT NiV-V (110-140)	This Paper	N/A
pPEPT NiV-V (110-140)^{ADA}	This Paper	N/A
**Software and algorithms**		
Image Studio Lite Quantification Software	LI-COR Biosciences	https://www.licor.com/bio/image-studio-lite/
MARS Data Analysis Software	BMG LABTECH	https://www.bmglabtech.com/mars-data-analysis-software/

(*Continued on next page*)

Continued

REAGENT or RESOURCE	SOURCE	IDENTIFIER
ImageJ-Fiji	(Schindelin et al., 2012)	https://imagej.net/software/fiji/
QuantStudio Software	Applied Biosystems	https://www.thermofisher.com/uk/en/home/technical-resources/software-downloads/applied-biosystems-viia-7-real-time-pcr-system.html
Origin for ITC200	Malvern Instruments	https://www.malvernpanalytical.com/en
autoProc	(Vonrhein et al., 2011)	https://www.globalphasing.com/autoproc/
Phenix.refine	(Liebschner et al., 2019)	https://www.phenix-online.org/
autoBuster	(Smart et al., 2012)	https://www.globalphasing.com/buster/
Prism	GraphPad	https://www.graphpad.com/scientific-software/prism/
GeneGlobe Data Analysis Centre	Qiagen	https://geneglobe.qiagen.com/us/analyze
Coot	(Emsley et al., 2010)	https://www2.mrc-lmb.cam.ac.uk/personal/pemsley/coot/
ChimeraX	UCSF	https://www.rbvi.ucsf.edu/chimerax/
ASTRA	Wyatt Technology	https://www.wyatt.com/
Uniprot	(Bateman, 2019)	https://www.uniprot.org/
NCBI blast	(Johnson et al., 2008)	https://blast.ncbi.nlm.nih.gov/Blast.cgi
Clustal Omega	(McWilliam et al., 2013)	https://www.ebi.ac.uk/Tools/msa/clustalo/
ESPrit 3.0	(Robert and Gouet, 2014)	https://espript.ibcp.fr

RESOURCE AVAILABILITY

Lead contact

Further information and requests for resources and reagents should be directed to and will be fulfilled by the lead contact, Geoffrey L Smith (gls37@cam.ac.uk).

Materials availability

See above.

Data and code availability

018:STAT1 X-ray crystallographic structure has been deposited on the PDB under the accession code 7nuf.

All raw data relating to this manuscript can be found at doi: 10.17632/s6zzv4nd3s.1

EXPERIMENTAL MODEL AND SUBJECT DETAILS

Cell lines

BS-C-1 (ATCC), CV-1 (ATCC), MEFs (A gift from Prof. Dr Eugen Kerkhoff), HEK 293T (ATCC), A549 (ATCC), 2fTGH (Sigma-Aldrich), U3A (a 2fTGH derived STAT1$^{-/-}$ cell line, Sigma-Aldrich), U6A (a 2fTGH derived, STAT2$^{-/-}$ cell line, Sigma-Aldrich), and TK^{-} 143B cells (ATCC) were maintained in DMEM (Gibco) supplemented with 10% (v/v) foetal bovine serum (FBS, PAN-Biotech) and penicillin-streptomycin (PS, 50 μg/mL, Gibco). T-REx 293 cells (Life technologies) were maintained in DMEM supplemented with 10% (v/v) FBS, PS (50 μg/mL) and blasticidin (10 μg/mL, Santa Cruz), and T-REx 293 derived cells lines expressing EV, TAP-N1, TAP-018, or TAP-NiV-V were further supplemented with zeocin (100 ug/mL, Invivogen). HeLa (ATCC) and RK13 cells (ATCC) were maintained in MEM (Gibco) supplemented with 10% (v/v) FBS, PS (50 μg/mL) and 1% (v/v) 100 X non-essential amino acids (Gibco). The construction of T-REx 293 cell lines expressing proteins inducibly is outlined in the method details section.

Viruses

Recombinant VACV vTAP-N1 was derived from VACV strain WR (VACV-WR, GenBank: AY243312.1) (Maluquer de Motes et al., 2014). v018, vΔ018 and vTAP-018 described in this paper were all derived from VACV strain WR and their construction is outlined in the method details section.

Animals

Specific pathogen-free BALB/c mice were obtained from Taconic Farms. 6-10 weeks old female mice were used. Mice were housed under specific pathogen-free conditions (including negativity for murine norovirus, mouse parvovirus, and mouse hepatitis virus) and

were maintained on standard rodent chow and water supplied *ad libitum*. All animal studies were approved by and performed in accordance with the Animal Care and Use Committee of the National Institute of Allergy and Infectious Diseases (NIAID), USA.

METHOD DETAILS

Orthologue alignments

Identifiers for poxvirus genomes from which the amino acids sequences of 018 orthologues were derived: vaccinia strain Western Reserve (VACV-WR, GenBank: AY243312.1), Modified vaccinia Ankara (MVA, GenBank: AY603355.1), variola virus (VARV, GenBank: X69198.1), monkeypox virus (MPXV, GenBank: AF380138.1), cowpox virus strain Brighton Red (CPXV-BR, GenBank: AF482758.2), ectromelia virus (ECTV, GenBank: AF012825.2), camelpox virus (CMLV, GenBank: AF438165.1), rabbitpox virus (RPXV, GenBank: AY484669.1), raccoonpox virus (RCNV, GenBank: KP143769.1), skunkpox virus (SKPTV, GenBank: KU749310.1), taterapox virus (TATV, GenBank: DQ437594.1), Cotia virus (COTV, GenBank: HQ647181.2), Yoka poxvirus (YKPV, GenBank: HQ849551.1). Alignments were performed using Clustal Omega (McWilliam et al., 2013) and conservation annotation was performed using ESPirt 3.0 (Robert and Gouet, 2014).

Plasmids

The 018 open reading frame (ORF) was codon-optimised for expression in human cells and was synthesised by Gene Art (Thermo Fisher Scientific). All plasmids are described in recombinant DNA key resource table and primers used for construction in (Table S1). The pPEPT1 vector map is provided in Figure S8.

Construction of T-REx 293 cell lines expressing proteins inducibly

T-REx 293 were transfected with pcDNA4/TO empty vector (EV) or expression plasmids (pcDNA4/TO TAP-018, pcDNA4/TO TAP-N1 and pcDNA4/TO TAP-NiV-V) using Transit-LT1 (Mirus Bio). Prior to transfection, pcDNA4/TO plasmids were linearised using *Pvu*I (NEB) to decrease the potential for plasmid-chromosomal integration within the viral ORF. Cells with integrated plasmid were selected in the presence of blasticidin (10 μg/mL) and zeocin (100 μg/mL) and single clones were obtained by limiting dilution. Clones were amplified and lysates were analysed for the expression of TAP-tagged protein by immunoblotting.

The expression of TAP-tagged proteins from T-REx 293-derived cells was induced by addition of doxycycline (100 ng/mL, Santa Cruz) to the medium for 24 h.

Construction of recombinant VACVs

Recombinant VACVs (vΔ018 and vTAP-018) were constructed using transient dominant selection (Falkner and Moss, 1990). To construct the pUC13-Ecogpt-EGFP-Δ018 plasmid to remove the entire 018 ORF, the downstream (301 bp) and upstream (300 bp) flanking regions of the 018 ORF were amplified by PCR from purified VACV (strain WR) DNA. A 15-bp complementary sequence was added to the internal upstream and downstream primers to enable joining of the two-flanking regions by overlapping PCR. The resulting PCR product was ligated into pUC13-Ecogpt-EGFP using *Pst*I (NEB) and *Bam*HI (NEB) cloning sites. To construct the pUC13-Ecogpt-EGFP TAP-018 plasmid, the downstream flanking and 018 ORF (484 bp) and the upstream region of the ORF (300 bp) were amplified separately by PCR from purified VACV (strain WR) DNA. The 018 ORF plus downstream flanking region PCR product was ligated into pcDNA4/TO vector containing an N-terminal TAP-tag using *Not*I (NEB) and *Xba*I (NEB) as an intermediate cloning step. The N-terminal TAP tag fused 018 ORF + downstream flanking region was then amplified by PCR using primers that added a 20-bp overhang sequence complementary to the upstream flanking PCR product. The two PCR products were then joined by overlapping PCR and the product was ligated into pUC13-Ecogpt-EGFP using *Pst*I and *Bam*HI cloning sites.

To construct vΔ018, CV-1 cells at 70% confluency were infected with VACV (strain WR) at 0.05 pfu/cell and after 90 min, the inoculum was removed and cells were transfected with 7.5 μg of pUC13-Ecogpt-EGFP-Δ018 using Transit-LT1 (Mirus Bio). Two days p.i., cells were harvested by scrapping cells into the culture medium. Cells were then collected by centrifugation (500 RCF) and resuspended in 0.5 mL of infection medium (DMEM supplemented with 2% (v/v) FBS and PS (50 μg/mL). Samples were freeze-thawed three times and sonicated to lyse cells, release progeny virus and disperse particulate material. Virus dilutions were used to inoculate BS-C-1 cells that had been preincubated in infection medium, supplemented with mycophenolic acid (25 μg/mL; MPA, Sigma-Alrich), xanthine (250 μg/mL; X, Sigma-Alrich) and hypoxanthine (15 ug/mL; HX, Sigma-Alrich) for 24 h. After 90 min, the inoculum was removed and replaced with a MEM, 1% (w/v) low gelling temperature agarose (Sigma Alrich), supplemented with MPA, HX, and X. After three days, EGFP-expressing plaques were picked, representing virus that had integrated the pUC13-Ecogpt-EGFP-Δ018, and then further plaque purified in the absence of MPA, HX and X. The genotype of these plaques was then determined by PCR using primers that flank the 018 ORF (Table S3) and VACVs containing the desired mutation (vΔ018) or wild type genotype were isolated. vTAP-018 was produced using the same strategy as described above, except that vΔ018 was used as the parental VACV into which the TAP-018 ORF was inserted at its natural locus. Stocks of VACVs were grown in RK13 cells and titrated by plaque assay on BS-C-1 cells.

Purification of VACVs by sedimentation through sucrose

VACVs were semi-purified by two rounds of ultracentrifugation through a sucrose cushion as described (Joklik, 1962) and stocks were resuspended in 1 mM Tris-HCl pH 9.0 for cell culture work or in Hank's balanced salt solution (HBSS) + 0.1% (w/v) BSA for *in vivo* work. Virus titres were determined by plaque assay.

Viral DNA for vΔ018 and wild-type sibling virus v018 was isolated from semi-purified virus stocks by phenol:chloroform extraction. Whole genome sequencing of viruses was performed by MircobesNG and virus sequences were aligned to each other and VACV strain WR reference genome (VACV-WR, GenBank: AY243312.1).

VACV infection for cell culture

Virus inoculums were prepared in DMEM supplemented with 2% (v/v) FBS (infection medium) and virus adsorption was performed at 4 °C for 1 h with gentle agitation every 10 mins. At time 0 h, the virus inoculum was removed and replaced with infection medium, and infection was continued at 37 °C.

Virus growth and spread assays

For virus growth curves, confluent BS-C-1 cells were infected at 5 pfu/cell. At 1, 8 and 24 h p.i., extracellular and cell-associated virus were harvested by collecting either the supernatant or cell monolayers, respectively. Supernatants were cleared by centrifugation (21,000 RCF) to remove detached cells and debris. Cell monolayers were scrapped into new medium and subjected to three cycles of freeze-thawing followed by sonication to release intracellular virus. For virus yields in A549 cells, confluent cells were infected at 5 pfu/cell. At 24 h p.i., cells were scrapped into their culture medium and subjected to three cycles of freeze-thawing followed by sonication. Viral titres were determined by plaque assay on BS-C-1 cells.

Virus spread was determined by analysis of plaque size growth. Confluent BS-C-1, RK13 and A549 cells in 6-well plates were infected with 30 pfu/per well. At 1 h p.i., the medium was replaced with MEM supplemented with 2 mM L-glutamine, 2% (v/v) FBS and 1.5% (w/v) carboxymethylcellulose (Sigma-Aldrich). At 72 h p.i., the semi-solid overlay was removed and monolayers were stained with crystal violet (Sigma-Aldrich).

Interferon stimulations

All stimulation with IFNα (Sigma-Aldrich) and IFNγ (PreproTech) were performed using 1000 units (U)/mL or 25 ng/mL (final concentration), respectively.

Immunoblotting

For immunoblotting analysis, cells were washed in chilled PBS and harvested on ice by scrapping into lysis buffer (50 mM Tris pH 8.0, 150 mM NaCl and 1% (v/v) NP-40, supplemented with protease (cOmplete Mini, Roche) and phosphatase inhibitors (PhosSTOP, Roche). Cell lysates were incubated with rotation at 4 °C for 15 min before being cleared by centrifugation (21,000 RCF) and protein concentrations were determined using BCA Protein Assay (Pierce). Lysates were mixed with 4X SDS-gel loading buffer and incubated at 100 °C for 5 min to denature protein. Samples were centrifuged (17,000 RCF) before loading onto either SDS-polyacrylamide gels or NuPAGE (4 to 12%, 1 mm, Bis-Tris gels (Thermo Fisher Scientific) along with protein ladder (Abcam) and separated by electrophoresis. Protein gels were incubated in transfers buffer (25 mM Tris, 250 mM glycine, 20% (v/v) methanol) with agitation for 15 min. Proteins were transferred onto a nitrocellulose transfer membrane (0.2 μM pore size, GE Healthcare) using a semi-dry transfer system (Trans-tubro blot, BioRad). Nitrocellulose membranes were allowed to dry for 30 min and then blocked with 5% (w/v) BSA (Sigma), in TBS containing 0.1% (v/v) Tween-20 for 1 h at room temperature (RT). Primary antibodies (see Key Resources Table) were diluted in blocking buffer and incubated with membranes overnight at 4 °C. Membranes were probed with fluorophore-conjugated secondary antibodies (LI-COR Biosciences) diluted in 5% (w/v) non-fat milk in PBS containing 0.1% (v/v) Tween-20 and incubated at RT for 45 min. Membranes were imaged using the Odyssey CLx imagining system (LI-COR Biosciences). Protein band intensities were quantified using Image Studio software (LI-COR Biosciences).

Reporter gene assays

HeLa cells (for GAS-Luc reporter) or HEK 293T cells (for ISRE-Luc and IFNβ-Luc) were co-transfected with 75 ng of firefly luciferase reporter (GAS-Luc, ISRE-Luc or IFNβ-Luc), 10 ng of *TK-Renilla* plasmid and the desired expression plasmid using Trans-LT1 (Mirus Bio). In cases where different doses of the expression plasmids were used, the lower doses were topped up by addition of EV plasmid so that equal amounts of DNA were transfected per well. Twenty-four h post transfection, cells were either non-stimulated, or stimulated with IFNα (1000 U/mL, Sigma-Aldrich), IFNγ (25 ng/mL, PreproTech) or SeV (a gift from Steve Goodbourn) for 6, 8 or 24 h, respectively. Following stimulation, cells were lysed in passive lysis buffer (Promega) and firefly and *Renilla* luciferase luminescence were measured using a FLUOstar luminometer (BMG). Firefly values were normalised to *Renilla* luciferase readings and fold inductions were calculated for each sample relative to their own non-stimulated values. Results are presented as individual data point without P values. Relative protein expression levels were determined by immunoblotting.

Immunofluorescence

For VACV infection, A549 cells were seeded onto sterile glass coverslips and 24 h later were serum-starved for 16 h prior to infection at 10 pfu/cell. At 2 h p.i., cells were washed once in medium before being stimulated with IFNγ (25 ng/mL, PreproTech) for 30 min.

CellPress
OPEN ACCESS

For transfection, HeLa cells were seeded onto sterile glass coverslips and 24 h later cells were transfected with 0.8 μg of expression plasmids using TransIT LT1 (Mirus Bio). Five h post transfection, the medium was replaced with serum-free medium to serum-starve cells for 16 h. Cells were then either non-stimulated, stimulated with IFNα (1000 U/mL, Sigma-Aldrich) or stimulated with IFNγ (25 ng/mL, Prepotech) for 1 h.

Following stimulation, cells were fixed in 8% (v/v) paraformaldehyde (PFA, Electron Microscopy Sciences) in 250 mM HEPES pH 7.5 for 5 min on ice followed by 25 min at RT. After fixation, cells were incubated for 5 min with 50 mM ammonium chloride in PBS to quench free aldehydes. Cells were permeabilised by incubating with ice-cold, 100% methanol at -20 °C for 10 min. Cells were blocked in IF buffer (10% (v/v) FBS in PBS) for 30 min before staining with primary antibodies (see Key Resources Table) for 1 h. After washing, coverslips were then stained with secondary antibodies (AlexPhore) diluted in IF buffer, supplemented with 5% (v/v) serum from primary antibody source animal for each secondary antibody for 30 min. Coverslips were then mounted using Mowiol 4-88 containing 4',6-diamidino-2-phenylindole (DAPI) on to microscope slides (Menzel-Gläser). Slides were visualised with a LSM 780 inverted confocal microscope (Zesis) and images were processed using ImageJ (Schindelin et al., 2012).

RT-qPCR

A549 cells were infected at 10 pfu/cell. At 2 h p.i. cells were washed once in medium before being stimulated with IFNγ (25 ng/mL, PreproTech) for 1 h. Following stimulation, RNA was harvested using Monarch Total RNA Miniprep Kit (NEB) according to the manufacturer's instructions including an optional on-column genomic DNA digestion step. RT-qPCR was performed using Luna Universal One-Step RT-qPCR Kit (NEB). Oligonucleotide primers (Table S2) targeting HRPT and IRF1 were designed using PrimerQuest Tool (IDT). RT-qPCRs were carried out using a real-time PCR system (Thermo Fisher Scientific) and fold-inductions of ISG levels were calculated using $2^{-\Delta\Delta Ct}$ taking mock non-stimulated readings as the basal level sample and HRPT as the control housekeeping gene.

Pulldowns

For infection, BS-C-1 cells and MEFs were infected at 10 pfu/cell with either vTAP-018 or vTAP-N1, whereas A549 cells were infected at 5 pfu/cell with either vTAP-018 or vFLAG-A36. For transfection, 2fTGH, U3A, U6A and HEK 293T cells were transfected using either TranIT LT1 (Mirus Bio) for 2fTGH, U6A and U3A cells or polyethylenimine (PEI, 2 μl of 1 mg/mL stock per μg of DNA, Polysciences) for HEK 293T cells. Prior to transfection, medium was replaced with DMEM supplemented with 2% (v/v) FBS. At 12 h p.i. or 18 h after transfection, cells were lysed in Tris-based IP buffer (50 mM Tris pH 7.4, 150 mM NaCl, 0.5% (v/v) NP-40) supplemented with protease (cOmplete Mini, Roche) and phosphatase inhibitors (PhosSTOP, Roche). Cell lysates were incubated with rotation at 4 °C for 15 min before being cleared by centrifugation (21,000 RCF). A fraction of cleared lysate was taken for input samples and the remaining lysate was incubated with 30 μl of one of the following affinity resins that had been washed and equilibrated in IP buffer: (i) Strep-Tactin XP super flow (IBA) for pull-down of TAP-tagged proteins via Strep-tag II epitope; (ii) anti-FLAG M2 affinity gel (Sigma-Aldrich) for immunoprecipitation of either FLAG or TAP-tagged protein via the FLAG epitope. Samples were incubated with affinity resins at 4 °C with rotation for 1 h 30 min. Samples were washed three times in IP buffer and proteins were eluted from beads by addition of 2X SDS-gel loading buffer. Subsequently, samples were analysed by either Nu-PAGE (Thermo Fisher Scientific) or SDS-PAGE followed by immunoblotting.

For pulldowns using proteins produced from a cell-free transcription and translation system, the TnT Sp6 High-Yield wheat germ protein expression system (Promega) was utilised according to the manufacturer's instructions.

In vivo experiments

Female BALB/c mice 6-10 weeks old were anesthetized and infected intranasally (i.n.) with 10^3 pfu for measurement of weight change and pulmonary virus titres, or 10^5 pfu for analysis of anti-viral genes by RT-qPCR. A final inoculation volume of 20 μl (10 μl per nostril) was used with VACVs diluted in HBSS + 0.1% (w/v) BSA to achieve the required dose. The actual dose administered was confirmed by plaque assay of the diluted virus inoculum.

For weight change experiments, mice were weighed daily. For virus titration experiments, lungs were collected at 3, 7 and 9 days p.i. and single-cell suspensions were prepared by chopping with scissors followed by collagenase I digestion (Worthington Biochemicals) for 60 min at 37 °C. Cells were disrupted by vigorous pipetting and suspensions were freeze-thawed three times to release virus and infectious virus titres were determined by plaque assay on TK^-143B cells. For RT-qPCR analysis, the upper lobes of lungs were removed and immediately placed in buffer RLT (Qiagen). Lungs were homogenized and RNA was isolated using Lysing Matrix S (1/8") metal beads (MPBio) and a FastPrep®-24 Instrument (MPBio). RNA was then purified using a Qiagen RNeasy Mini Kit (Qiagen). An on-column DNAse (Sigma-Aldrich) digestion was performed prior to RNA elution. cDNA was synthesised using the RT^2 First Strand Kit (Qiagen) with ~ 1.2 mg of RNA/sample. cDNA was then loaded onto an Antiviral Response qPCR array (Qiagen) or onto a separate plate for the analysis of IRF1 and IFNγ for which individual RT^2 qPCR primer assays (Qiagen) were obtained. RT-qPCRs were carried out using RT^2 SYBR Green ROX qPCR mastermix (Qiagen) and a real-time PCR system (Thermo Fisher Scientific) and fold-changes of genes were calculated by comparing Ct values of individual vΔ018-infected mice (n=4) to the Ct averages of v018-infected mice (n=3) using the $2^{-\Delta\Delta Ct}$ method. Fold changes of genes were normalised against 5 standard housekeeping genes included on the Antiviral Response qPCR array (Qiagen) or against 3 standard housekeeping genes (Actb, B2M and GAPDH, Qiagen) for analysis of IRF1 and IFNγ (Qiagen). Data analysis and significances were performed using the manufacturer's software (GeneGlobe Data Analysis Centre, Qiagen).

Protein expression and purification

The purity of protein preparations was analysed by SDS-PAGE and subsequent Coomassie blue staining (Figure S7).

Full-length STAT1 and STAT1$^{132\text{-}684,\Delta183\text{-}190,H182A,E393A,E394A}$ expression plasmids were transformed into *E. coli* T7 Express cells (NEB) and plated overnight on LB agar supplemented with 100 μg/mL of ampicillin. The next day colonies of transformed cells were collected and used to inoculate 1 L of terrific broth (TB) medium supplemented with 100 μg/mL of ampicillin and were grown at 37 °C in 2 L flasks until OD_{600} of 0.8-1.2. Cultures were cooled to 18 °C and incubated overnight with 0.4 mM IPTG to induce protein expression. Cells were collected by centrifugation and resuspended in lysis buffer (25 mL of 50 mM Tris-HCl, pH 8.0, 300 mM NaCl, 20 mM imidazole, 1 mM AEBSF, 1 mM TCEP) and lysed by sonication. Cell lysates were centrifugated at 40,000 RCF for 30 min and the cleared supernatant was loaded on a 3 mL Ni-NTA agarose resin (Cube Biotech) or on a 5mL HisTrap HP column (Cytiva). The column matrix was washed with 10 column volumes (CV) of wash buffer (50 mM Tris-HCl, pH 8.0, 300 mM NaCl, 20 mM imidazole, 1 mM TCEP). Proteins were eluted with 50 mM Tris-HCl, pH 8.0, 300 mM NaCl, 200 mM imidazole, 1 mM TCEP into 2 mL fractions. Fractions containing the proteins of interest were pooled. STAT1$^{132\text{-}684,\ \Delta183\text{-}190,\ H182A,\ E393A,\ E394A}$ fractions were incubated with 100 μL of 2 mg/mL TEV protease (prepared in-house) overnight at 4 °C to remove the N-terminal His_6 affinity tag. STAT1 proteins were then diluted ten-fold in heparin buffer A (20 mM Tris-HCl, pH 8.0, 1 mM EDTA) and loaded on a 5 mL HiTrap Heparin HP column (Cytiva) equilibrated with the same buffer. The column matrix was washed with 10 CV of heparin buffer A, followed by elution with a 0-100% linear gradient of heparin buffer B (20 mM Tris-HCl pH 8.0, 1 mM EDTA, 1 M NaCl). STAT1 and STAT1(core)$^{\Delta183\text{-}190,\ EE}$ eluted at approximately 20% heparin buffer B. Fractions containing the protein of interest were supplemented with TCEP (1 mM final) and concentrated on a centrifugal filter (molecular weight cut off (MWCO) 30,000 Da, Amicon) to 2 mL, after which the proteins were loaded on a Superdex 200 16/600 GL (Cytiva) size-exclusion chromatography (SEC) column equilibrated with 20 mM Tris-HCl pH 8.0, 300 mM NaCl, 1 mM EDTA. SEC fractions corresponding to the later-eluting major peak were pooled and supplemented with TCEP (1 mM final), concentrated to ~0.5 mM on a centrifugal filter (MWCO 30,000 Da, Amicon) and flash-frozen in liquid nitrogen.

GB1-018 and GB1-NiV-V fusions were expressed from pPEPT plasmids (TP, unpublished, Figure S8) that were transformed into *E. coli* T7 Express cells (NEB) and plated overnight on LB agar supplemented with 100 μg/mL of ampicillin. The next day transformed cells were collected and used to inoculate 1 L TB medium supplemented with 100 μg/mL of ampicillin and were grown at 37 °C in 2 L flasks until OD_{600} of 0.8-1.2. Protein expression was induced with 0.4 mM IPTG for 3 h at 37 °C. Following bacterial expression, a nickel affinity purification step was performed as described for STAT1 proteins. Fractions containing protein of interest were concentrated on a centrifugal filter (Amicon, MWCO 3,000 Da) to 2 mL, after which the proteins were loaded on a Superdex 75 or Superdex 200 16/600 GL (Cytiva) SEC columns equilibrated with 20 mM Tris-HCl pH 8.0, 300 mM NaCl, 1 mM EDTA. SEC fractions corresponding to GB1 fusions were pooled, concentrated on a centrifugal filter (MWCO 3,000 Da, Amicon) and flash-frozen in liquid nitrogen. For the purification of NiV-V proteins, buffers were supplemented with TCEP (1 mM final) to maintain cysteines in a reduced state.

Isothermal titration calorimetry (ITC)

All proteins were buffer-exchanged into ITC buffer (50 mM Tris-HCl pH 8.0, 150 mM NaCl, 1 mM EDTA, 0.1% (v/v) Tween-20) using a NAP-5 size-exclusion column (Cytiva) and concentrations were determined by UV/Vis spectrophotometry and adjusted as needed. For measurements with synthetic peptides, peptides were dissolved from lyophilised powder in MilliQ water and then concentrations were measured by UV-Vis and were adjusted to 10x the final value. Thereafter, the peptides were diluted ten-fold in ITC buffer. ITC measurements were performed on a Microcal ITC200 instrument (GE Healthcare) with 18 x 2 μL injections, 160 s interval and 5 μCal s^{-1} reference power. Baseline correction was performed using injection heats from protein-into-buffer runs. Integration of thermogram peaks and fitting of data was done using the Malvern ITC package in Origin 7.0 (Originlab). Isotherm fitting was performed using a one site model. Initial low volume injection is excluded from all analysis. All reaction conditions and fitted parameters are shown in Table S5.

Fluorescence polarisation (FP) anisotropy measurements

All proteins were buffer-exchanged into FP buffer (50 mM Tris pH 8.0, 300 mM NaCl, 1mM EDTA, 0.1% (v/v) Tween-20, 1 mM TCEP) using a NAP-5 size-exclusion column (Cytiva) and concentrations were determined by UV/Vis spectrophotometry and adjusted as needed, after which, BSA was added to 0.1% (w/v). Fluorescein-conjugated pIFNGR1 12-mer peptide probe (Fluor-pIFNGR1) was first dissolved in DMSO to 10 mM and then diluted in FP buffer plus 0.1% (w/v) BSA to the required concentration. Reactions (40 μL) were set up in a 384-well non-transparent microplate (Corning, #3542). Competition reactions were performed with 10 nM Fluor-pIFNGR1 and fixed STAT1 concentration of 1.5 μM and 2-fold serial dilutions of 018 or NiV-V GB1 fusions. Each dilution was measured in triplicate. Graphs show means ± SD (n=3) per dilution.

Measurements were performed on a Pherastar FS plate reader (BMG) using a FP 485/520/520 optical module. Reactions containing only 10 nM Fluor-pIFNGR1 were prepared as reference standards and were used to calibrate gain and focal height. Dose-response curves were fitted in Prism 9.0.0 (GraphPad) using a four-parameter logistic regression.

Peptides for ITC and FP

A 5-mer sequence (pYDKPH) of the pIFNGR1 is responsible for the vast majority of the receptor STAT1 SH2 domain interaction. For the FP assay we utilised a 12-mer peptide (5Flu-GTSFGpYDKPHVLV-NH2, PeptideSynthetics, UK) where TSFGpYDKPHVLV corresponds to 12 aa from pIFNGR1 and 5Flu-G represents an N-terminal 5-carboxyfluorescein and a spacer glycine. For ITC measurement we utilised the 5-mer peptide (Ac-pYDKPH-NH2, Genosphere Biotechnologies) due to greater solubility compared

CellPress
OPEN ACCESS

to the 12-mer peptide. Peptides were prepared using Fmoc-based solid-phase synthesis and purity was >95% as determined by HPLC.

SEC-MALS

SEC-MALS was performed using a Superdex 200 Increase 10/300 column (Cytiva) equilibrated with 50 mM Tris pH 8.0, 300 mM NaCl, 1 mM EDTA, 1 mM TCEP. The column was connected to a DAWN HELEOS II light scattering detector (Wyatt Technology) and the Optilab T-rEX refractive index detector (Wyatt Technology). Scattering was detected at 664 nm wavelength at RT. One hundred μL of sample was applied at a concentration of 20 μM for STAT1 and 100 μM of GB1-018. The experimental data were recorded and processed using the ASTRA software (Wyatt Technology).

X-ray crystallography

A STAT1 core fragment crystallography construct (STAT1$^{132\text{-}684,\Delta183\text{-}190,H182A,E393A,E394A}$) was prepared harbouring a loop deletion at the apex of the coiled coil domain (Δ183-190,H182A) and surface entropy-reducing mutations (E393A,E394A). The STAT1-018 complex was co-crystallised using sitting-drop vapour diffusion in a 96-well MRC plate format. The complex was prepared by mixing STAT1$^{132\text{-}684,\Delta183\text{-}190,H182A,E393A,E394A}$ in SEC buffer (20 mM Tris-HCl pH 8.0, 300 mM NaCl, 1 mM EDTA) and 018 21-mer peptide (Ac-MWSVFIHGHDGSNKGSKTYTS-NH2, Genosphere Biotechnologies) in 20 mM Tris-HCl pH 8.0, to a final concentration of 5 mg/mL protein and 2 mg/mL peptide. Three hundred nL of the complex was mixed with 300 nL of the crystallisation condition using a Mosquito liquid handling robot (TTP Labtech). Crystals were obtained using the following condition: 16% (v/v) PEG 3350, 175 mM KCl, 125 mM $(NH_4)_2SO_4$. Cryoprotectant solution containing the crystallisation condition and 30% (v/v) ethylene glycol was added to the drop and crystals were incubated for 1 min. A crystal was then harvested and cryo-cooled in liquid nitrogen. Diffraction data were collected at Diamond Light Source (Harwell, UK) synchrotron radiation source, beamline i04. Diffraction images were processed with autoPROC (Vonrhein et al., 2011). Molecular replacement phasing was used with STAT1 core residues 133-683 (PDB ID: 1YVL) as a search model. The structure was refined without peptide first and the peptide was built into the clearly visible electron density manually (Figure S6A). Manual real-space refinement was done in Coot (Emsley et al., 2010) and automated refinement with phenix.refine (Liebschner et al., 2019) and autoBUSTER (Smart et al., 2012). Crystallographic data and refinement statistics are shown in Table S4. The coordinates and corresponding structure factors have been deposited to the PDB under accession number PDB: 7nuf.

QUANTIFICATION AND STATISTICAL ANALYSIS

Significances were calculated in Prism (GraphPad) by either Dunnett's T3 multiple comparisons test or Unpaired t-test with Welch's correction as indicated. For anti-viral array data (Figure 4G), analysis and Unpaired t-tests were performed using GeneGlobe Data Analysis Centre (Qiagen). All significances are indicated with P values.

Cell Host & Microbe, Volume *30*

Supplemental information

Poxviruses and paramyxoviruses use a conserved mechanism of STAT1 antagonism to inhibit interferon signaling

Callum Talbot-Cooper, Teodors Pantelejevs, John P. Shannon, Christian R. Cherry, Marcus T. Au, Marko Hyvönen, Heather D. Hickman, and Geoffrey L. Smith

Table S1. Oligonucleotide primers for construction of recombinant DNA. Related to STAR methods. Cloning sites are highlighted in bold (for restriction digest cloning) or underlined (for ligation-independent cloning). Complementary sequences for overlapping PCR are italicised. Site-directed mutagenesis primers are indicated with an asterisk (*). All V5 and TAP-tagged proteins are tagged at the N terminus.

Plasmid	Primers 5'-3'
pcDNA4/TO TAP-018	AAA**GCGGCCGC**GATGAGTTCTAAAGGCGGATCTTC
	AAA**TCTAGA**TTAGATTTTGCCGGTGCCGC
pcDNA/TO TAP-NiV-V	AAAA**GCGGCCG**CGGATAAATTGGAACTAGTCAA
	AAAA**TCTAGA**TTAACCGCAGTGGAAGCATTCAG
pcDNA4/TO TAP-018 (1-54)	AAA**GCGGCCGC**GATGAGTTCTA
	AAA**TCTAGA**TTATTTCACGCCGCCGTTCACGC
pcDNA4/TO TAP-018 (1-48)	AAA**GCGGCCGC**GATGAGTTCTA
	AAA**TCTAGA**TTAGCCAGACTTCACTCCGCTA
pcDNA4/TO TAP-018 (1-43)	AAA**GCGGCCGC**GATGAGTTCTA
	AAA**TCTAGA**TTAGCTAGATGATCCTCCGCCCCA
pcDNA4/TO TAP-018 (1-35)	AAA**GCGGCCGC**GATGAGTTCTA
	AAA**TCTAGA**TTATCCTCCGCCGCCAGATGT
pcDNA4/TO TAP-018 (1-30)	AAA**GCGGCCGC**GATGAGTTCTA
	AAA**TCTAGA**TTATGTGTATGTCTTGCTGCCCT
pcDNA4/TO TAP-018 (1-27)	AAA**GCGGCCGC**GATGAGTTCTA
	AAA**TCTAGA**TTACTTGCTGCCCTTGTTGCTG
pcDNA4/TO TAP-018 (1-24)	AAA**GCGGCCGC**GATGAGTTCTA
	AAA**TCTAGA**TTACTTGTTGCTGCCATCGTGG
pcDNA4/TO TAP-018 (1-21)	AAA**GCGGCCGC**GATGAGTTCTA
	AAA**TCTAGA**TTAGCCATCGTGGCCGTGGAT
pcDNA4/TO TAP-018 (8-60)	AAA**GCGGCCGC**GTCTGGCGGCATGTGGAGC
	AAA**TCTAGA**TTAGATTTTGCCGGTGCCGCTT
pcDNA4/TO TAP-018 (11-60)	AAA**GCGGCCGC**GATGTGGAGCGTGTTCATCCA
	AAA**TCTAGA**TTAGATTTTGCCGGTGCCGCTT
pcDNA4/TO TAP-018 (14-60)	AAA**GCGGCCGC**GGTGTTCATCCACGGCCACG
	AAA**TCTAGA**TTAGATTTTGCCGGTGCCGCTT
pcDNA4/TO TAP-018 (17-60)	AAA**GCGGCCGC**GCACGGCCACGATGGCAG
	AAA**TCTAGA**TTAGATTTTGCCGGTGCCGCTT
pcDNA4/TO TAP-018 (22-60)	AAA**GCGGCCGC**GAGCAACAAGGGCAGCAAGAC
	AAA**TCTAGA**TTAGATTTTGCCGGTGCCGCTT
pcDNA4/TO TAP-018 (31-60)	AAA**GCGGCCGC**GTCTGGCGGCGGAGGAATGT
	AAA**TCTAGA**TTAGATTTTGCCGGTGCCGCTT
pcDNA4/TO TAP-018^{AGA}	CGCCGATGGCAGCAACAAGGG*
	CCGGCGATGAACACGCTCCACATG*
pcDNA3 HA-018	SUBCLONED FROM PCDNA4/TO TAP-018
pcDNA3 HA-018^{AGA}	CGCCGATGGCAGCAACAAGGG*
	CCGGCGATGAACACGCTCCACATG*
pcDNA3 V5-STAT1	AAAA**GCGGCCGC**GTCTCAGTGGTACGAACTTCA
	AAAA**GGGCCC**TTATACTGTGTTCATCATAC
pcDNA3 V5-STAT2	AAA**GCGGCCGC**GATGGCGCAGTGGGAAATGCT
	AAA**CTCGAG**TTAGAAGTCAGAAGGCATCAAG
pcDNA3 V5-STAT3 (human)	AAA**GCGGCCGC**GATGGCCCAATGGAATCAGCT
	AAA**CTCGAG**TTACATGGGGGAGGTAGCGC
pcDNA3 V5-STAT3 (mouse)	AAA**GCGGCCGC**GGCTCAGTGGAACCAGCTGCA
	AAA**GGGCCC**TCACATGGGGGAGGTAGCAC
pcDNA3 V5-STAT4	AAA**GCGGCCGC**GATGTCTCAGTGGAATCAAGTCC
	AAA**CTCGAG**TTATTCAGCAGAATAAGGAGACTTC
pcDNA3 V5-STAT5A	AAA**GCGGCCGC**GATGGCGGGCTGGATCCA
	AAA**CTCGAG**TTATGAGAGGGAGCCTCTGGC
pcDNA3 V5-STAT5B	AAA**GCGGCCGC**GATGGCTGTGTGGATACAAGCTCA
	AAA**CTCGAG**TTACGATTGTGCGTGCGGGA
pcDNA3 V5-STAT6	AAA**GCGGCCGC**GATGTCTCTGTGGGGTCTGGTC
	AAA**CTCGAG**TTACCAACTGGGGTTGGCCCT
pcDNA3 V5-STAT1 (1-712)	AAAA**GCGGCCGC**GTCTCAGTGGTACGAACTTCA
	AAAA**GGGCCC**TTAAACTTCAGACACAGAAATCAA
pcDNA3 V5-STAT1 (1-684)	AAAA**GCGGCCGC**GTCTCAGTGGTACGAACTTCA
	AAAA**GGGCCC**TTATGGCCTGGAGTAATACTTTC
pcDNA3 V5-Fus1	AAAA**GCGGCCGC**GGCTCAGTGGAACCAGCTGCA
	*CCCTTCATTCCAAAG*GGCCAAG
	*CTTTGGAATGAAGGG*TGCATCATGGGCTTCAT
	AAAA**GGGCCC**TTATACTGTGTTCATCATAC
pcDNA3 V5-Fus2	AAAA**GCGGCCGC**GTCTCAGTGGTACGAAC
	CCCATCATTCCAGAGAGGGA
	*TCCCTCTCTGGAATGATGGG*TACATCATGGGTTTCATCAG
	AAAA**GGGCCC**TTACATGGGGGAGGTAGCACAC
pcDNA3 V5-STAT3^{Q635H} (Human)	GACCCAGATCCACTCCGTGGAAC*
	TTACCGCTGATGTCCTTC*
pF3A TAP-018	AAA**CGATCG**ATGTGGTCTCATCCTCAGTT
	AAA**GTTTAAAC**TTAGATTTTGCCGGTGCCGC
pF3A STAT1	AAA**CGATCG**ATGTCTCAGTGGTACGAACTTCA
	AAA**GTTTAAAC**TTATACTGTGTTCATCATAC
pUC13-Ecogpt-EGFP Δ018	AAA**CTGCAG**TGCCACTGTCTAGTAGTAGT
	AAA*GCGGCCGCATACTCGAG*ACACTAAATTATTTTTATTA
	AAA*CTCGAGTATGCGGCCGC*ATTAATAAGACTTAAGGACA
	AAA**GGATCC**CTAATAAGTTAATAGAACAA
pUC13-Ecogpt-EGFP TAP-018	AAA**TCTAGA**TGCCACTGTCTAGTAGTAGT.
	AAA**GCGGCCGC**GATGTCTTCTAAAGGGGGTA
	ATTAATAAGACTTAAGGACA.
	AAA**GGATCC**GCTAATAAGTTAATAGAACAA.
	AAA**CTGCAG**TGCCACTGTCTAGTAGTAGT.
	*TGTCCTTAAGTCTTATTAAT*ATGTGGTCTCATCCTCAGTT
pOPTH_TEV	CCGGAGAGCTCCAATTGGA.
	AAA**GGATCC**CTGGAAGTACAGGTTTTCGGTACCGCTATGGTGATGGTGATGATGAGCCAT
pOPTH_TEV STAT1$^{132-684,\Delta183-190,H182A,E393A,E394A}$	AAA**GGATCC**ACAGTGATGTTAGACAAACAG
	AAA**ACGCGT**TTATGGCCTGGAGTAATACTTT
	*TTGCAGAACAGAGAA*GCGGATCAGAAACAAGAACAGCTGTTACTC
	*TTCTCTGTTCTGCAA*GGTTTTG
	GATGAACATGGCGGCGTCCACCAATG*
	ACTTTTGTGTGCGTGCCC*
pEXP-nHis-STAT1-cStrep	GAAAACCTGTACTTCCAGGGTTCTCAGTGGTACGAACTTCAGCAG
	GCTCCATGCGCTCGATACTGTGTTCATCATACTGTCGAATTC
pPEPT 018	GGAA**GGATCC**GGCGGCATGAGTTCTAAAGGCGGATC
	AAA**CTCGAG**CCGATTTTGCCGGTGCCGCTTTTC
pPEPT 018^{T2}	GGAA**GGATCC**GGCGGCATGTGGAGCGT
	AAA**CTCGAG**CCAGATGTGTATGTCTTGCTGCCC
pPEPT 018^{T3}	GGAA**GGATCC**GGCGGCATGTGGAGCGT
	AAA**CTCGAG**CCCTTGCTGCCCTTGTTGCTG
pPEPT 018^{AGA}	CGCCGATGGCAGCAACAAGGG*
	CCGGCGATGAACACGCTCCACATG*
pPEPT-NiV-V (110-140)	AAA**GGATCC**GGCGGCGTGGTTACAGACGTTGTATA
	AAA**CTCGAG**CCTGTGTAATCACTCCACCCTC
pPEPT-NiV-V (110-140)^{ADA}	TGCTGGAGGAGAATGTACCGGA*
	TCAGCGTATACAACGTCTGTAACCAC*

Table S2. Oligonucleotide primers for RT-qPCR. Related to STAR methods.

Target Gene	Primers 5'-3'
HRPT	CGAGATGTGATGAAGGAGATGG
	TTGATGTAATCCAGCAGGTCAG
IRF1	CATTCACACAGGCCGATACA
	TGGTCTTTCACCTCCTCGATA

Table S3. Oligonucleotide primers for analytical PCR or sequencing. Related to STAR methods.

Target	Primers 5-3'
STAT1 Internal	ATGCTTGCTTGGATCAGC
STAT2 Internal	GTTGGAACAGCTGGAGAC
STAT3 (human) Internal	CAACTTCAGACCCGTCAAC
STAT4 Internal	GAGCTGCAAGACTGGAA
STAT5A Internal	GATGACGAGCTGATCCAGTG
STAT5B Internal	GACGAGCTGATCCAGTGG
STAT6 Internal	GCTGGATGAAGTCCTGAG
VACWR018 Upstream	GTGAACTCTATACACCCACAC
VACWR018 Downstream	ACATTTGATTTTCTCGTACGC

Table S4. X-ray crystallographic data collection and refinement statistics. Related to Figure 7 and STAR methods.

PDB ID: 7nuf

**Data collection and processing**	
Wavelength (Å)	0.9795
Space group	C 1 2 1
Data collection temperature (K)	100
a, b, c (Å)	169.06, 37.47, 115.48
α, β, γ (°)	90.00, 116.19, 90.00
Resolution range (high resolution bin) (Å)	80.42 - 2.00 (2.00 - 2.04)
R_{meas}	0.124 (3.251)
Completeness (%)	99.7 (99.8)
Number of total / unique reflections	183215 / 44523
Redundancy	4.1 (4.3)
$\langle I/\sigma(I)\rangle$	6.6 (0.5)
$CC_{1/2}$	1.0 (0.4)
**Refinement**	
R_{cryst} / R_{free}	0.224 / 0.256
Number of reflections in test set	2136
Number of atoms	4659
Mean/Wilson B-factor (Å^2)	61 / 44.3
Ramachandran favoured/allowed/outliers (%)	98.86/ 1.14 / 0
RMSD bonds (Å)	0.009
RMSD angles (°)	1.309

Table S5. ITC experimental conditions and fitted parameters. Related to Figures 3, 5, 6, S6 and STAR methods.

Reaction	Cell	Syringe	K_d, nM	N	ΔH, cal / mol	ΔS, cal / mol / deg
018 + STAT1	10 μM STAT1	100 μM 018	291	1.02	-1.93×10^4	-34.9
018T2 + STAT1	15 μM STAT1	150 μM 018T2	235	1.01	-1.87×10^4	-32.3
018T3 + STAT1	15 μM STAT1	350 μM 018T3	$>10^4$	1.00 (fixed)	-2.01×10^4	-46.8
018AGA + STAT1	10 μM STAT1	100 μM 018AGA	No binding	n.a.	n.a.	n.a.
IFNGR1 + STAT1	10 μM STAT1	300 μM IFNGR1 5-mer	7.6×10^3	0.708	-9172	-7.33
IFNGR1 + STAT1 / 018	10 μM STAT1 + 50 μM 018	300 μM IFNGR1 5-mer	No binding	n.a.	n.a.	n.a.
IFNGR1 + STAT1 / 018 AGA	10 μM STAT1 + 50 μM 018 AGA	300 μM IFNGR1 5-mer	7.8×10^3	0.79	-8001	-3.48
IFNGR1 + STAT1 / NiV-V	10 μM STAT1 + 200 μM NiV- V	300 μM IFNGR1 5-mer	No binding	n.a.	n.a.	n.a.
IFNGR1 + STAT1 / NiV-V ADA	10 μM STAT1 + 200 μM NiV-V ADA	300 μM IFNGR1 5-mer	7.2×10^3	0.88	-8125	-3.72
018 21-mer + STAT1	10 μM STAT1	100 μM 018 21-mer	321	1.09	-1.59×10^4	-23.9
p018 21-mer + STAT1	10 μM STAT1	100 μM p018 21-mer	174	1.12	-9139	0.276

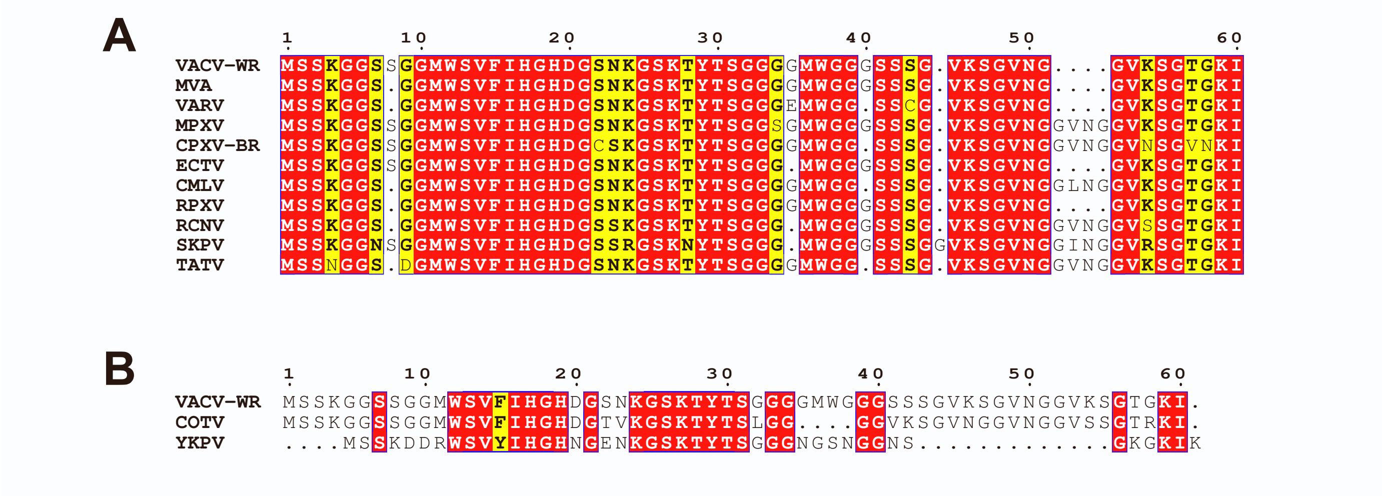

Figure S1. Sequence alignment of 018 orthologues. Related to Figure 1. (**A**) Alignment of 018 orthologues from representative orthopoxviruses: vaccinia virus strain Western Reserve (VACV-WR), modified vaccinia Ankara (MVA), variola virus (VARV), monkeypox virus (MPXV), cowpox virus strain Brighton Red (CPXV), ectromelia virus (ECTV), camelpox virus (CMPV), rabbitpox virus (RPXV), racconpox virus (RCNV), skunxpox virus (SKPV) and taterpox virus (TATV). The 018 ORF is absent from VACV strain Copenhagen. (**B**) Alignment of 018 poxvirus orthologues from Cotia virus (COTV) and yokapoxvirus (YKPV), which sit outside of the orthopoxvirus genus. Identical residues are shown in red, similar residues are shown in yellow (**A-B**).

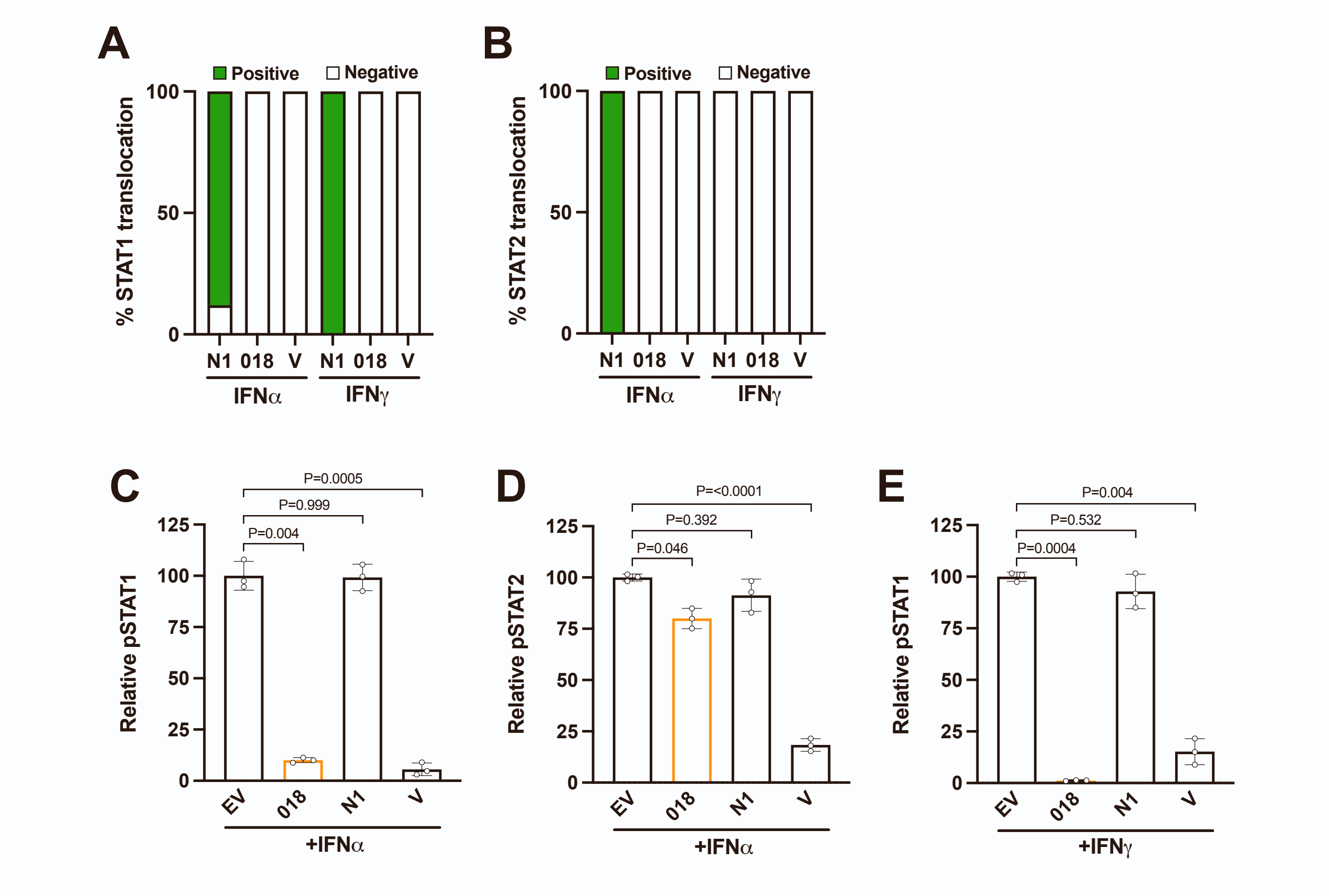

Figure S2. Quantification of STAT1 and STAT2 phosphorylation and translocation into the nucleus. Related to Figure 2. (**A-B**) Percentage of cells expressing TAP-tagged N1, 018 and NiV-V (V) that show nuclear STAT1 (**A**) and STAT2 (**B**) following stimulation with IFNα or IFNγ from (**Figure 2A, B**), (n=25 cells per condition). Cells positive for translocation are shown in green. (**C-E**) Quantification of relative band intensities for pSTAT1 (**C**) and pSTAT2 (**D**) from (**Figure 2C**) and pSTAT1 (**E**) from (**Figure 2D**). pSTAT levels were normalised against total STAT levels and made relative to EV condition. Means ± SD (n=3 per condition) are shown. Significances were calculated by Dunnett's T3 multiple comparisons test.

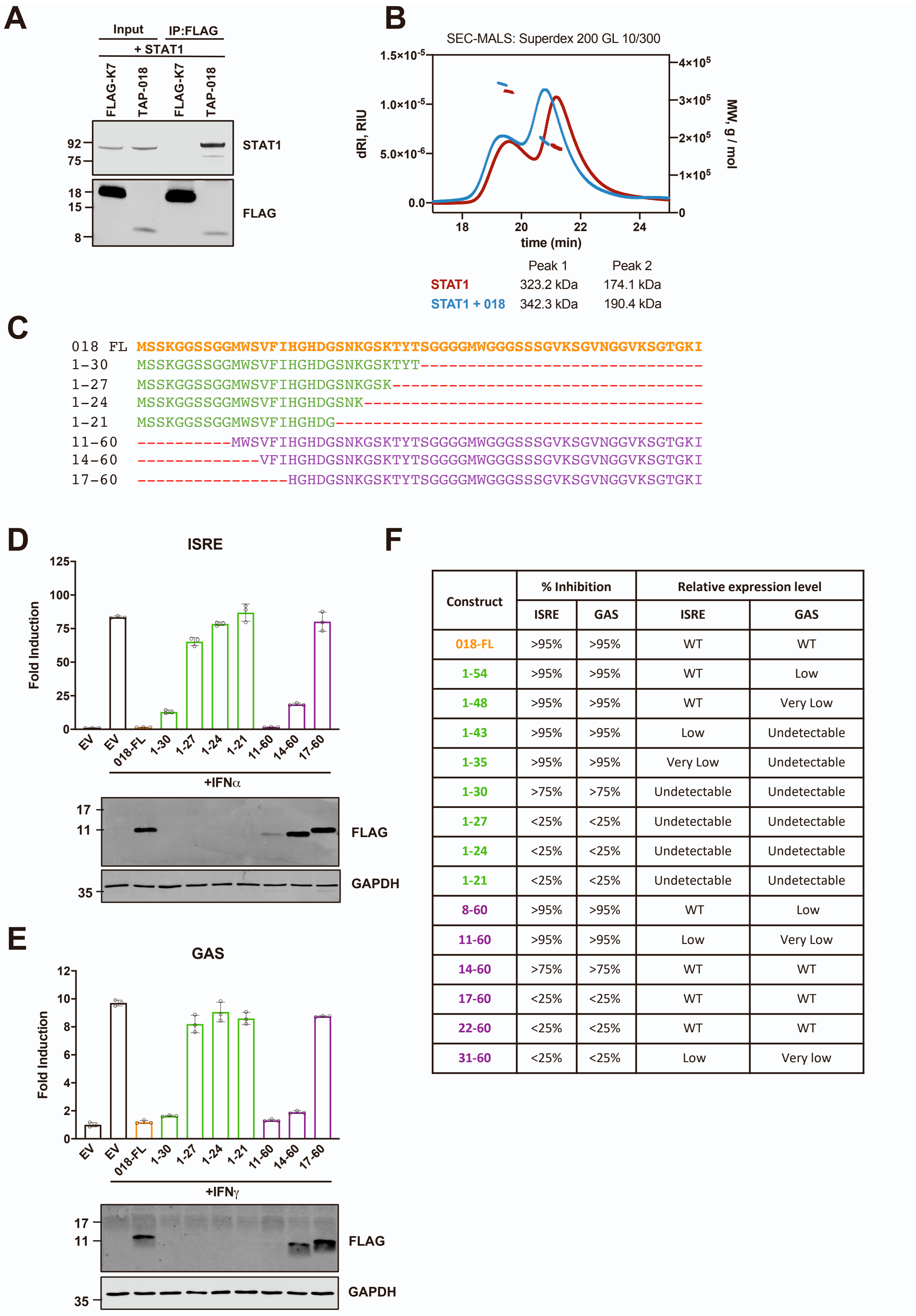

Construct	% Inhibition		Relative expression level	
	ISRE	GAS	ISRE	GAS
018-FL	>95%	>95%	WT	WT
1-54	>95%	>95%	WT	Low
1-48	>95%	>95%	WT	Very Low
1-43	>95%	>95%	Low	Undetectable
1-35	>95%	>95%	Very Low	Undetectable
1-30	>75%	>75%	Undetectable	Undetectable
1-27	<25%	<25%	Undetectable	Undetectable
1-24	<25%	<25%	Undetectable	Undetectable
1-21	<25%	<25%	Undetectable	Undetectable
8-60	>95%	>95%	WT	Low
11-60	>95%	>95%	Low	Very Low
14-60	>75%	>75%	WT	WT
17-60	<25%	<25%	WT	WT
22-60	<25%	<25%	WT	WT
31-60	<25%	<25%	Low	Very low

Figure S3. 018 binds STAT1 without affecting its oligomeric state and inhibits IFN-signalling via a 21 aa sequence. Related to Figure 3. (**A**) 018:STAT1 interaction using a cell-free transcriptional and translation system. Untagged-STAT1 was co-expressed along with either FLAG-tagged K7 or TAP-tagged 018 using a wheat germ cell free transcriptional and translation system. FLAG-K7 and TAP-018 were

precipitated using M2-FLAG affinity gel and purified proteins were analysed by immunoblotting with α-FLAG and α-STAT1 antibodies. (**B**) SEC-MALS measurements of free STAT1 (red) and STAT1:GB1-018 complex (blue). One hundred μl samples were loaded on a Superdex 200 GL 10/300 column and scattering and refractive index of the eluting peaks were measured. Concentration of 20 μM for STAT1 and 100 μM of GB1-018 were applied. (**C**) Sequences for C-terminal (green) and N-terminal (purple) 018 refined truncation mutants. (**D**) HEK 293T cells or (**E**) HeLa cells were transfected with reporter plasmids ISRE-Luc (**D**) or GAS-Luc (**E**) plus TK-*Renilla* and vectors expressing 018 truncation mutants from (**C**) fused to a TAP-tag. Cells were stimulated with IFNα (1000 U/mL) (**D**), or IFNγ (25 ng/mL) (**E**) for 6 h (**D**) or 8 h (**E**) and luciferase values were measured. Means ± SD (n=3 per condition) are shown. Lysates were prepared and analysed by immunoblotting with α-FLAG and α-GAPDH. (F) Summary of all C-terminal (green) and N-terminal (purple) 018 truncation mutants describing the percentage inhibitory activity (>95%, >75% (but less then >95%) or <25%) and relative protein expression levels (WT (wild-type)), low, very low or undetectable) for ISRE (IFNα) and GAS (IFNγ) reporters. Data from (**D-E**) are representative of 2 individual experiments.

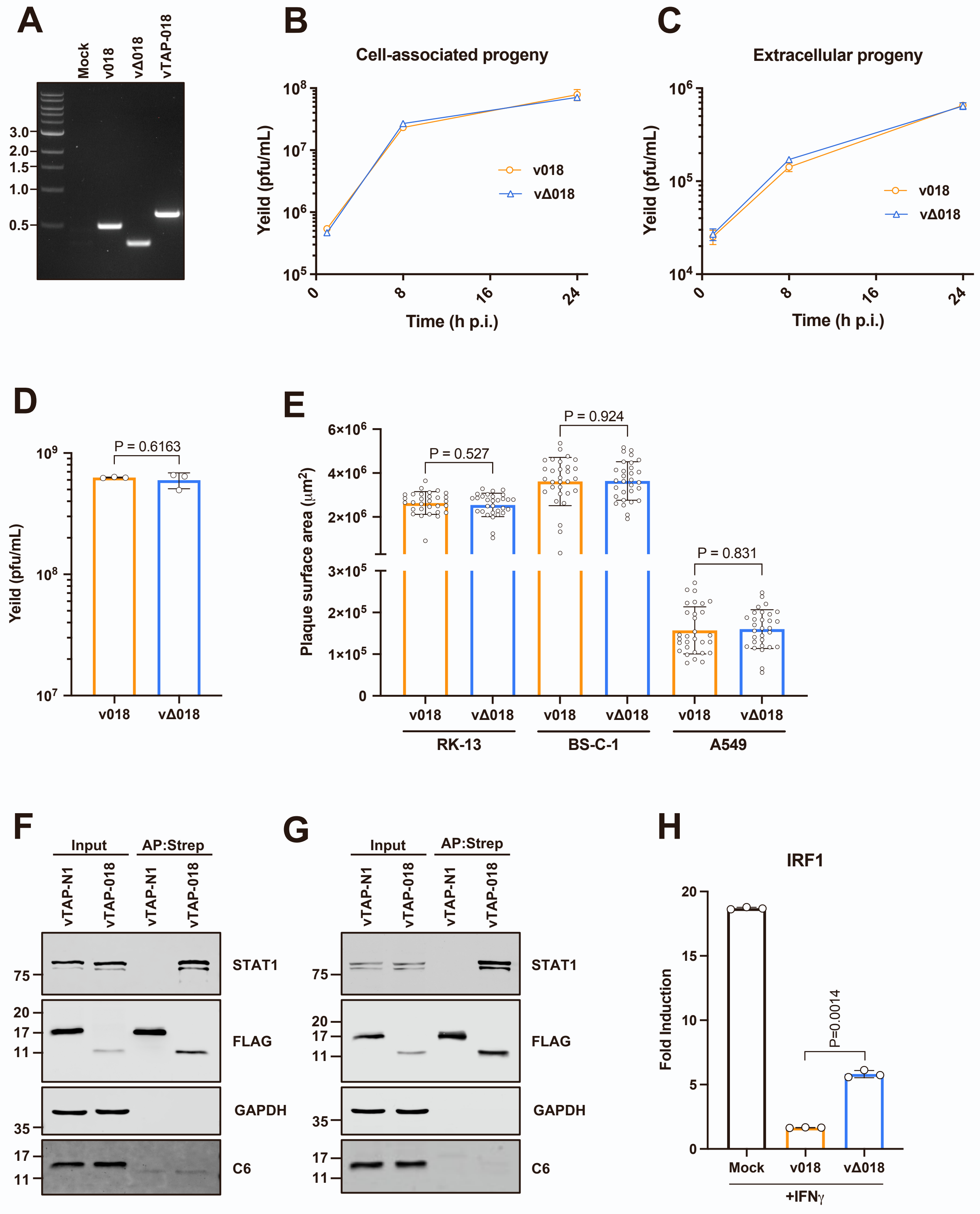

Figure S4. Characterisation of VACV-WR deletion mutant lacking gene *VACWR018*. Related to Figure 4. (**A**) PCR amplification of genomic DNA from the indicated viruses using primers upstream and downstream of the 018 ORF. The position of DNA size markers (kbp) are shown on the left side of the image. (**B, C**) BS-C-1 cells were infected with either v018 (orange) or vΔ018 (blue) at 5 pfu/cell. At 1, 8 and 24 h p.i. infectious virus titres associated with cells (**B**) and in the supernatants (**C**) was determined by plaque assay on BS-C-1 cells. Means ± SD (n=2 per condition) are shown and P=>0.05 for all timepoints. (**D**) A549 cells were infected with either v018 (orange) or vΔ018 (blue) at 5 pfu/cell. At 24 h p.i., cells and supernatant were collected, and infectious virus titres were determined by plaque assay on BS-C-1 cells. **(E)** RK13, BS-C-1 and A549 cells were infected at 30 pfu per well with either v018 (orange) or vΔ018

(blue). At 72 h p.i. monolayers were stained and plaque surface areas were quantified. Means ± SD (n=30 plaques per condition) are shown. (**F**) BS-C-1 cells or (**G**) MEFs were infected with vTAP-018 or vTAP-N1 at 5 pfu/cell for 12 h. TAP-tagged proteins were affinity-purified by Strep-Tactin and whole cell lysates (Input) and affinity-purified proteins (AP:Strep) were analysed by immunoblotting with α-FLAG, α-GAPDH, α-STAT1 and α-C6. (**H**) A549 cells were mock-infected or infected with v018 or vΔ018 at 10 pfu/cell. At 2 h p.i. cells were stimulated IFNγ (25 ng/mL) for 1 h. Total RNA was extracted and mRNA for IRF1 was analysed by RT-qPCR. Means ± SD (n=3 per condition) are shown. Data from (**B-H**) representative of 2 individual repeats. Significances were determined using Unpaired t-test with Welch's correction (**B-E**, **H**).

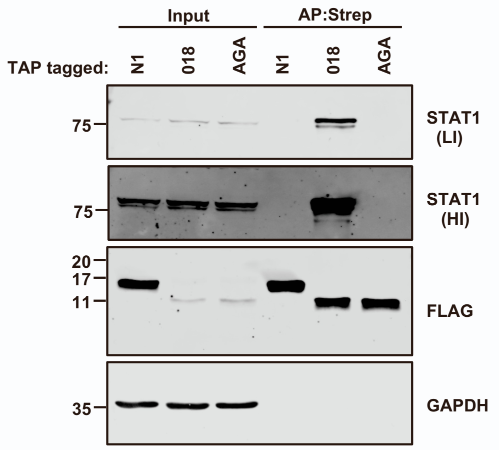

Figure S5. The 018 HxH motif is essential for 018 co-precipitation with STAT1 from cells. Related to Figure 6. TAP-tagged 018, 018 AGA (labeled AGA) and N1 were expressed by transfection in 2fTGH cells and affinity purified by Strep-Tactin. Whole cell lysates (Input) and affinity-purified proteins (AP:Strep) were analysed by immunoblotting with α-FLAG, α-GAPDH and α-STAT1. A high intensity (HI) and low intensity (LI) scan of α-STAT1 are shown. Data is representative of 2 independent repeats.

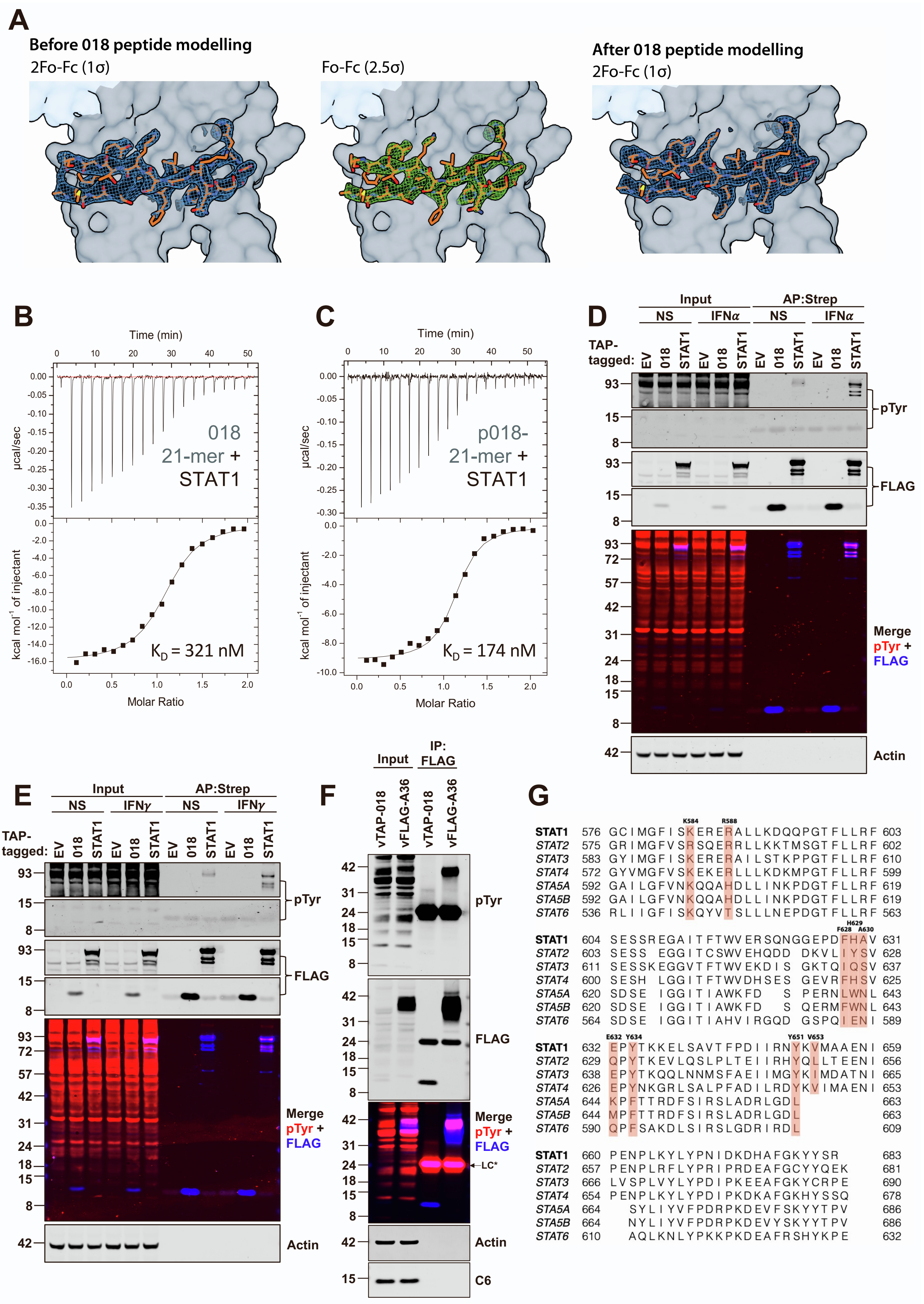

Figure S6. Analysis of the 018 pTyr-independent mode of STAT binding. Related to Figure 7. (**A**) Electron density maps of 018 peptide before and after peptide modelling and refinement. (**B-C**) ITC data for 100 µM 018 21-mer (**B**) and 100 µM p018 21-mer (pTyr29) (**C**) titrated into 10 µM U-STAT1. Fitting of the isotherm (bottom) to a one site model gave a K_D of 321 nM (**B**) and 174 nM (**C**). (**D-E**) EV,

TAP-tagged 018 and TAP-tagged STAT1 were expressed by transfection in HEK 293T cells and at 16 h post-transfection, cells were kept either non-stimulated (NS) or were stimulated with IFNα (1000 U/mL) (**D**), or IFNγ (25 ng/mL) (**E**) for 30 min and TAP-tagged proteins were affinity-purified by Strep-Tactin. Whole cell lysates (Input) and affinity-purified proteins (AP:Strep) were analysed by immunoblotting with α-pTyr, α-FLAG and α-actin. (**F**) A549 cells were infected with either vTAP-018 or vFLAG-A36 (a VACV protein phosphorylated on Tyr), at 5 pfu/cell and at 12 h p.i., TAP-tagged and FLAG-tagged proteins were immunoprecipitated using M2-FLAG affinity gel. Whole cell lysates (Input) and immunoprecipitated proteins (IP:FLAG) were analysed by immunoblotting with α-pTyr, α-FLAG, α-actin and α-C6. LC = IgG light chain. Data from (**D-F**) are representative of 2 individual repeats. (**G**) Alignment of STAT family SH2 domains with residues that form contacts with 018 in the 018:STAT1 crystal structure highlighted in orange.

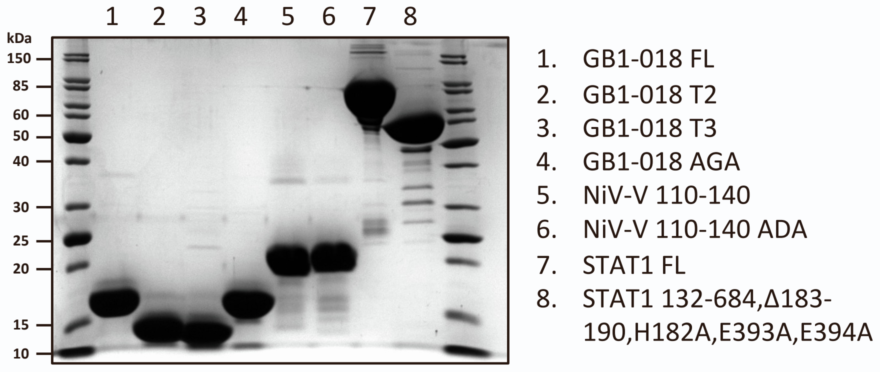

Figure S7. Coomassie-stained SDS-polyacrylamide gel of purified recombinant proteins. Related to STAR methods.
